# Clinical practice guidelines for the antenatal management of dichorionic diamniotic twin pregnancies: a systematic review

**DOI:** 10.1186/s12884-023-05652-z

**Published:** 2023-05-13

**Authors:** Caroline O’Connor, Emily O’Connor, Sara Leitao, Shauna Barrett, Keelin O’Donoghue

**Affiliations:** 1 INFANT Research Centre, University College Cork, Cork, Ireland; 2 Pregnancy Loss Research Group, Department of Obstetrics & Gynecology, University College Cork, Cork, Ireland; 3 National Perinatal Epidemiology Center (NPEC), University College Cork, Cork, Ireland; 4 Cork University Hospital Library, Cork University Hospital, Cork, Ireland; 5 Department of Obstetrics and Gynecology, Cork University Maternity Hospital, 5th Floor, Wilton Road, Cork, Ireland

**Keywords:** Dichorionic diamniotic, Twin pregnancy, Clinical practice guidelines, Antenatal management

## Abstract

**Objective:**

This review aimed to identify guidelines with recommendations applicable to the antenatal management of dichorionic diamniotic twin pregnancies within high-income countries, appraise their methodological quality, and discuss the similarities and variability across guidelines.

**Method:**

A systematic literature review of electronic databases was performed. Manual searches of guideline repositories and websites of professional organisations were performed to identify additional guidelines. The protocol for this systematic review was registered on PROSPERO (CRD42021248586, 25 June 2021). AGREE II and AGREE-REX tools were applied to assess the quality of eligible guidelines. A narrative and thematic synthesis described and compared the guidelines and their recommendations.

**Results:**

Twenty-four guidelines were included, from which 483 recommendations were identified across 4 international organisations and 12 countries. Guidelines addressed eight themes and recommendations were classified accordingly: chorionicity and dating (103 recommendations), fetal growth (105 recommendations), termination of pregnancy (12 recommendations), fetal death (13 recommendations), fetal anomalies (65 recommendations), antenatal care (65 recommendations), preterm labour (56 recommendations) and birth (54 recommendations). Guidelines showed significant variability in recommendations, with conflicting recommendations regarding non-invasive preterm testing, definitions surrounding selective fetal growth restriction, screening for preterm labour and the timing of birth. Guidelines lacked a focus on standard antenatal management of DCDA twins, management of discordant fetal anomaly and single fetal demise.

**Conclusions:**

Specific guidance for dichorionic diamniotic twins is overall indistinct and access to guidance regarding the antenatal management of these pregnancies is currently difficult. Management of discordant fetal anomaly or single fetal demise needs greater consideration.

**Supplementary Information:**

The online version contains supplementary material available at 10.1186/s12884-023-05652-z.

## Introduction

Twin pregnancies comprised 1.8% of all births in Ireland in 2019 and the twinning rate was 17.8 per 1,000 maternities [1]. There has been no significant change in the rate of twin pregnancies in Ireland since 2012, however, epidemiological data from the UK and US indicate a decline in multiple pregnancies, owing to improvements in assisted reproductive therapies (ART) and the implementation of single embryo transfer policies [2, 3]. Despite this decline twins accounted for 32.1 per 1000 births in the US and 15.3 per 1000 births in the UK in 2019 [4, 5]. Twin pregnancies are associated with an increased risk of maternal complications such as hypertensive disorders, and they contribute to an increased risk of perinatal morbidity and mortality, thereby causing significant resource implications for both healthcare providers and maternity services [6, 7]. Given the increased risk associated with twin pregnancies, there is a need for evidenced-based and up-to-date recommendations for clinical care [8].

One of the most important factors influencing perinatal outcomes of twin pregnancies is chorionicity [9]. Twin pregnancies can be dichorionic (70%) or monochorionic (30%), and should be managed differently according to this [9–11]. Monochorionic twin pregnancies have a higher rate of perinatal morbidity and mortality compared to dichorionic twin pregnancies, due to the presence of vascular anastomoses, resulting in specific conditions such as twin-to-twin transfusion syndrome [8, 12]. Monochorionic twin pregnancies are also associated with higher rates of preterm birth and low birthweight [13]. In recognition of these risk factors, specific monochorionic international clinical practice guidelines exist for their management [14–16]. Dichorionic twin pregnancies have an increased risk of perinatal mortality and morbidity compared to singleton pregnancies, which is largely attributed to increased rates of fetal growth restriction, preterm birth, and congenital anomalies [17, 18]. Therefore, increased fetal surveillance and recognition of risk factors is also warranted in these pregnancies [19, 20]. This systematic review focuses on dichorionic diamniotic (DCDA) twin pregnancies as they are high risk pregnancies compared to singletons, no guideline, to our knowledge, focuses solely on DCDA twin pregnancies, despite being the most prevalent type, and existing research supports the management of twin pregnancies according to chorionicity.

Clinical practice guidelines are defined as “systematically developed statements to assist practitioner and patient decisions about appropriate healthcare for specific clinical circumstances” [21, 22]. Clinical practice guidelines (guidelines or CPGs) are typically created by organisations, professional societies and/or scientific institutes to guide clinicians in their decision-making [8, 23]. There are national and international twin pregnancy CPGs, however, to our knowledge, no systematic review has examined the quality and the consistency of these guidelines. The identification, quality appraisal and review of published CPGs using a systematic method is a valuable step to standardise and optimise current DCDA twin pregnancy care.

This systematic review aims to identify, appraise, and examine published CPGs for the antenatal management of DCDA twin pregnancies, within high-income countries. Recommendations from the identified CPGs were also examined for a deeper understanding of the guidance provided concerning antenatal management of DCDA twin pregnancies.

## Methods

This systematic review is reported in accordance with the Preferred Reporting Items for Systematic Reviews and Meta-Analysis (PRISMA) criteria [24]. The protocol for this systematic review was registered on the International Prospective Register of Systematic Reviews (PROSPERO CRD42021248586, registered 25 June 2021) and published in an open research online platform [8].

### Eligibility criteria

For this review, clinical practice guidelines are “systematically developed statements informed by a systematic review of evidence and an assessment of the benefits and harms of alternative care options with the aim of optimizing patient care”, this definition is an adaptation of the definition from the Institute of Medicine (IOM) [25, 26]. The PICAR (population, interventions, comparators, attributes of eligible CPGs and recommendations) framework was used to develop the inclusion and exclusion criteria (Table 1) [27].

**Table 1:** Eligibility Criteria Pertaining to the Population and Clinical Areas, Interventions, Comparators, Attributes of Clinical Practice Guidelines and Recommendation Characteristics (Picar) Statement

PICAR Framework	Eligibility Criteria
**P**opulation, clinical indication(s), and condition(s).	**Study population** • Guidelines focused on the management of multiple pregnancies, in high income countries, containing recommendations applicable to the antenatal management of DCDA twin pregnancies • Humans only **Clinical Investigation** • Antenatal management of DCDA twin pregnancies **Clinical condition** • DCDA twin pregnancy is defined as each fetus having their own separate inner membrane (amnion) and outer membrane (chorion). For the purpose of this review, there is a specific focus on antenatal management of DCDA twin pregnancies. • The antenatal period extends from the start of pregnancy to the onset of labour
**I**nterventions	• Any intervention focusing on the antenatal management of DCDA twin pregnancies
**C**omparator(s), Comparisons, and (key) Content	• Any comparator or comparison • Key clinical practice guideline (CPG) content of interest is DCDA twin pregnancy antenatal management (including general twin pregnancy recommendations)
**A**ttributes of eligible CPGs	**Scope** • Cover any aspect of antenatal care for DCDA twin pregnancies. • Clinical practice guidelines only • Must be published **Language** • Full text CPGs in any language **Year of publication** • 2000 onwards **Developing/ publishing organisation** • Only CPGs issued or endorsed by national or international scientific societies, professional colleges, charitable organisations, and government organisations will be included. **Country of publication** • High income countries (83 countries), as defined by the world bank. This is due to the large discrepancies in care systems and management of care between high, low, and middle-income countries (25). **Version** • Current version only **Development process** • Evidence and/or consensus-based **Quality of evidence** • No maximum quality score based on the AGREE II and AGREE-REX instruments are required for eligibility. ALL relevant CPGs will be included in the review. • The quality score will be used to interpret the review findings and will be addressed in the discussion.
**R**ecommendations characteristics and ‘other’ considerations	• Guidelines must have a twin pregnancy or multiple pregnancy focus. • Eligible recommendations should be applicable to antenatal care of DCDA twin pregnancies. These include general recommendations for the management of a twin pregnancy and DCDA specific recommendations. Recommendations specific to monochorionic twin pregnancies, triplets or higher order multiples are not eligible for inclusion. • Recommendations will be considered if they are located anywhere within the guideline document (e.g. main text, algorithms, or tables). • Guidelines addressing any aspect of antenatal management of DCDA twin pregnancies without recommendations are eligible for inclusion.

### Information sources and search strategy

We systematically searched the following databases to identify eligible CPGs, published between 2000 and 2021: PubMed, EMBASE, Cumulative Index to Nursing and Allied Health Literature (CINAHL), MEDLINE, Web of Science and OpenGrey. Guideline repositories (n = 13) and the websites of professional organisations of high-income countries within the European Union (EU), Scandinavian countries and high-income English-speaking countries outside of the EU (n = 44). The search strategy was developed with the assistance of a specialist medical librarian. Information sources and search terms are detailed in Supplementary File 1 and in the published protocol [8].

### Study selection criteria

Retrieved records were imported into Mendeley, and deduplicated using the “check for duplicates” function. They were then imported into Rayyan and screened. Two independent reviewers (COC, EOC) screened 50% of the results each and cross-checked 20% of decisions, there were no discrepancies. The PICAR framework was applied broadly in the first phase of screening to ensure relevant CPGs were included. Monochorionic twin pregnancy guidelines were included in this review if they contained any recommendation applicable to a DCDA twin pregnancy, in particular dating and chorionicity recommendations. The blind function was activated for the reviewers on Rayyan during the full text screen to eliminate bias. Once completed the blind function was removed and any disagreements were discussed and resolved with the input of a third reviewer (KOD).

### Data collection process

To ensure CPGs were not missed during the professional organisation website search, relevant websites were contacted directly requesting any twin specific CPGs if none were publicly available prior to data extraction. Publication dates of the available CPGs were confirmed to ensure these were the most up to date versions available.

### Data extraction

Two reviewers performed single data extraction (July & August 2021), with verification of all data points to ensure accuracy and completeness. Translation for CPGs was done via Google Translate in German, Italian, Lithuanian, Romanian, and Danish [28]. The translated recommendations were sent to fluent speakers working in obstetrics, along with the google translate link and the original CPG. The fluent speakers were asked to verify the accuracy of the translated recommendations and correct them where necessary.

The data extraction spreadsheet was designed and adapted based on the methodology utilised in a previous systematic review of CPGs and based on the recommendations from Johnson et al. [27, 29]. To facilitate a thematic data synthesis, reviewers assigned each recommendation to one of eight categories during extraction (Table 2). The data extraction also captured the quality of evidence and strength of recommendations (if provided) for the recommendations, guideline title, authors, year of publication and the recommendation category specified within the guideline which helps to give context to the recommendation in some cases.

**Table 2:** Number of Recommendations by Category and Sub-Category

Category	Sub-Category	Number of recommendations	Number of CPGs
Chorionicity and dating (n = 103) Reference no. [9, 28-31, 33-45, 48-50].			21
Fetal growth (n = 105) Reference no. [9, 28-31, 33-35, 41-43, 45, 48-50].	Fetal growth charts Management Scanning	7 16 81	6 7 15
Termination of pregnancy (n = 12) Reference no. [9, 28, 29, 36, 41, 46, 50].	Multifetal pregnancy reduction (MFPR) Selective reduction	1 11	1 7
Fetal death (n = 13) Reference no. [9, 28, 34, 41, 48-50].	Management Counselling	12 1	6 1
Fetal anomalies (n = 75) Reference no. [9, 28-30, 33-35, 41, 42, 47-50].	Screening for aneuploidy Second trimester anomaly scan Counselling Management	52 9 8 6	13 7 5 4
Antenatal care (n = 65) Reference no. [28-31, 41-43, 48, 50]	General care Patient education Location of care Multidisciplinary team Organisation	29 16 6 13 1	8 6 4 6 1
Preterm labour (n = 56) Reference no. [9, 28-31, 31, 33, 34, 41-43, 48-50]	Intervention Screening	33 23	9 13
Birth (n = 54) Reference no. [28-33, 41-43, 48, 50]	Mode Timing	39 15	11 9

### Quality assessment

The CPGs were appraised using the Appraisal of Guidelines for Research and Evaluation Instrument (AGREE II) and the Appraisal of Guidelines for Research and Evaluation Instrument Recommendation Excellence (AGREE-REX) quality appraisal tools, which are internationally recognised gold standards for appraising guidelines [21, 25, 30]. AGREE II evaluates the overall methodological quality of the CPG, with a focus on the development process, while AGREE REX focuses specifically on the quality of the CPG recommendations, focusing on their credibility and implementability [25, 31, 32].

Domain scores were calculated by summing up all the scores of the individual items in a domain and by scaling the total as a percentage of the maximum possible score for that domain (as per AGREE II and AGREE REX user manuals). The AGREE II tool was applied to the overall CPG while the AGREE_REX tool was applied to a cluster of recommendations within a CPG that were specific to the antenatal management of DCDA pregnancies. AGREE-REX consists of two evaluation statements for each item, the first is a rating of the overall quality and the second is suitability for use. We did not include the optional ‘suitability for use’ score because the aim of this review is to compare, discuss and contrast recommendations within CPGs rather than determining whether the guideline recommendations are appropriate for use in a particular context. Two reviewers independently completed the AGREE II scores and a consensus-based approach was used between the two reviewers for AGREE-REX scoring. Consensus was reached through discussion, and involvement of a third party when required.

AGREE II and AGREE-REX outcomes are reported categorically using the five-point Likert scale described by other reviews, excellent (> 80%),  good  (> 60–80%),  average (> 40–60%), fair (> 20–40%) and poor (≤ 20%) [29, 33, 34].

### Data synthesis

A narrative and thematic synthesis was used to describe, compare, and contrast the CPGs and to extract the key themes within the recommendations specific to DCDA twin pregnancy.

## Results

### Guideline selection

**Fig. 1:**
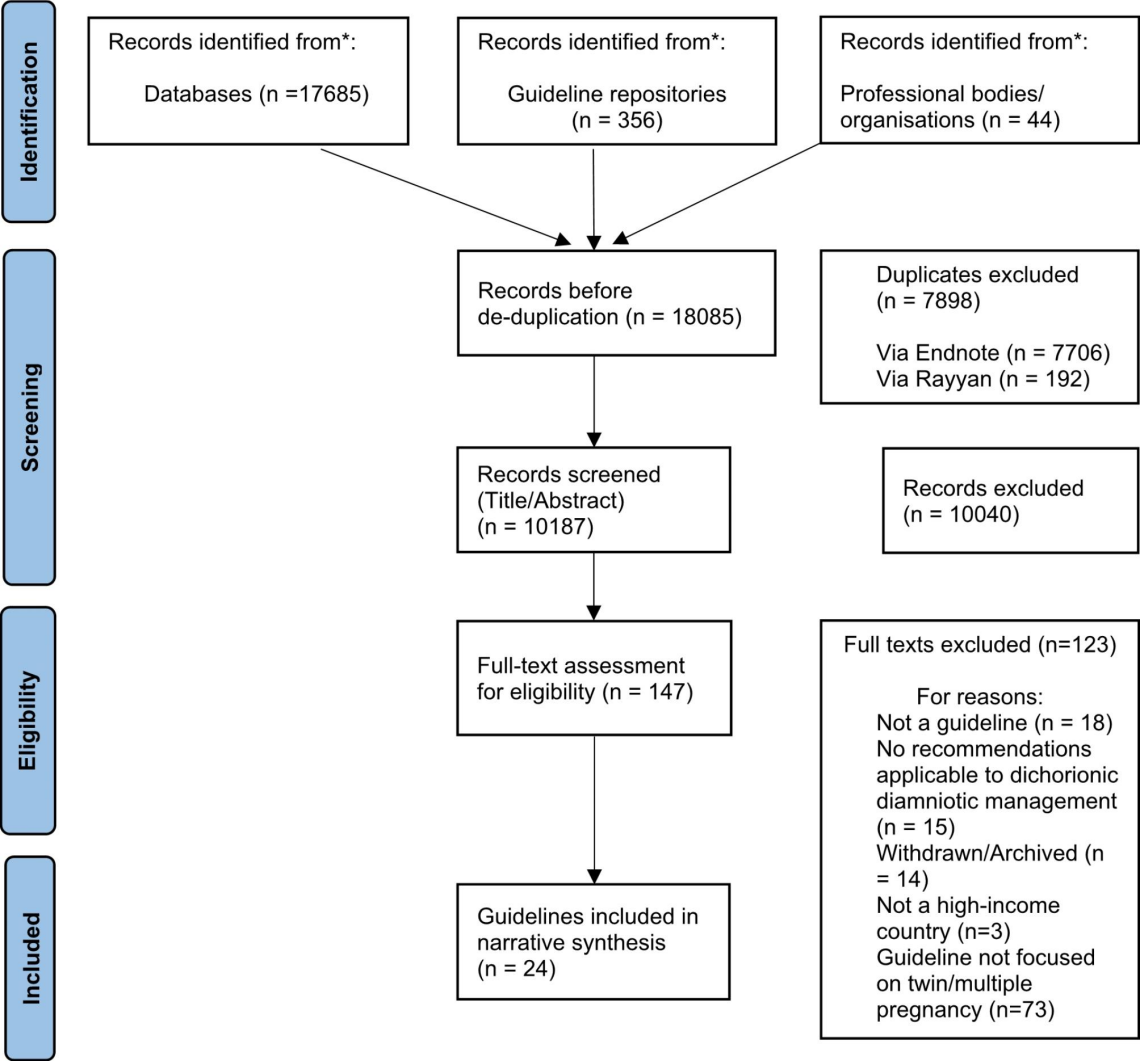
PRISMA flow diagram. *From*: Page MJ, McKenzie JE, Bossuyt PM, Boutron I, Hoffmann TC, Mulrow CD, et al. The PRISMA 2020 statement: an updated guideline for reporting systematic reviews. BMJ 2021;372:n71. doi: 10.1136/bmj.n71. For more information, visit: http://www.prisma-statement.org/.

A total of 18,085 records were retrieved overall from the database searches (n = 17,685), guideline repositories (n = 365) and professional bodies and organisations (n = 44). The full search list is included in Supplementary File 1. After the removal of duplicates (n = 7,898) and of documents deemed ineligible at title/abstract screening stage (n = 10,187), 147 records underwent full-text assessment. One hundred and twenty-three records were excluded following full-text screening. Twenty-four CPGs were included in the synthesis (Fig. 1).

### Guideline characteristics

Most included CPGs originated from international organisations (SMFM, FIGO, ISPD, ISUOG) (n = 6). The country-specific CPGs included: USA (n = 4), Canada (n = 3), Australia (n = 2), UK (n = 2), Denmark (n = 2), Ireland (n = 1), Italy (n = 1), France (n = 1) and Lithuania (n = 1). Two CPGs focused on more than one country, with one CPG covering Germany and Austria and one for Australia and New Zealand. A total of 18 CPGs were written in English, and the full translation from the French National College of obstetricians and gynaecologists (CNGOF) CPG was available via publication [35]. The remaining five CPGs were written in Italian, Lithuanian, Danish and German. Most CPGs were described as guidelines (n = 7) or clinical practice guidelines (n = 4) (Table 3). They varied in year of publication from 2010 to 2021.

**Table 3:** Summary of Included Clinical Practice Guidelines

Title	Year	Developing/publishing organisation and/or Lead authors	Funding	Country/countries of publication	Authors description of document (e.g. guideline/algorithm)	Type of guideline (formulated, adapted, updated, or revised)	No of recommendations specific to AN management of DCDA pregnancy	Development process (evidence based, consensus based or both)	System of rating evidence/ Quality instrument used during guideline development (GRADE, Oxford, not mentioned, or other)
Multifetal Gestations: Twin, Triplet, and Higher-Order Multifetal Pregnancies (Practice Bulletin No 231). (Reference no. 48)	2021	The American College of Obstetricians and Gynecologists (ACOG) and Society for Maternal-Fetal Medicine (SMFM)	Not specified	USA	Practice bulletin	Updated	28	Both	The U.S. Preventive Services Task Force (USPTF)
Multifetal Pregnancy Reduction. Committee Opinion No 719. (Reference no. 46)	2017	The American College of Obstetricians and Gynecologists (ACOG)	Not specified	USA	Committee opinion	Updated	1	Consensus based	Not stated
Management of Monochorionic Twin Pregnancy: Greentop Guideline No. 51. (Reference no. 40).	2016	Royal College of Obstetricians & Gynaecologists (RCOG)	Not specified	United Kingdom	Green-top Guideline	Updated	6	Evidence based	Scottish Intercollegiate Guidelines Network (SIGN)
ISUOG Practice Guideline: role of ultrasound in twin pregnancy. (Reference no. 9).	2016	The International Society of Obstetrics and Gynecology (ISUOG)	Not specified	International	Practice Guideline	Formulated	31	Both	Scottish Intercollegiate Guidelines Network (SIGN)
Ultrasound in twin pregnancies: SOGC Clinical practice guideline No. 260. (Reference no 34).	2011	Society of Obstetricians and Gynaecologists of Canada (SOGC)	Not specified	Canada	Clinical practice guideline	Formulated	19	Both	Canadian Task Force on Preventive Health Care
Prenatal Screening for and Diagnosis of Aneuploidy in Twin Pregnancies: Joint SOGC-CCMG Clinical Practice Guideline No, 262. (Reference no 36).	2011	Society of Obstetricians and Gynaecologists of Canada (SOGC) and the Prenatal Diagnosis Committee of the Canadian College of Medical Geneticists (CCMG)	Not specified	Canada	Clinical practice guideline	Updated	13	Both	Canadian Task Force on Preventive Health Care
Clinical practice guideline: Management of multiple pregnancy. (Reference no.43)	2012	Institute of Obstetricians and Gynaecologists, Royal College of Physicians of Ireland and Directorate of Strategy and Clinical Care, Health Service Executive	Not specified	Ireland	Clinical Practice Guideline	Formulated	11	Evidence based	Not specified
Twin nd Triplet Pregnancy: NG137. (Reference no.42).	2019	National Institute for Health and Care Excellence (NICE)	Not specified	United Kingdom	Guideline	Updated	60	Both	Not specified
Twin pregnancy. (Reference no.29).	2018	Department for Health and Ageing, Government of South Australia.	Not specified	South Australia	Perinatal Practice Guideline	Updated	31	Both	Not specified
Cell free (cf.)DNA screening for Down syndrome in multiple pregnancies. (Reference no. 47)	2021	International Society for Prenatal Diagnosis (ISPD)	Not specified	International	Position statement	Formulated	4	Evidence based	Not specified
SMFM Special Statement: State of the science on multifetal gestations: unique considerations and importance. (Reference no. 44).	2020	The Society of Maternal-Fetal Medicine published in the American Journal of Obstetrics and Gynecology	Not specified	International	Special statement	Formulated	1	Evidence based	Not specified
FIGO Committee Report. Good Clinical Practice advice: Management of Twin Pregnancy. (Reference no. 30)	2019	International Federation of Gynecology and Obstetrics (FIGO)	Not specified	International	Committee Report/Good clinical practice advice	Not specified	33	Both	Not specified
Role of ultrasonography in the management of twin gestation. (Reference no. 35).	2018	International Federation of Gynecology and Obstetrics (FIGO).	Not specified	International	Review article	Not specified	17	Evidence based	Not specified
American College of Radiology (ACR) Appropriateness Criteria: Multiple Gestations. (Reference no. 45)	2017	American College of Radiology (ACR)	Not specified	USA	Evidence-based Guidelines	Updated from 2012 version.	29	Both	RAND/UCLA Appropriateness Method and Grading of Recommendations Assessment, Development, and Evaluation or GRADE
RANZCOG Best Practice Statement: Management of monochorionic twin pregnancy. (Reference no. 39.)	2021	The Royal Australian and New Zealand College of Obstetricians and Gynaecologists (RANZCOG)	RANZCOG	Australia/New Zealand	Best practice statement	Updated	1	Both	National Health and Medical Research Council (NHMRC) Levels of Evidence and Grades of Recommendations for Developers of Guidelines
AWMF 015–087 S2e Überwachung und Betreuung von Zwillingsschwangerschaften (Monitoring and Care of Twin Pregnancies). (Reference no. 33).	2020	Arbeitsgemeinschaft der Wissenschaftlichen Medizinischen Fachgesellschaften (AWMF) (German Society for Gynecology & Obstetrics)	German Society for Gynecology and Obstetrics (DGGG), the German Society for Ultrasound in Medicine and the German Foundation for Women’s Health.	Germany	Guideline	Formulated	31	Evidence-based	Not stated
Tvillinger - håndtering af graviditet og fødsel (twins- handling pregnancy and childbirth). (Reference no. 31)	2010	(Sandbjerg) Dansk Selskab for Obstetrik og Gynækologi	German Society for Ultrasound in Medicine eV and the German Foundation for Women’s Health.	Denmark	Guideline	Updated	13	Evidence-based	Not stated
Tvillinger – Håndtering af fødslen (Twins- handling the birth). (Reference no. 32)	2020	Dansk Selskab for Obstetrik og Gynækologi	Not specified	Denmark	Guideline	Formulated	8	Evidence based	Not stated
Maternity- Management of monochorionic twin pregnancy. (Reference no. 37).	2020	New South Wales Government	Not specified	New South Wales Australia	Guideline	Formulated	1	Evidence based	Not stated
Ultrasound for twin and multiple pregnancies. (Reference no. 49)	2017	Alberta radiologists. Toward optimized practice (TOP)	Not specified	Alberta, Canada	Clinical Practice Guideline	Not stated	25	Both	Not stated
SMFM Special Statement: Updated checklist for management of monochorionic twin pregnancy. (Reference no. 38).	2020	Society for fetal maternal medicine (SMFM)	Not specified	International	Checklist	Prenatal care clinic	1	Consensus based	Not stated
Gestione della gravidanza multipla (Management of multiple pregnancy). (Reference no. 41).	2016	Confalonieri Ragonese Foundation, Societa’Italiana (SIGO), Associazione Ostetrici Ginecologi Ospedalieri Italiani (AOGOI) and Associazione Ginecologi Universitari Italiani (AGUI).	Not specified	Italy	Guideline	Formulated	45	Both	Not stated
Twin pregnancies: guidelines for clinical practice from the French College of Gynaecologists and Obstetricians (CNGOF). (Reference no. 28)	2009	La gynécologie et l’obstétrique en France - CNGOF-French national college of obstetricians and gynecologists.	Not specified	France	Guideline	Not explicitly stated but obstetricians and gynaecologists	38	Both	Not stated
Daugiavaisis Nėštumas (Multiple Pregnancy). (Reference no. 50).	2014	Lithuanian Society of Obstetricians and Gynaecologists, Lithuanian Midwives Association	Not specified	Lithuania	Guideline	Evidence based	36	Evidence based	Not stated

A total of 10 of the 24 CPGs were twin-specific focusing on the management of twin pregnancy (n = 6) [35–40], ultrasound in twin pregnancies (n = 3) [9, 41, 42] and prenatal screening in twin pregnancy (n = 1) [43]. No CPG focused specifically on DCDA twin management, however four focused on the management of monochorionic (MC) twin pregnancies [14–16, 44]. The remaining ten CPGs were broader in focus including triplet and higher order multiple pregnancies [45–54].

Fifteen CPGs (63%) did not report on the type of system used to rate the evidence justifying the recommendations whereas nine CPGs mentioned a system of rating evidence or quality instrument used during guideline development (37%). The system of rating/quality instruments used among included records are described in Table 3.

### Quality assessment findings (AGREE II and AGREE-REX evaluations)

**Fig. 2:**
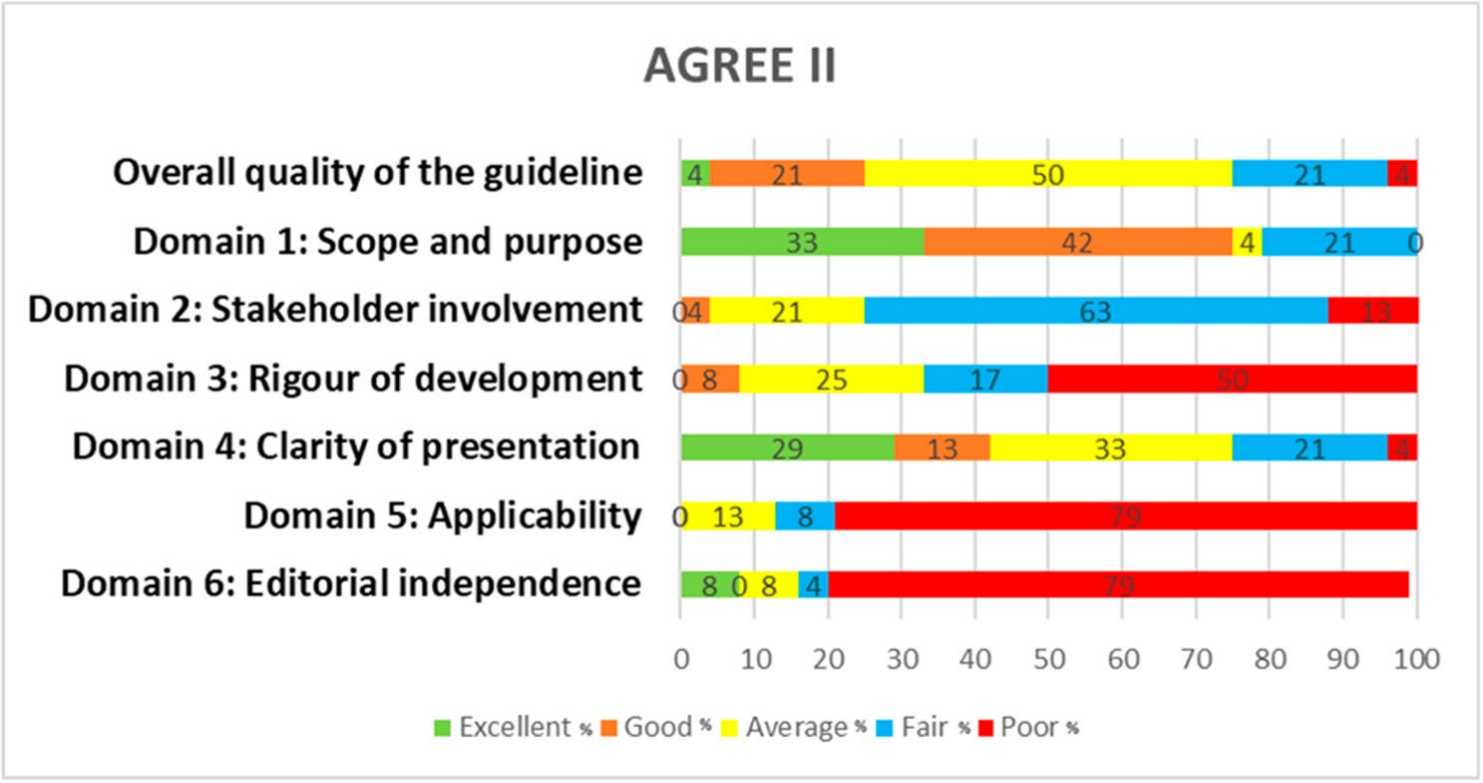
Agree II domain scores for the 24 guidelines, percentage (%). Excellent (> 80%), good (> 60–80%), average (> 40–60%), fair (> 20–40%) and poor (≤ 20%)

**Fig. 3:**
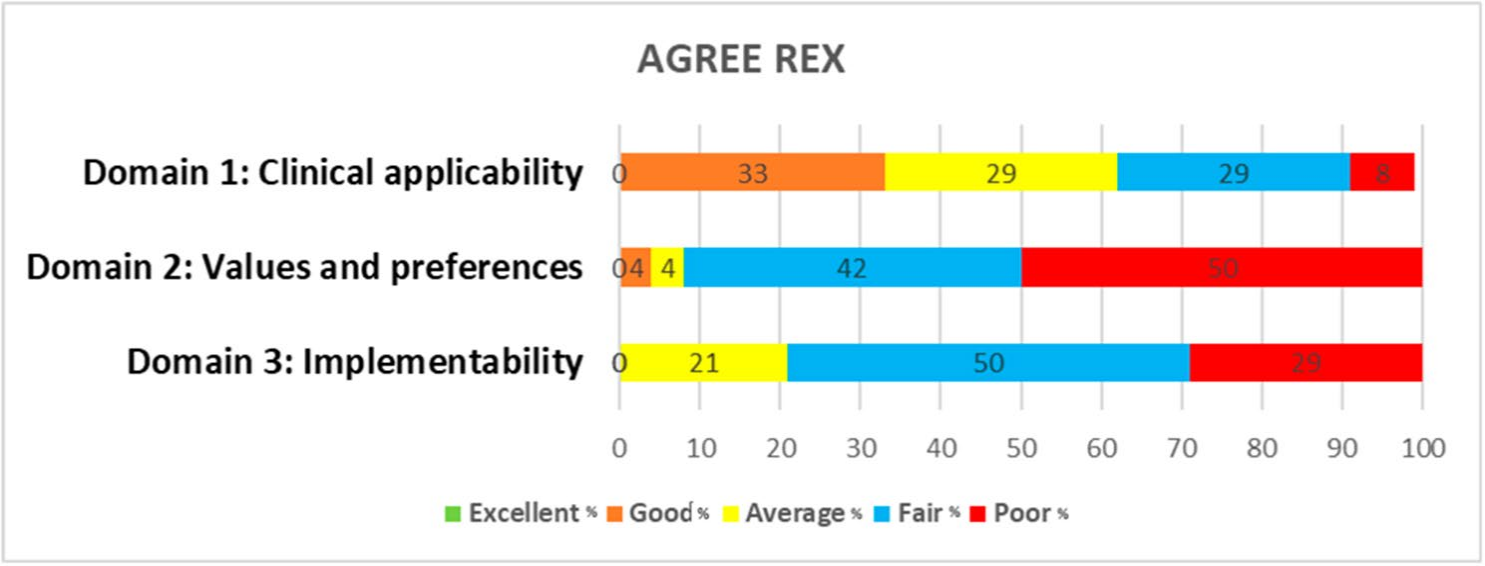
Agree -REX domain scores for the 24 guidelines, percentage (%). Excellent (> 80%), good (> 60–80%), average (> 40–60%), fair (> 20–40%) and poor (≤ 20%)

The quality assessment scores for the included CPGs are presented in Figs. 2 and 3; Tables 4 and 5. Following assessment of the CPGs quality using the AGREE II tool, only one CPG from the National Institute for Health Care Excellence (NICE), entitled ‘Twin and triplet pregnancy’ (NG137), was recommended for use in its current form by both reviewers [46]. Twenty-three CPGs were recommended for use with modifications. The highest average AGREE II domain score was scope and purpose, followed by clarity of presentation, editorial independence, rigour of development, stakeholder involvement and the domain scoring the lowest was applicability. Cohen’s K statistic was run to determine if there was agreement between the two assessors for the AGREE II scores. There was moderate agreement between the two researchers judgements, κ = 0.462, p < .001 [55].

**Table 4:** Agree II Quality Assessment by Domain (% Clinical Practice Guideline)

Title	Reference no:	Domain 1: Scope and purpose	Domain 2: Stakeholder involvement (%)	Domain 3: Rigour of development (%)	Domain 4: Clarity of presentation (%)	Domain 5: Applicability (%)	Domain 6: Editorial independence (%)	Overall quality (%)	Recommended for use
Toward Optimised Practice (TOP) Clinical practice guideline: Ultrasound for twin and multiple pregnancies (2017).	[48/51]	Good (69%)	Fair (25%)	Poor (0%)	Fair (33%)	Poor (0%)	Poor (0%)	Fair (25%)	YWM
ACOG- Multifetal Gestations: Twin, Triplet, and Higher Order Multifetal Pregnancies. Practice Bulletin No. 231 (2021).	[47]	Fair (39%)	Poor (17%)	Poor (20%)	Average (50%)	Poor (0%)	Poor (17%)	Average (50%)	YWM
ACOG- Multifetal pregnancy reduction. Committee opinion No. 719 (2017).	[45]	Excellent (86%)	Fair (36%)	Poor (10%)	Average (53%)	Poor (6%)	Poor (0%)	Average (50%)	YWM
ACR, Multiple Gestations (2017).	[44]	Good (72%)	Fair (39%)	Good (72%)	Excellent (81%)	Poor (0%)	Poor (13%)	Average (50%)	YWM
Überwachung und Betreuung von Zwillingsschwangerschaften (Screening, management and delivery in twin pregnancy) (2020).	[32]	Good (64%)	Average (47%)	Good (61%)	Excellent 83 (%)	Poor (8%)	Poor (8%)	Good (75%)	YWM
Grossesses gemellaires (Twin pregnancies): guidelines for clinical practice from the French College of Gynaecologists and Obstetricians (2019).	[27]	Good (67%)	Fair (39%)	Average (47%)	Average (42%)	Poor (4%)	Poor (17%)	Average (58%)	YWM
Tvillinger - håndtering af graviditet og fødsel (twins- handling pregnancy and childbirth) (2010).	[30]	Fair (39%)	Fair (25%)	Poor (19%)	Fair (36%)	Poor (0%)	Poor (0%)	Fair (33%)	YWM
Tvillinger – Håndtering af fødslen (Twins- handling the birth) (2020).	[31]	Fair (39%)	Fair (25%)	Fair (23%)	Average (47%)	Poor (0%)	Poor (0%)	Average (50%)	YWM
FIGO Committee Report: GCP advice, management of twin pregnancy (2019).	[29]	Fair (28%)	Fair (25%)	Poor (13%)	Fair (39%)	Poor (0%)	Poor (13%)	Fair (25%)	YWM
FIGO Role of ultrasonography in the management of twin gestation (2018).	[34]	Good (69%)	Fair (25%)	Poor (5%)	Fair (39%)	Poor (0%)	Fair (21%)	Average (42%)	YWM
Twin pregnancy, South Australia (2018).	[28]	Good (72%)	Fair (31%)	Poor (7%)	39 (Fair%)	Poor (0%)	Poor (0%)	Fair (25%)	YWM
HSE, Management of multiple pregnancy (2012).	[42]	Good (61%)	Fair (22%)	Fair (28%)	Average (42%)	Poor (13%)	Poor (0%)	Average (42%)	YWM
ISPD International Society for Prenatal Diagnosis Position Statement: cell free (cf.)DNA screening for Down syndrome in multiple pregnancies (2021).	[46]	Excellent (81%)	Average (56%)	Fair (21%)	Average (44%)	Poor (0%)	Poor (0%)	Average (50%)	YWM
ISUOG Practice Guideline: Role of ultrasound in twin pregnancy (2016).	[9]	Good (75%)	Average (44%)	Average44%)	Excellent (83%)	Poor (2%)	Poor (0%)	Average (58%)	YWM
Daugiavaisis nestumas (Multiple pregnancy) (2014).	[49]	Average (42%)	Poor (14%)	Poor (7%)	Average (42%)	Average (54%)	Poor (0%)	Average (42%)	YWM
NICE, Twin and Triplet Pregnancy (2019).	[41]	Excellent (89%)	Good (64%)	Average (59%)	Excellent81	Fair (27%)	Average (42%)	Excellent (83%)	Yes
New South Wales Government- Management of monochorionic twin pregnancy (2020).	[36]	Good (67%)	Fair (33%)	Poor (13%)	Average (47%)	Fair (21%)	Poor (0%)	Fair (33%)	YWM
RANZCOG Best Practice Statement: Management of monochorionic twin pregnancy (2021).	[38]	Excellent (94%)	Average (53%)	Average (47%)	Excellent (83%)	Poor (8%)	Excellent (83%)	Good (67%)	YWM
RCOG Green-top Guideline 51: Management of monochorionic twin pregnancy (2016).	[39]	Excellent (81%)	Average (47%)	Average (54%)	Excellent (89%)	Poor (19%)	Average (46%)	Good (67%)	YWM
Gestatione della gravidanza multipla (Management of multiple pregnancy) (2016).	[40]	Good (61%)	Fair (22%)	Fair (22%)	Good (75%)	Poor (6%)	Poor (0%)	Average (50%)	YWM
SMFM Special Statement: Updated checklists for management of monochorionic twin pregnancy (2020).	[37]	Excellent (83%)	Fair (36%)	Poor (20%)	Good (69%)	Average (56%)	Excellent (83%)	Average (50%)	YWM
SMFM Special Statement: State of the science on multifetal gestations: unique considerations and importance (2020).	[43]	Fair (36%)	Poor (14%)	Poor (18%)	Poor (8%)	Poor (8%)	Poor (1%)	Poor (8%)	YWM
SOGC- Guideline No. 262: Prenatal screening for diagnostics of aneuploidy in twin pregnancies (2011).	[35]	Excellent (86%)	Fair (36%)	Average (44%)	Excellent (81%)	Poor (0%)	Poor (13%)	Good (75%)	YWM
SOGC- Guideline No. 260: Ultrasound in twin pregnancies (2011).	[33]	Excellent (83%)	Fair (28%)	Average (50%)	Good (69%)	Poor (2%)	Poor (0%)	Good (67%)	YWM

^1^Recommended for use abbreviations: yes (Y), yes with modifications (YWM) and no (N)

**Table 5:** Agree-Rex Quality Assessment by Domain (% Clinical Practice Guideline)

Title	Reference no	Domain 1: Clinical applicability (%)	Domain 2: Values and preference (%)	Domain 3: Implementability (%)	Recommended for use^1^
Toward Optimised Practice (TOP) Clinical practice guideline: Ultrasound for twin and multiple pregnancies (2017).	[48/51]	Fair (33%)	Poor (4%)	Fair (44%)	YWM
ACOG- Multifetal Gestations: Twin, Triplet, and Higher Order Multifetal Pregnancies. Practice Bulletin No. 231 (2021).	[47]	Average (50%)	Fair (21%)	Poor (17%)	YWM
ACOG- Multifetal pregnancy reduction. Committee opinion No. 719 (2017).	[45]	Fair (33%)	Fair (21%)	Poor (8%)	YWM
ACR, Multiple Gestations (2017).	[44]	Good (72%)	Fair (25%)	Poor (17%)	YWM
Überwachung und Betreuung von Zwillingsschwangerschaften (Screening, management, and delivery in twin pregnancy) (2020).	[32]	Good (72%)	Fair (25%)	Fair (33%)	YWM
Grossesses gemellaires (Twin pregnancies): guidelines for clinical practice from the French College of Gynaecologists and Obstetricians (2019).	[27]	Average (50%)	Fair (25%))	Fair (33%)	YWM
Tvillinger - håndtering af graviditet og fødsel (twins- handling pregnancy and childbirth) (2010).	[30]	Average (44%)	Poor (4%)	Fair (25)	YWM
Tvillinger – Håndtering af fødslen (Twins- handling the birth) (2020).	[31]	Fair (39%)	Poor (8%)	Fair (25%)	YWM
FIGO Committee Report: GCP advice, management of twin pregnancy (2019).	[29]	Poor (17%)	Poor (8%)	Poor (17%)	YWM
FIGO Role of ultrasonography in the management of twin gestation (2018).	[34]	Fair (22%)	Poor (4%)	Poor (17%)	YWM
Twin pregnancy, South Australia (2018).	[28]	Fair (39%)	Poor (8%)	Poor (8%)	YWM
HSE, Management of multiple pregnancy (2012).	[42]	Average (44%0	Poor (8%)	Fair (25%)	YWM
ISPD International Society for Prenatal Diagnosis Position Statement: cell free (cf.)DNA screening for Down syndrome in multiple pregnancies (2021).	[46]	Fair (33%)	Poor (0%)	Fair (25%)	YWM
ISUOG Practice Guideline: Role of ultrasound in twin pregnancy (2016).	[9]	Good (78%)	Fair (21%)	Poor (17%)	YWM
Daugiavaisis nestumas (Multiple pregnancy) (2014).	[49]	Fair (39%)	Fair (25%)	Average (50%)	YWM
NICE, Twin and Triplet Pregnancy (2019).	[41]	Good (78%)	Good (63%)	Average (58%)	Yes
New South Wales Government- Management of monochorionic twin pregnancy (2020).	[36]	Average (42%)	Poor (0%)	Average (46%)	YWM
RANZCOG, Best Practice Statement: Management of monochorionic twin pregnancy (2021).	[38]	Average (50%)	Average (42%)	Average (50%)	YWM
RCOG, Green-top Guideline 51: Management of monochorionic twin pregnancy (2016).	[39]	Good (61%)	Fair (21%)	Fair (25%)	YWM
Gestatione della gravidanza multipla (Management of multiple pregnancy) (2016).	[40]	Good (61%)	Poor (17%)	Fair (33%)	YWM
SMFM Special Statement: Updated checklists for management of monochorionic twin pregnancy (2020).	[37]	Average (50%)	Fair (29%)	Average (50%)	YWM
SMFM Special Statement: State of the science on multifetal gestations: unique considerations and importance (2020).	[43]	Poor (17%)	Poor (0%)	Poor (0%)	YWM
SOGC-Guideline No. 262: Prenatal screening for diagnostics of aneuploidy in twin pregnancies (2011).	[35]	Good (78%)	Fair (21%)	Fair (25%)	YWM
SOGC- Guideline No. 260: Ultrasound in twin pregnancies (2011)	[33]	Good (61%)	Poor (17%)	Fair (25%)	YWM

^1^Recommended for use abbreviations: yes (Y), yes with modifications (YWM) and no (N)

Following the assessment of quality using the AGREE-REX tool, the majority of CPGs (n = 23) were recommended for use with modifications and only one CPG was recommended for use in their current form. This was the NICE guideline for ‘Twin and triplet pregnancy’ (NG137), [46]. The highest average AGREE-REX domain score was clinical applicability, followed by values and preferences, while implementability was the lowest scoring domain. Cohen’s kappa was not applicable to AGREE-REX because the two reviewers reached consensus on scores.

### Synthesis of recommendations

There were 483 recommendations extracted from the 24 CPGs. Each recommendation was classified according to the following 8 categories: Chorionicity and dating, Fetal growth, Termination of pregnancy, Fetal death, Screening for fetal anomalies, Antenatal care, Preterm labour and Birth (further detail on recommendations included in each category available in Supplementary Files 2–9). The eight categories were further broken down into subcategories to facilitate the discussion points of this systematic review. The number of recommendations by category and sub-category are presented in Table 3. Each of these categories and related recommendations are discussed further below.

#### Chorionicity and dating

A total of 103 recommendations from 21 CPGs were categorised under ‘chorionicity and dating’ (Supplementary File 2). The optimal gestational age for diagnosis of chorionicity was addressed in fifteen CPGs through 41 recommendations. The CPGs agreed chorionicity should be determined in early pregnancy but differed in their definition. Four CPGs provided a reference range which varied from 10 weeks to 13 + 6 weeks gestation [15, 40, 45, 54]. Eight CPGs stated chorionicity should be determined before 13 + 6 (n = 1), 14 (n = 6) and 15 weeks gestation (n = 1) [9, 14, 16, 38, 42, 46, 48]. The remainder varied between, “first trimester”, “first trimester and early second trimester”, “first consultation” and “as early as possible” [35, 36, 42, 43, 46, 49, 52].

Seven recommendations across six CPGs detailed the appropriate methods to determine chorionicity which included the number of placental masses, membrane thickness and identifying the T or lamba sign [9, 15, 35, 37, 38, 46, 53]. A total of 14 recommendations across 11 CPGs recommended procedures to maintain and document twin designation which covered nomenclature and description of twin fetuses, photographic records following chorionicity and the documentation strategy [9, 15, 36, 37, 40, 45–48, 53, 54].

Eleven recommendations across nine CPGs specified a management pathway in the event chorionicity cannot be determined [9, 15, 35, 40, 42, 45–47, 54]. The management pathways involved a second opinion in the form of a specialist (n = 2), specialised or tertiary centre (n = 2), senior sonographer (n = 1) and unspecified (n = 2) or assuming monochorionicity until proven otherwise (n = 4). Only one CPG contained 3 recommendations for staff training to facilitate identification of chorionicity at both the recommended gestational age and beyond, and clinical audits to evaluate the accuracy of staff in determining chorionicity [46].

In seven CPGs, 14 recommendations addressed the optimal timing for gestational age assessment and the method required for dating the pregnancy when the optimal timing for gestational age assessment had passed [9, 15, 36, 37, 40–42, 45, 46, 53]. One CPG stated dating is most accurate in the first trimester and 2 further CPGs were more precise with specified CRL 45-84 mm and between 11 + 0–13 + 6 weeks gestation, and another recommended dating from 7 weeks and beyond [9, 37, 41, 53]. There were five conflicting CPG recommendations regarding dating pregnancies following ART [9, 37, 40, 41, 45].

Three recommendations applied to dating a pregnancy when the optimal timing for dating had passed, two of these recommendations advised using the head circumference of the larger twin for dating pregnancies > 14 weeks gestation, and one recommended using a combination of parameters rather than a single parameter beyond the first trimester [9, 37, 41]. Eleven recommendations across eight CPGs specified the requirements for establishing gestational age when a discordance of size exists, with all agreeing the larger twin should be used for estimation of gestational age [9, 37, 40–42, 46, 53, 54].

#### Fetal growth

One hundred and five recommendations from fifteen CPGs were categorised under ‘fetal growth’ (Supplementary file 3). Eighteen of these recommendations (across 13 CPGs) described the frequency of fetal growth scans in uncomplicated DCDA pregnancy. The majority of growth scans were recommended every 3–4 weeks following the anomaly scan, while one CPG advised between 4 and 6 weeks and one CPG recommended weekly growth scans from 36 weeks gestation [9, 35–37, 40–42, 45–47, 52–54]. Two CPGs specified that there should be a minimum required number of appointments (8 appointments) throughout an uncomplicated DCDA twin pregnancy [54, 56].

Three CPGs defined selective fetal growth restriction (sFGR) and selective intrauterine growth restriction (sIUGR), two agreed in its definition as a condition in which one fetus has an estimated fetal weight < 10th centile and the intertwin estimated fetal weight discordance > 25% but differed between use of sFRG and sIUGR terms [9, 45]. Another guideline defined sFGR as a single estimated weight < 3rd percentile in a twin independent of chorionicity or in the case of DC twins sFGR diagnosis must meet two of three parameters [40]. Clear guidance was provided on the frequency of scans following sFGR diagnosis in 3 CPGs, however, while 2 guidelines stated that ultrasound scans are required every 2 weeks, another recommended scans on a weekly basis [9, 40, 46]. Two further CPGs recommended increasing surveillance following sFGR diagnosis [41, 52].

One CPG highlighted a 20% growth discordance as a marker to distinguish pregnancies at increased risk of adverse outcome [9]. One CPG defined FGR as ≥ 20% difference in the estimated fetal weight while 2 other CPGs defined growth discordance as the presence of either a > 20% difference in estimated fetal weight or an absolute measurement difference in abdominal circumference. Recommendations differed in the type of surveillance for growth discordance and included increased surveillance, umbilical artery Doppler assessment, and biophysical profile, cardiotocography (CTG), growth monitoring every 14 days, and scans every 1–2 weeks with AFI, UAD and biophysical profile [37, 39, 49, 53, 54].

Eighteen recommendations across eight CPGs were specific to the DCDA twin management following the identification of sFGR or discordant growth. Two CPGs supported the involvement of a fetal medicine specialist following identification of a first trimester CRL discordance >10% [9, 40]. Three CPGs supported seeking an underlying cause of the discordance with ISUOG detailing the search should include an anatomy scan, screening for viral infections, and an amniocentesis may be required to exclude chromosomal abnormalities [9, 37, 40]. AWFM agrees with ISUOG but does not detail the method for genetic testing and includes a family history and doppler sonography as part of the investigations [40].

Five CPGs made recommendations on procedures following identification of discordant growth which varied from referral to tertiary hospitals, to specialist services with adequate facilities, intense ultrasound monitoring without specifying the location, to the same or next day referral to a fetal medicine specialist to advise on and coordinate care [9, 35, 40, 45, 53]. Five CPGs recommend the appropriate calculation for EFW discordance and application of the same method to ascertain this (Supplementary File 3) [9, 37, 40, 42, 46]. The SOGC ultrasound in twin pregnancy guideline was the only guideline that provided a recommendation for defining oligohydramnios and polyhydramnios [41]. Three CPGs referred to the appropriateness of fetal echocardiography in DCDA twin pregnancies; no consensus was reached with one CPG stating it should not be offered while two CPGs recommended it could be considered in certain circumstances [38, 45, 49].

Seven recommendations over six CPGs gave guidance on the use of fetal growth charts. FIGO is the only CPG supporting the use of a twin specific growth chart because ‘it may reduce unnecessary medical intervention’, while three CPGs supported using singleton growth charts (9,37,40,53). Another CPG supported the use of the singleton growth chart but only until 32–35 weeks gestation [36]. Only one guideline specified the appropriate growth chart in the case of unknown gender (a singleton female growth chart is recommended) [53].

#### Termination of pregnancy

Twelve recommendations across seven CPGs were categorised to ‘termination of pregnancy’ (Supplementary File 4). Six recommendations address selective reduction. Two of the six recommendations specified with consensus, the optimal timing of selective reduction [40, 45]. Two CPGs described fetal reduction as being possible in cases of congenital anomalies but did not address the need for counselling or the benefits and risks of reduction [36, 40]. Two more CPGs outlined the importance of informing the couple on the risks and benefits of reduction but SOGC-CCMG is the only CPG to identify the need for counselling [43, 45]. Three recommendations were specific to selective termination of pregnancy. Two provide similar recommendations regarding the method of selective termination, with ISUOG detailing considerations and location of care surrounding second trimester or late selective termination in the third trimester [9, 40, 54]. One guideline recommended selective termination should be discussed and made available following counselling in the context of twins discordant for an abnormality [43]. Only one recommendation addressed the option of fetal reduction or termination in the case of congenital anomaly in one or both twins [36].

One recommendation was specific to a multifetal pregnancy reduction; this recommendation addressed patient counselling and the need for information in a timely and unbiased manner [50]. No recommendation addressed the need for training required, follow-up care of the women or her physical and psychosocial wellbeing.

#### Fetal death

Thirteen recommendations from seven CPGs were categorised under ‘fetal death’ (Supplementary File 5). Six recommendations across five CPGs recommended the transfer of care to tertiary centres or fetal medicine specialists in the case of a single fetal death [41, 45, 52, 53, 57] Only one recommendation from CNGOF addressed the need for counselling at the death of a twin [35]. The remaining seven recommendations addressed different aspects of maternal medical management and management of the surviving twin [45, 52–54]. Two guidelines addressed the need for prophylactic anti-D immunoglobulin administration, following the death of one twin, in women who are Rh negative and not isoimmunised [45, 54].

#### Screening for fetal anomalies

Seventy–five recommendations from fourteen CPGs were categorised under ‘screening for fetal anomalies’ (Supplementary File 6). One CPG was focused specifically on diagnosis of aneuploidy in twin pregnancies [43].

Fifty-two recommendations focused on screening for aneuploidy. Eight CPGs (fifteen recommendations) addressed screening for first trimester aneuploidy. Recommendations regarding the methods of appropriate screening were conflicting, three CPGs recommended the use of maternal age and nuchal translucency thickness (NT), or the combined test (NT, free beta-human chorionic gonadotropin (β-hCG) level and pregnancy–associated plasma protein-A (PAPP-A) level and SOGC-CCMG states this is the preferred method because the false-positive rate is decreased [9, 37, 40, 43], four CPGs recommended NT screening alone [36, 40, 41, 53], and one recommended the combined test alone [54]. One CPG recommended a calculation integrating the maternal age, NT of each fetus and the CRL [35]. One CPG recommended adjusting the serum analysis for twin pregnancies and another advised using the risk estimate tables for aneuploidy in singletons [35, 45].

Four CPGs (eight recommendations) advised on cell free DNA (cfDNA) screening [42, 43, 45, 51]. SIGO was the only CPG that did not support the use of cfDNA, reporting there is no sufficient data available [45]. The remaining recommendations supported the use of cfDNA testing and recommended when to offer it. One CPG advised invasive testing should be offered when cfDNA is not available [43].

CVS was identified as the preferred method of invasive testing in DCDA twins because it can be performed earlier than amniocentesis, allowing earlier diagnosis of aneuploidy; this was identified by CNGOF, ISUOG and AWMF and SIGO as being of importance, as the risk of selective reduction is lower in the first trimester compared to the second [9, 35, 37, 40, 45]. Only four CPGs recommended who should carry out the invasive test and all specify experienced staff are required to perform the procedure [35, 40, 45]. Eight recommendations (four CPGs) addressed the method of invasive testing. Two CPGs recommended sampling both fetuses in the case of DCDA twins while another stated routine sampling of both fetuses is not always necessary, however parental request justifies it [35, 40, 45]. SOGC recommended the preferred method of CVS for DCDA twins as a transabdominal and transcervical approaches or a transabdominal only approach, to minimise sampling errors however two other organisations just recommend the transabdominal route [35, 43, 45]. Only one CPG recommended the careful mapping of fetal and placental location prior to invasive testing [40].

One CPG highlighted in the case of fetal demise, anomaly or vanishing twin there is a significant risk of inaccurate test results following cfDNA or serum-based aneuploidy screening. Two further CPGs agreed that in the case of a vanishing twin, with a visible fetal pole on ultrasound, serum based analysis is not recommended, however, they differed in their following recommendations between NT alone or NT and maternal age [37, 45, 52]. One guideline differed in its approach to the use of serum-based screening in the case of vanishing twin and recommended maternal age, NT and serum-based screening without PAPP-A [40].

Six CPGs had recommendations specific to the second trimester anomaly ultrasound scan. Recommendations varied slightly between CPGs, one recommended routine anatomic evaluation is performed at the same gestational age applied to singletons, two recommended scanning between 18 and 22 weeks, with AWMF also recommending fetal echocardiography [37, 40, 41]. Three CPGs stated the gestational age should be between 18 and 20 weeks, and another recommended ‘in the second trimester’ [45, 46, 53, 54].

Only four CPGs provided recommendations regarding counselling when screening for anomalies [43, 45, 46, 52]. Six of the seven recommendations referred to counselling prior to screening, SOGC recommended it should be non-directive and respect the right of a woman to accept or decline any or all of the tests offered, while the NICE and SIGO recommendations focussed on sensitively explaining the aims and possible outcomes of all screening and diagnostics tests offered, including information regarding the complex decisions they might need to make based on the screening outcome [43, 45, 46]. ACOG’s recommendation focussed on counselling following a chromosomal abnormality diagnosis and advised discussion options for pregnancy management if only one fetus is affected [52].

#### Antenatal care

Sixty-five recommendations over nine CPGs addressed aspects of routine antenatal care in DCDA twins (Supplementary File 7). Only five CPGs provided recommendations on aspirin, one recommended low dose aspirin (LDA); specific dose recommendations varied from between 75 mg, 81 mg and 150 mg. All CPGs agree LDA should be prescribed in early pregnancy in the presence of other risk factors for pre-eclampsia [37, 38, 46, 52, 54]. Four CPGs focussed on recommendations regarding maternal weight gain and nutritional advice [35, 36, 46, 54]. These recommendations conflicted with one recommending diet, lifestyle and nutritional supplements as in routine singleton antenatal care and another recommending nutritional advice which might include supplementary iron and folate to accommodate the increased needs of a twin pregnancy [36, 46]. Only one CPG had a weight gain recommendation, but applicable to women with a normal body mass index [35]. Two guidelines highlighted the increased risk of anaemia in a twin pregnancy and recommended full blood counts at 20–24 weeks and between 28 and 34 weeks gestation [37, 46]. Three CPGs recommended a routine glucose tolerance test (GTT) in twin pregnancies, with only one specifying the recommended gestation [38, 45, 54]. One guideline stated that current data is insufficient to justify a recommendation for a GTT.

Only one CPG mentioned the administration of anti-D for Rh negative woman as per routine antenatal care [36]. One CPG advised increased monitoring for mental health complications in recognition of the increased incidence of postnatal depression in twin mothers, another recommended referring women with multiple pregnancies to additional support services but do not specify the services, and no other CPG focused on mental health as part of general antenatal care [36, 38]. Interestingly the South Australia CPG was the only one to recommend encouraging women with a multiple pregnancy to join a multiple birth specific association (South Australian Multiple Birth Association) [36].

FIGO were the only organisation to recommend a specialised twin clinic while CNGOF’s professional consensus states there is not enough evidence to justify a recommendation for a specialist twin clinic [35, 37]. One CPG provided detailed recommendations regarding the location and multidisciplinary team involvement for antenatal care [46]. Three CPGs recommended tertiary level care following identification of any complications [37, 45, 46].

Sixteen recommendations (five CPGs) focussed on patient education. NICE provided ten detailed recommendations regarding information on mode and timing of birth, risks, location of care and specify timelines for these conversations [46]. Four CPGs recommended women should be informed of the increased risk associated with twin pregnancy and one also recommended attending antenatal education specific to care and management of multiple birth [36, 37, 45, 54].

#### Preterm labour

Fifty-six recommendations (fourteen CPGs) specified views on screening and interventions for preterm labour (Supplementary File 8). There is no consensus across CPGs regarding the use of cervical length screening. Five guidelines recommended routine cervical length (CL) screening for twin pregnancies, however they differ in their definition of a short cervical length (20-25 mm) [9, 35, 38, 40, 42]. Four guidelines highlighted they do not advise routine cervical length screening [47, 52–54]. In women symptomatic of preterm labour one guideline recommended cervical length should not be used exclusively to direct the management [52].

One CPG recommended no intervention (CL, fetal fibronectin or any other rapid test) is appropriate for use in multiple pregnancy [35]. Three CPGs agreed it is not appropriate to use fetal fibronectin testing alone to predict the risk of spontaneous preterm birth [45, 46, 52].

No CPG supports the untargeted administration of corticosteroids and seven CPGs specified appropriate gestation for corticosteroid administration (24–34 weeks), with FIGO also recommending they should also be administered if there is a planned caesarean section < 37 weeks [36, 37, 45–47, 52]. ACOG and FIGO differ in their recommendations for repeat corticosteroid courses; FIGO recommended it should be individualised rather than routine practice but ACOG specify a single repeat course should be considered in women < 34 weeks, at risk of delivery within the next 7 days, who had their initial course > 14 days ago [37, 52].

CPGs agreed the use of prophylactic tocolysis is not routinely recommended, and two CPGs provided recommendations when a course of tocolysis may be considered [36, 52]. The use of progesterone, cervical pessary, cerclage and bed rest to prevent spontaneous preterm labour is not recommended in any included guideline. One guideline recommended the use of magnesium sulphate in the case of delivery prior to 32 weeks for fetal neuroprotection [52].

#### Birth

A total of fifty-four recommendations across eleven CPGs were applicable to this category (Supplementary File 9). Recommendations either addressed the mode of birth, the timing of birth and/or the multidisciplinary team required for the birth.

Nine CPGs recommended the timing of planned birth and there was no homogeneity amongst the recommendations, they differed as follows: 37 weeks (n = 1), from 37 weeks (n = 2), between 37 + 0–38 + 0 (n = 2), 38 weeks(n = 3), from 38 weeks and before 40 weeks (n = 1) [35–37, 39, 40, 45, 46, 52, 54]. The NICE guideline was the only guideline to address the possibility of a woman declining the recommended delivery date and recommended weekly appointments with an ultrasound for AFI, umbilical artery Doppler and a fortnightly growth scan in this instance [46].

Thirty-three recommendations across eleven guidelines addressed the mode of birth. Vaginal birth in the cases of one previous caesarean section and in the absence of other contraindications were supported in three guidelines [45, 52, 54]. Caesarean section delivery was only recommended in DCDA twin pregnancies in the case of a large head circumference, the first twin is not cephalic or in the case of major obstetric risk factors (not defined) [36, 37, 39, 46, 54]. The recommendations supporting vaginal birth were overall homogeneous with most supporting a vaginal birth where twin 1 is in the vertex position and in the absence of other complications and contraindications [35, 36, 38–40, 45, 46, 52, 54]. One CPG did not recommend one type of delivery rather than another, regardless of gestational age, if twin 1 is breech, or in the presence of uterine scars [35]. Two guidelines recommended individualised assessment for the mode of birth in the case of preterm labour before 26 weeks and in the case of pregnancies complicated by IUGR and fetal growth discordance [39, 46]. Four CPGs specifically highlighted the importance of an experienced obstetrician performing the delivery [35, 36, 47, 52].

## Discussion

This review identified 24 multiple pregnancy guidelines containing recommendations on the antenatal management of DCDA twin pregnancies within high-income countries. The majority of CPGs were from international organisations (n = 6) and written in English (n = 19). The focus of the CPGs included twins as well as higher order multiples, with no specific guidelines focussing specifically on DCDA twin management. There was high variability in the type and guidance provided in the CPGs and in their recommendations.

### Quality

The quality of the guideline methodology (AGREE II) varied greatly, and only one CPG was recommended for use in its current form [46]. The remaining guidelines were recommended for use, but with modifications. Overall, included CPGs recorded the lowest score in the applicability domain and editorial independence, this is consistent with findings from guidelines in other studies [29, 58]. Applicability in this case refers to consideration of the types of facilitators and barriers surrounding guideline implementation [36]. This suggests that few guidelines (less than 20% of those in our study) considered the implementation of recommendations in a clinical setting of DCDA twin management. The creation of guidelines is work-intensive and costly and their goal is to ultimately improve patient care, however if the implementation of guidelines is not considered during their development the guideline may not be translated into practice leading to potential ‘suboptimal patient outcomes or experiences and a waste of resources’ [30, 59].

In the editorial independence domain, guidelines should mention any relevant funding bodies and state that their views have not influenced guideline content, clarifying and addressing any potential conflict of interest between guideline developers [36]. In this SR many guidelines failed to prove editorial independence with 80% of CPGs scoring $\le 20\%$, suggesting many guidelines overlooked the importance of reporting on this domain. A guideline unable to prove editorial independence does not allow the user a fair assessment for potential conflicts of interest and may therefore raise concern regarding the credibility and transparency of the guideline.

The quality of the guideline recommendations within the CPGs (AGREE_REX) also varied greatly and only the recommendations from one CPG were endorsed for use in its current from [46]. The remaining guideline recommendations were endorsed for use with modifications. No CPG scored excellent in any of the 3 domains. CPGs scored highest in ‘clinical applicability’, followed by ‘implementability’ and scored the lowest in ‘values and preferences’. The values and preferences domain aims to incorporate the values and preferences of the target users, patients/populations, policy/decision-makers and guideline developers during the recommendation formulation. The majority of guidelines scored fair (40%) or poor (52%) in this domain, highlighting that the views and preferences of stakeholders were not adequately considered or explored when formulating DCDA twin antenatal management recommendations.

### Recommendations

The specific guidance for DCDA twins was at times indistinct and the guidelines lacked a clear standardised approach in both structure and recommendations. We identified differences in recommendations across CPGs, but we also found areas of consensus. The observed variations among recommendations may be explained by the year of guideline development, their PICAR/ PICO used during the search, method of rating the evidence and the guideline developers own prioritisation process in developing recommendations [58].

Findings from this review suggest that clinicians using national guidelines may be using guidelines that are not methodologically rigorous and/or reflect the best and most recent clinical evidence available. For example, most CPGs agreed chorionicity and dating was essential in early pregnancy but there was no consensus on the definition of early pregnancy, the best method of chorionicity determination or method of documentation, while the majority of CPGs agreed this was an important process. Given the high risk associated with twin pregnancies, research supports accurate dating, chorionicity assessment and documentation for correct management of the pregnancy [60]. Therefore, guideline recommendations should be more specific and the optimal gestation for dating and chorionicity should be agreed on and clarified on beyond ‘early pregnancy’.

Separating twin guidelines according to their chorionicity could be beneficial for clinical practice. We have seen this with monochorionic specific guidelines in recognition of the unique complications specific to monochorionic twins [61]. This is not the case with DCDA twins and the complexities of these twins merit greater recognition. Only 5 CPGs provided recommendations for dating a pregnancy following ART, this lack of consensus is surprising as in vitro fertilisation (IVF) greatly increases the risk of a twin pregnancy, especially in cases where more than one embryo is transferred [62].

Most of the recommendations focussed on fetal growth (n = 105), and we speculate the focus on this category is in recognition of twins having a higher rate of growth complications compared to singletons, which is generally managed by ultrasound surveillance [63]. There was consensus regarding the frequency of fetal growth scans across the guidelines, in recognition of the increased risk of DCDA twin pregnancies. Differences between recommendations were evident regarding the definition of sIUGR, sFGR and growth discordance, and these differences have also been identified in various previous reports and studies [12, 64]. Fetuses diagnosed with suboptimal fetal growth are at risk of stillbirth, preterm birth and long-term adverse health outcomes [65]. To facilitate the optimal management of these pregnancies, international consensus of standardised definitions would make guidelines more practicable and applicable [64].

No consensus was evident across guidelines on the frequency and exact type of ultrasound scans required following a complication regarding fetal growth, however recommendations regarding the location of care were all very similar, with tertiary hospitals and involvement of specialists recommended. Of the five guidelines addressing the use of fetal growth charts only one organisation recommended the use of a twin-specific growth chart [62]. Twins experience a slowing of growth from the third trimester compared to singletons and various research studies now support the use of twin-specific charts [66–68]. The argument in support of these charts is to decrease the rate of incorrect diagnosis of FGR, therefore, minimalizing medical intervention, without effecting outcomes [67, 68].

Very few CPGs addressed termination of pregnancy (n = 7) and only two recommendations endorsed the need for patient counselling following termination [43, 50]. Detailed counselling and emotional and psychological support are deemed as essential prerequisites in delivering this type of care [69]. Guidelines should focus further on the psychological needs of the woman in recognition the associated emotional and ethical difficulties around selective termination of pregnancy [69].

Similarly, very few guidelines specified recommendations regarding fetal death (n = 7), and of note only one recommendation addressed the need for bereavement counselling [35]. No recommendation addressed the need for staff education, follow-up care of the women or their physical and psychosocial wellbeing. Studies have highlighted patients who do not receive support to address common grief can experience complicated grief, leading to increased distress and complications [70–72]. Research also shows the important role healthcare professionals have during bereavement [71, 73]. Perinatal loss can effect one twin only and these bereaved families have a specific set of needs further to those of a singleton pregnancy [74]. Guidelines and organisations need to recognise and address these needs in order to provide optimal patient care. Healthcare professionals require specific training to facilitate the complexities of bereavement care and ideally organisations and health care systems would provide recommendations for training required [73].

The main goal of screening for fetal anomalies is to utilise information that will optimise the provision of antenatal care, achieving the best possible outcome for both the mother and baby [75]. This statement is also applicable to DCDA twin pregnancies because they are associated with a higher risk of aneuploidy compared to singletons [76]; however, there was no consensus on the recommended methods regarding screening for first trimester aneuploidy. There was no agreement on the optimal timing of the second trimester anomaly scan either. SIGO was the only CPG that did not support the use of cfDNA, however this guideline was published in 2016 and since then research has reviewed the screening performance of cfDNA in twin pregnancies [45]. The study completed in response to organisations not recommending cfDNA, supported its use in twin pregnancies and recommended it for consideration as a primary screening test for trisomy 21 [77]. Aside from this, eight CPGs mentioned methods of invasive testing and five CPGs identified CVS as the preferred choice for invasive testing in DCDA twins [9, 35, 37, 40, 45]. Recommendations lacked information on caring for the woman following a diagnosis of a fetal anomaly, with only one guideline including a recommendation on counselling and pregnancy management discussions following diagnosis of a chromosomal anomaly [52]. This review has highlighted that management of discordant twin anomaly or demise needs greater consideration within CPGs.

Recommendations regarding standard antenatal care for twin pregnancies is lacking from many guidelines and inconsistencies are observed on the topics covered among those that do include this topic. The antenatal care surrounding twin pregnancies can be challenging because both maternal wellbeing and the interest of both twins have to be managed [61]. There is an agreement across guidelines that DCDA twin pregnancies need increased antenatal visits, and although we recognise this is primarily to facilitate increased ultrasound surveillance, more emphasis should be placed on the routine antenatal care of women within twin guidelines. The NICE guideline is the most detailed in this category and provided 24 of the 65 recommendations [46].

Only two guidelines addressed the need for specialised twin clinics [35, 37] although one considered that there was a lack of evidence supporting a recommendation regarding these [35]. Literature surrounding twin pregnancy supports that they are at a higher risk compared to singletons [7, 61, 78–80]. Therefore, twin pregnancies are ideally managed by healthcare providers who are experienced in the management of twin pregnancies and trained to recognise the complications [81]. Recent evidence supports the use of specialised twin clinics for the antenatal care of women and showed that these can reduce the length of maternal inpatient stay, late prematurity, and reduce caesarean section rates without increasing maternal or neonatal adverse reactions [76, 82]. A 2016 Cochrane review urgently called for more studies addressing the use of specialised twin clinics [83]. Updated and new guidelines should reflect this most up-to-date research in support of specialised twin clinics.

Women are at an increased risk of preterm birth during twin pregnancy [84]. Therefore, they should be counselled on both risks and signs of preterm labour. However, only three recommendations highlighted women should be informed of the increased risk associated with twin pregnancies which include preterm birth [36, 46, 54]. No consensus was reached on screening for preterm labour or the routine use of cervical screening in twin pregnancies. However, studies show that in clinical practice, despite the conflicting guidelines, many specialists carry out routine cervical length screening [81, 85, 86], highlighting the importance of stakeholder involvement during guideline development to ensure it reflects best current practice. It also raises concern that guidelines regarding the management of twins has had limited impact [85].

Guidelines did not have consensus regarding the appropriate gestation for DCDA twin birth. Only one guideline addressed the possibility of a woman declining induction after reaching the organisation’s date for induction, which further supports this review’s findings regarding stakeholder involvement in guideline and recommendation development [46]. Homogeneity was evident across recommendations supporting vaginal birth in DCDA twin pregnancies and there was a focus on highlighting the importance of experienced obstetricians to attend the delivery, which implies recognition of the complications associated with twin delivery [87].

### Strengths and Limitations

This systematic review has several strengths. We conducted a rigorous and transparent search strategy and our search was not limited to English guidelines only. An internationally accepted gold standard tool was used for guideline appraisal of CPGs (AGREE II) and, as a complement AGREE_REX was used to assess the methodological quality of the guideline development and the quality of the recommendations specific to DCDA twin management.

## Conclusion

Overall specific guidance for DCDA twins is at times indistinct and the guidelines lack a clear standard approach in both structure and recommendations. Some organisations presented monochorionic twin-specific guidelines, with no acknowledgement of DCDA twin pregnancies. Management of discordant fetal anomaly or single fetal demise needs greater consideration within DCDA twin CPGs and the potential complexities of DCDA twins merit greater recognition. The quality of CPGs in their applicability and consideration of stakeholders needs improvement, particularly with the involvement of women who have experienced twin pregnancy.

Overall access to clear, focused and adequate guidance on the management of twin pregnancies is currently difficult. The lack of DCDA twin-specific clinical practice guidelines is a major gap that needs to be addressed.

## Electronic supplementary material

Below is the link to the electronic supplementary material.

**Additional file 1**

**Additional file 2**

**Additional file 3**

**Additional file 4**

**Additional file 5**

**Additional file 6**

**Additional file 7**

**Additional file 8**

**Additional file 9**

**Additional file 10**

## Data Availability

All data is included in this manuscript and within the supplementary (1–10) files attached.

## References

[1] Health Service Executive. Perinatal Statistics Report 2019. Healthcare Pricing Office (HPO), Health Service Executive (HSE); 2019.

[2] Niu M, Gao Y, Yang M, Zhang Y, Geng J, Song Z, et al. The quality and clinical applicability of recommendations in anxiety disorders guidelines: a systematic review of seventeen guidelines from seven countries. J Affect Disord. 2021 295(August):1301–9. 10.1016/j.jad.2021.08.103. 10.1016/j.jad.2021.08.103 34706444

[3] O’Farrel IB, Manning E, Corcoran P, White E, Greene. R on behalf of the PMG. Perinatal Mortality in Ireland Biennial Report 2018/2019. National Perinatal Epidemiology Centre 2021. doi:10.1007/BF02949658.

[4] Martin JA, Hamilton BE, Osterman MJK, Driscoll AK, Schwartz S, Horon I. Births: Final data for 2019. Natl Vital Stat Rep. 2021;70(2) p9-10. 33814033

[5] Office for National Statistics (2019). Birth characteristics in England and Wales: 2019. Stat Bull Office Natl Stat.

[6] Ferriman E, Stratton S, Stern V. Twin pregnancy. Obstetrics, Gynaecology and Reproductive Medicine. 2018;28(8):221–8. doi:10.1016/j.ogrm.2018.07.002.

[7] Khalil A, Liu B. Controversies in the management of twin pregnancy. Ultrasound in Obstetrics & Gynecology. 2021; 57(6):888–902. doi:10.1002/uog.22181. 10.1002/uog.22181 32799348

[8] O’Connor C, Leitao S, O’Donoghue K. A protocol for a systematic review of clinical practice guidelines for the antenatal management of dichorionic diamniotic twin pregnancy. HRB Open Research 2021; 4:115. doi:10.12688/hrbopenres.13418.1. 10.12688/hrbopenres.13418.1 PMC11170067 38873346

[9] Khalil A, Rodgers M, Baschat A, Bhide A, Gratacos E, Hecher K, et al. ISUOG Practice Guidelines: role of ultrasound in twin pregnancy. Ultrasound in Obstetrics & Gynecology. 2016; 47(2):247–63. 10.1002/uog.15821. 10.1002/uog.15821 26577371

[10] Carroll SGM, Tyfield L, Reeve L, Porter H, Soothill P, Kyle PM. Is zygosity or chorionicity the main determinant of fetal outcome in twin pregnancies? Am J Obstet Gynecol 2005; 193(3):757–61. doi:10.1016/j.ajog.2005.01.024. 10.1016/j.ajog.2005.01.024 16150271

[11] Hankins GVD, Saade GR. Factors influencing twins and zygosity. Paediatric and Perinatal Epidemiology. 2005 Jan;19(s1):8–9. doi:10.1111/j.1365-3016.2005.00609.x. 10.1111/j.1365-3016.2005.00609.x 15670115

[12] Lewi L (2020). Monochorionic diamniotic twins: what do I tell the prospective parents?. Prenat Diagn.

[13] Hack KEA, Derks JB, Elias SG, Franx A, Roos EJ, Voerman SK (2008). Increased perinatal mortality and morbidity in monochorionic versus dichorionic twin pregnancies: clinical implications of a large dutch cohort study. BJOG.

[14] The Royal Australian and New Zealand College of Obstetricians and Gynaecologists: RANZCOG. Management of Monochorionic Twin Pregnancy. BJOG 2017; 124(1):e1–45. doi:10.1111/1471-0528.14188.

[15] Royal College of Obstetricians & Gynaecologists. : RCOG. Management of Monochorionic Twin Pregnancy: Green-top Guideline No. 51. BJOG. 2017; 124(1):e1–45. doi:10.1111/1471-0528.14188. 10.1111/1471-0528.14188 27862859

[16] New South Wales Government. Guideline Maternity - Management of Monochorionic Twin Pregnancy Pregnancy. 2020.

[17] Glinianaia S V., Obeysekera MA, Sturgiss S, Bell R. Stillbirth and neonatal mortality in monochorionic and dichorionic twins: A population-based study. Human Reproduction. 2011;26(9):2549–57. 10.1093/humrep/der213. 10.1093/humrep/der213 21727159

[18] Vos AA, Denktaş S, Borsboom GJJM, Bonsel GJ, Steegers EAP (2015). Differences in perinatal morbidity and mortality on the neighbourhood level in dutch municipalities: a population based cohort study. BMC Pregnancy Childbirth.

[19] Al Dzajali M, Gajewska-Knapik K, Chong HP, Aviram A, Lipworth H, Asztalos EV et al. Trends in morbidity and mortality of extremely preterm multiple gestation newborns. Ultrasound Obstet Gynecol 2020; 54(1):263–71. doi:10.1542/peds.2014-4075.

[20] Chitrit Y, Filidori M, Pons JC, Duyme M, Papiernik E (1999). Perinatal mortality in twin pregnancies: a 3-year analysis in Seine Saint-Denis (France). Eur J Obstet Gynecol Reproductive Biology.

[21] Brouwers MC, Kho ME, Browman GP, Burgers JS, Cluzeau F, Feder G et al. AGREE II: Advancing guideline development, reporting, and evaluation in health care. Prev Med 2010 Nov;51(5):421–4. 10.1016/j.ypmed.2010.08.005. 10.1016/j.ypmed.2010.08.005 20728466

[22] Woolf SH, Grol R, Hutchinson A, Eccles M, Grimshaw J. Clinical guidelines: Potential benefits, limitations, and harms of clinical guidelines. BMJ. 1999 Feb 20;318(7182):527–30. 10.1136/bmj.318.7182.527. 10.1136/bmj.318.7182.527 PMC1114973 10024268

[23] Steeb T, Hayani KM, Förster P, Liegl R, Toussaint F, Schlaak M (2020). Guidelines for uveal melanoma: a critical appraisal of systematically identified guidelines using the AGREE II and AGREE-REX instrument. J Cancer Res Clin Oncol.

[24] Page MJ, McKenzie JE, Bossuyt PM, Boutron I, Hoffmann TC, Mulrow CD (2021). The PRISMA 2020 statement: an updated guideline for reporting systematic reviews. BMJ.

[25] AGREE-REX Research Team. (2019). The Appraisal of Guidelines Research & Evaluation—Recommendation EXcellence (AGREE-REX) [Electronic version]. [cited 2022 Jan 25]. Available from: chrome-extension: https://efaidnbmnnnibpcajpcglclefindmkaj/viewer.html?pdfurl=https%3A%2F%2Fwww.agreetrust.org%2Fwp-content%2Fuploads%2F2021%2F07%2FAGREE-REX-Tool-PDF-version.pdf&chunk=true.

[26] Field MJ, Lohr K (1990). Clinical practice guidelines: directions for a new program.

[27] Johnston A, Kelly SE, Hsieh SC, Skidmore B, Wells GA. Systematic reviews of clinical practice guidelines: a methodological guide. J Clin Epidemiol 2019 Apr 1;108:64–76. doi:10.1016/j.jclinepi.2018.11.030. 10.1016/j.jclinepi.2018.11.030 30529647

[28] Google. Google translate. Available from: https://translate.google.com/.

[29] Hennessy M, Dennehy R, Meaney S, Linehan L, Devane D, Rice R, et al. Clinical practice guidelines for recurrent miscarriage in high-income countries: a systematic review. Reprod Biomed Online. 2021 Jun;42(6):1146–71. 10.1016/j.rbmo.2021.02.014. 10.1016/j.rbmo.2021.02.014 33895080

[30] Gagliardi AR, Brouwers MC (2015). Do guidelines offer implementation advice to target users? A systematic review of guideline applicability. BMJ Open.

[31] Florez ID, Brouwers MC, Kerkvliet K, Spithoff K, Alonso-Coello P, Burgers J et al. Assessment of the quality of recommendations from 161 clinical practice guidelines using the Appraisal of Guidelines for Research and Evaluation–Recommendations Excellence (AGREE-REX) instrument shows there is room for improvement. Implementation Science. 2020 Dec 18;15(1):79. doi:10.1186/s13012-020-01036-5. 10.1186/s13012-020-01036-5 PMC7501649 32948216

[32] Brouwers MC, Spithoff K, Kerkvliet K, Alonso-Coello P, Burgers J, Cluzeau F (2020). Development and validation of a Tool to assess the Quality of Clinical Practice Guideline Recommendations. JAMA Netw Open.

[33] Eady EA, Layton AM, Sprakel J, Arents BWM, Fedorowicz Z, van Zuuren EJ. AGREE II assessments of recent acne treatment guidelines: how well do they reveal trustworthiness as defined by the U.S. Institute of Medicine criteria? British Journal of Dermatology. 2017 Dec 12;177(6):1716–25. doi:10.1111/bjd.15777. 10.1111/bjd.15777 28667760

[34] Daley B, Hitman G, Fenton N, Mclachlan S (2019). Assessment of the methodological quality of local clinical practice guidelines on the identification and management of gestational diabetes. BMJ Open.

[35] Vayssière C, Benoist G, Blondel B, Deruelle P, Favre R, Gallot D (2011). Twin pregnancies: guidelines for clinical practice from the French College of Gynaecologists and Obstetricians (CNGOF). Eur J Obstet Gynecol Reproductive Biology.

[36] Al-Kouatly HB, Skupski DW (2019). Twin pregnancy: South Australian Perinatal Practice Guideline. Curr Opin Obstet Gynecol.

[37] Di Renzo GC, Gratacos E, Kurtser M, Malone F, Nambiar S, Sierra N (2019). Good clinical practice advice: management of twin pregnancy (FIGO committee report). Int J Gynecol Obstet.

[38] Lauritzen J, Christensen J, Clausen T, Hoseth E, Johansen M, Rohde M et al. Tvillinger – håndtering af graviditet og fødsel - Sandbjerg 2010. 2010;1–52.

[39] Schou KV, Lotte L, Andersen T, Storgaard L, Beck M, Sundtoft I et al. Tvillinger – Håndtering af fødslen. 2020.

[40] von Kaisenberg C, Klaritsch P, Ochsenbein-Kölble N, Hodel ME, Nothacker M, Hecher K. Überwachung und Betreuung von Zwillingsschwangerschaften. 2021. Available from: http://www.thieme-connect.de/DOI/DOI?10.1055/a-1248-8896doi:10.1055/a-1248-8896. 10.1055/a-1248-8896 33017845

[41] Morin L, Lim K, Bly S, Butt K, Cargill YM, Davies G (2011). Ultrasound in Twin Pregnancies: SOGC Clinical Practice Guideline No. 260. J Obstet Gynecol Can.

[42] Smith J, Treadwell MC, Berman DR. Role of ultrasonography in the management of twin gestation: FIGO. Int J Gynecol Obstet. 2018 Jun;141(3):304–14. 10.1002/ijgo.12483. 10.1002/ijgo.12483 29536536

[43] Audibert F, Gagnon A, Douglas Wilson R, Audibert F, Blight C, Brock JA, et al. Prenatal screening for and diagnosis of Aneuploidy in Twin Pregnancies no 262. J Obstet Gynecol Can. 2011 Jul;33(7):754–67. 10.1016/S1701-2163(16)34963-5. 21749753

[44] Hoskins IA, Combs CA (2020). Society for maternal-fetal Medicine Special Statement: updated checklists for management of monochorionic twin pregnancy. Am J Obstet Gynecol.

[45] Della G, Multipla G, Placido G, De, Dubini V, Stola E, Venturini PL et al. Gestione della gravidanza multipla. 2020;1–38.

[46] National Institute for Health and Care Excellence (NICE). Twin and triplet pregnancy. NICE guidelines (NG 137). 2019;(September):1–61.

[47] Institute of Obstetricians and Gynaecologists. Royal College of Physicians of Ireland (RCPI) and directorate of Strategy and Clinical Care HSE (HSE). Clinical practice guideline no. Volume 14. Management of Multiple Pregnancy; 2014.

[48] Grantz KL, Kawakita T, Lu YL, Newman R, Berghella V, Caughey A. SMFM Special Statement: state of the science on multifetal gestations: unique considerations and importance. Am J Obstet Gynecol. 2019 Aug;221(2):B2–12. 10.1016/j.ajog.2019.04.013. 10.1016/j.ajog.2019.04.013 PMC7556908 31002766

[49] Glanc P, Nyberg DA, Khati NJ, Deshmukh SP, Dudiak KM, Henrichsen TL (2017). ACR appropriateness Criteria® multiple gestations. J Am Coll Radiol.

[50] OBIČAN S, BROCK C, BERKOWITZ R, WAPNER RJ. Multifetal pregnancy reduction: ACOG Committee opinion no 719. Clin Obstet Gynecol. 2015 Sep;58(3):574–84. 10.1097/GRF.0000000000000119. 10.1097/GRF.0000000000000119 26083128

[51] Palomaki GE, Chiu RWK, Pertile MD, Sistermans EA, Yaron Y, Vermeesch JR et al. International Society for Prenatal Diagnosis (ISPD) Position Statement: cell free (cf) DNA screening for Down syndrome in multiple pregnancies. Prenatal Diagnosis 2021 Sep 15;41(10):1222–32. doi:10.1002/pd.5832. 10.1002/pd.5832 33016373

[52] The American College of Obstetricians and Gynecologists ACOG. Multifetal Gestations: Twin, Triplet, and Higher-Order Multifetal Pregnancies: Practice Bulletin No 231. Obstetrics & Gynecology. 2021 Jun;137(6):e145–62. doi:10.1097/AOG.0000000000004397. 10.1097/AOG.0000000000004397 34011891

[53] Toward Optimized Practice (TOP) Ultrasound Working Group. Ultrasound for twin and multiple pregnancies clinical practice guideline. Edmonton, AB: Toward Optimized Practice. 2016. Available from: http://www.topalbertadoctors.org.

[54] Lietuvos akušerių ginekologų. draugija (LAGD). Daugiavaisis Nėštumas. 2014.

[55] Laerd Statistics. Cohen’s Kappa. Available from: https://statistics.laerd.com/premium/spss/ck/cohens-kappa-in-spss.php.

[56] National Institute for Health and Care Excellence. Multiple pregnancy: twin and triplet pregnancies Quality Standard: (QS46). 2019.

[57] Cheong-See F, Schuit E, Arroyo-Manzano D, Khalil A, Barrett J, Joseph KS et al. Prospective risk of stillbirth and neonatal complications in twin pregnancies: Systematic review and meta-analysis. BMJ 2016 Sep 6;354:i4353. doi:10.1136/bmj.i4353. 10.1136/bmj.i4353 PMC5013231 27599496

[58] Jolliffe L, Lannin NA, Cadilhac DA, Hoffmann T. Systematic review of clinical practice guidelines to identify recommendations for rehabilitation after stroke and other acquired brain injuries. BMJ Open. 2018 Feb 1;8(2):18791. doi:10.1136/bmjopen-2017-018791. 10.1136/bmjopen-2017-018791 PMC5855444 29490958

[59] Burls A (2010). AGREE II - improving the quality of clinical care. The Lancet.

[60] Townsend R, Khalil A (2018). Ultrasound surveillance in twin pregnancy: an update for practitioners. Ultrasound.

[61] Lewi L, Deprest J. Management of twin pregnancies: where do we go from here? Ultrasound in Obstetrics & Gynecology. 2013 Jun;41(6):601–4. doi:10.1002/uog.12502. 10.1002/uog.12502 23712884

[62] Luke B (2017). Pregnancy and birth outcomes in couples with infertility with and without assisted reproductive technology: with an emphasis on US population-based studies. Am J Obstet Gynecol.

[63] Stirrup OT, Khalil A, D’Antonio F, Thilaganathan B (2015). Fetal growth reference ranges in twin pregnancy: analysis of the Southwest Thames Obstetric Research Collaborative (STORK) multiple pregnancy cohort. Ultrasound Obstet Gynecol.

[64] Unterscheider J, O’Donoghue K, Malone FD (2014). Guidelines on fetal growth restriction: a comparison of recent national publications. Am J Perinatol.

[65] Easter SR, Eckert LO, Boghossian N, Spencer R, Oteng-Ntim E, Ioannou C (2017). Fetal growth restriction: case definition & guidelines for data collection, analysis, and presentation of immunization safety data. Vaccine.

[66] Proctor LK, Kfouri J, Hiersch L, Aviram A, Zaltz A, Kingdom J (2019). Association between hypertensive disorders and fetal growth restriction in twin compared with singleton gestations. Am J Obstet Gynecol.

[67] Kalafat E, Sebghati M, Thilaganathan B, Khalil A, Bahamie A, Bhide A (2019). Predictive accuracy of Southwest Thames Obstetric Research Collaborative (STORK) chorionicity-specific twin growth charts for stillbirth: a validation study. Ultrasound Obstet Gynecol.

[68] Kibel M, Kahn M, Sherman C, Kingdom J, Zaltz A, Barrett J (2017). Placental abnormalities differ between small for gestational age fetuses in dichorionic twin and singleton pregnancies. Placenta.

[69] Beriwal S, Impey L, Ioannou C (2020). Multifetal pregnancy reduction and selective termination. The Obstetrician & Gynaecologist.

[70] Nuzum D, Meaney S, O’Donoghue K (2018). The impact of stillbirth on bereaved parents: a qualitative study. PLoS ONE.

[71] O’Connell O, Meaney S, O’Donoghue K (2016). Caring for parents at the time of stillbirth: how can we do better?. Women Birth.

[72] McSpedden M, Mullan B, Sharpe L, Breen LJ, Lobb EA (2017). The presence and predictors of complicated grief symptoms in perinatally bereaved mothers from a bereavement support organization. Death Stud.

[73] Leitao S, Helps A, Cotter R, O’Donoghue K (2021). Development and evaluation of TEARDROP - a perinatal bereavement care training programme for healthcare professionals. Midwifery.

[74] Richards J, Graham R, Embleton ND, Campbell C, Rankin J (2015). Mothers’ perspectives on the perinatal loss of a co-twin: a qualitative study. BMC Pregnancy Childbirth.

[75] Salomon LJ, Alfirevic Z, Audibert F, Kagan KO, Paladini D, Yeo G, et al. ISUOG consensus statement on the impact of non-invasive prenatal testing (NIPT) on prenatal ultrasound practice. Ultrasound in Obstetrics & Gynecology. 2014 Jul;44(1):122–3. 10.1002/uog.13393. 10.1002/uog.13393 24895295

[76] Bricker L (2014). Optimal antenatal care for twin and triplet pregnancy: the evidence base. Best Pract Res Clin Obstet Gynecol.

[77] Khalil A, Archer R, Hutchinson V, Mousa HA, Johnstone ED, Cameron MJ (2021). Noninvasive prenatal screening in twin pregnancies with cell-free DNA using the IONA test: a prospective multicenter study. Am J Obstet Gynecol.

[78] Khalil A, O’Brien P. Challenges of twin pregnancy. Br J Midwifery 2007 May;15(5):266–72. doi:10.12968/bjom.2007.15.5.23398.

[79] Lam JR, Liu B, Bhate R, Fenwick N, Reed K, Duffy JMN (2019). Research priorities for the future health of multiples and their families: The Global Twins and Multiples Priority setting Partnership. Ultrasound Obstet Gynecol.

[80] Kilby MD, Gibson JL, Ville Y (2019). Falling perinatal mortality in twins in the UK: organisational success or chance?. BJOG An International Journal Obstetrics Gynaecology.

[81] Hiersch L, Attali E, Melamed N. Special considerations regarding antenatal care and pregnancy complications in dichorionic twin pregnancies. Am J Obstet Gynecol MFM. 2021 Oct;100500. 10.1016/j.ajogmf.2021.100500. 10.1016/j.ajogmf.2021.100500 34637959

[82] Bhide A, Thilaganathan B (2004). What prenatal diagnosis should be offered in multiple pregnancy?. Best Pract Res Clin Obstet Gynecol.

[83] Dodd JM, Dowswell T, Crowther CA. Specialised antenatal clinics for women with a multiple pregnancy for improving maternal and infant outcomes. Cochrane Database of Systematic Reviews. 2015;2015(11). 10.1002/14651858.CD005300.pub4. 10.1002/14651858.CD005300.pub4 PMC8536469 26545291

[84] Geisler ME, O’Mahony A, Meaney S, Waterstone JJ, O’Donoghue K (2014). Obstetric and perinatal outcomes of twin pregnancies conceived following IVF/ICSI treatment compared with spontaneously conceived twin pregnancies. Eur J Obstet Gynecol Reproductive Biology.

[85] Gent J, Nanda S, Khalil A, Sharp A (2020). Antenatal management of multiple pregnancies within the UK: a survey of practice. Eur J Obstet Gynecol Reproductive Biology.

[86] Lipworth H, Hiersch L, Farine D, Barrett JFR, Melamed N (2021). Current practice of maternal–fetal medicine specialists regarding the Prevention and Management of Preterm Birth in Twin Gestations. J Obstet Gynecol Can.

[87] Barrett JFR. Twin delivery: Method, timing and conduct. Best Practice Research Clinical Obstetrics Gynaecology. 2014 Feb;28(2):327–38. doi:10.1016/j.bpobgyn.2013.12.008. 10.1016/j.bpobgyn.2013.12.008 24445020

