# Supplementary material for: Clinical practice guidelines for the antenatal management of dichorionic diamniotic twin pregnancies: a systematic review
Source: BMC Pregnancy Childbirth. 2023 May 13;23:347. doi: 10.1186/s12884-023-05652-z (PMC10182673; doi:10.1186/s12884-023-05652-z)
Supplement: Supplementary file 1 — Additional file 1 [file 12884_2023_5652_MOESM1_ESM.docx]

**SUPPLEMENTARY FILE 1 INFORMATION SOURCES AND SEARCH TERMS APPLIED**

|  | **Database: CINHAL**  **Date of least search:** 01/07/2021 | |
| --- | --- | --- |
|  | TX ALL TEXT | “Twin pregnanc*” |
|  | TX ALL TEXT | “Multifetal gestation*” |
|  | TX ALL TEXT | “Multifetal pregnanc*” |
|  | TX ALL TEXT | “Multiple gestation” |
|  | TX ALL TEXT | “Multiple pregnanc*” |
|  | TX ALL TEXT | “Fraternal twin*” |
|  | TX ALL TEXT | “Dichorionic Diamniotic” |
|  | TX ALL TEXT | “Dichorionic twin*” |
|  | TX ALL TEXT | Dichorionic |
|  | TX ALL TEXT | “Dizygotic twin*” |
|  | TX ALL TEXT | Dizygotic |
|  | TX ALL TEXT | “Nonidentical twin” |
|  | TX ALL TEXT | “Non identical twin” |
|  | TX ALL TEXT | **#1 OR #2 OR #3 OR #4 OR #5 OR #6 OR #7 OR #8 OR #9 OR #10 OR #11 OR #12 OR #13** |
|  |  | |
|  | TX ALL TEXT | “Antenatal management” |
|  | TX ALL TEXT | “Clinical practice guideline*” |
|  | TX ALL TEXT | “Clinical Guideline*” |
|  | TX ALL TEXT | Guideline* |
|  | TX ALL TEXT | Guidance |
|  | TX ALL TEXT | “Best practice” |
|  | TX ALL TEXT | Standard* |
|  | TX ALL TEXT | “Practice guideline*” |
|  | TX ALL TEXT | “Practice bulletin*” |
|  | TX ALL TEXT | Management OR Managing |
|  | TX ALL TEXT | **#15 OR #16 OR #17 OR #18 OR #19 OR #20 OR #21 OR #22 OR #23 OR #24** |
|  | TX ALL TEXT | **#14 AND #25** |
|  | Limiters | Humans  Year: 2000-2021 |

***Databases***

|  | **Database: EMBASE**  **Date of least search:** 01/07/2021 | |  |
| --- | --- | --- | --- |
|  | **Search Field** |  |  |
|  | **Concept 1 Multiple Pregnancy** | |  |
|  | ALL FIELDS | “Twin pregnanc*” |  |
|  | ALL FIELDS | “Multifetal gestation*” |  |
|  | ALL FIELDS | “Multifetal pregnanc*” |  |
|  | ALL FIELDS | “Multiple gestation” |  |
|  | ALL FIELDS | “Multiple pregnanc*” |  |
|  | ALL FIELDS | “Fraternal twin*” |  |
|  | ALL FIELDS | “Dichorionic Diamniotic” |  |
|  | ALL FIELDS | “Dichorionic twin*” |  |
|  | ALL FIELDS | Dichorionic |  |
|  | ALL FIELDS | “Dizygotic twin*” |  |
|  | ALL FIELDS | Dizygotic |  |
|  | ALL FIELDS | “Nonidentical twin” |  |
|  | ALL FIELDS | “Non identical twin” |  |
|  | ALL FIELDS | **#1 OR #2 OR #3 OR #4 OR #5 OR #6 OR #7 OR #8 OR #9 OR #10 OR #11 OR #12 OR #13** |  |
|  | **Concept 2 Clinical practice guidelines** | |  |
|  | ALL FIELDS | “Antenatal management” |  |
|  | ALL FIELDS | “Clinical practice guideline*” |  |
|  | ALL FIELDS | “Clinical Guideline*” |  |
|  | ALL FIELDS | Guideline* |  |
|  | ALL FIELDS | Guidance |  |
|  | ALL FIELDS | “Best practice” |  |
|  | ALL FIELDS | Standard* |  |
|  | ALL FIELDS | “Practice guideline*” |  |
|  | ALL FIELDS | “Practice bulletin*” |  |
|  | ALL FIELDS | Management OR Managing |  |
|  | ALL FIELDS | **#15 OR #16 OR #17 OR #18 OR #19 OR #20 OR #21 OR #22 OR #23 OR #24** |  |
|  | ALL FIELDS | **#14 AND #25** |  |
|  | Limiters | Humans  Year: 2000-2021 |  |

|  | **Database: MEDLINE**  **Date of last search:** 01/07/2021 | |  |  |  |
| --- | --- | --- | --- | --- | --- |
|  | **Search Field** | **Search Terms: Milestone Study** |  |  |  |
|  |  | |  |  |  |
|  | ALL FIELDS | “Twin pregnanc*” |  |  |  |
|  | ALL FIELDS | “Multifetal gestation*” |  |  |  |
|  | ALL FIELDS | “Multifetal pregnanc*” |  |  |  |
|  | ALL FIELDS | “Multiple gestation” |  |  |  |
|  | ALL FIELDS | “Multiple pregnanc*” |  |  |  |
|  | ALL FIELDS | “Fraternal twin*” |  |  |  |
|  | ALL FIELDS | “Dichorionic Diamniotic” |  |  |  |
|  | ALL FIELDS | “Dichorionic twin*” |  |  |  |
|  | ALL FIELDS | Dichorionic |  |  |  |
|  | ALL FIELDS | “Dizygotic twin*” |  |  |  |
|  | ALL FIELDS | Dizygotic |  |  |  |
|  | ALL FIELDS | “Nonidentical twin” |  |  |  |
|  | ALL FIELDS | “Non identical twin” |  |  |  |
|  | ALL FIELDS | **#1 OR #2 OR #3 OR #4 OR #5 OR #6 OR #7 OR #8 OR #9 OR #10 OR #11 OR #12 OR #13** |  |  |  |
|  | **Concept 2 Clinical practice guidelines** | |  | | 714 |
|  | ALL FIELDS | “Antenatal management” |  |  |  |
|  | ALL FIELDS | “Clinical practice guideline*” |  |  |  |
|  | ALL FIELDS | “Clinical Guideline*” |  |  |  |
|  | ALL FIELDS | Guideline* |  |  |  |
|  | ALL FIELDS | Guidance |  |  |  |
|  | ALL FIELDS | “Best practice” |  |  |  |
|  | ALL FIELDS | Standard* |  |  |  |
|  | ALL FIELDS | “Practice guideline*” |  |  |  |
|  | ALL FIELDS | “Practice bulletin*” |  |  |  |
|  | ALL FIELDS | Management OR Managing |  |  |  |
|  | ALL FIELDS | **#15 OR #16 OR #17 OR #18 OR #19 OR #20 OR #21 OR #22 OR #23 OR #24** |  |  |  |
|  | ALL FIELDS | **#14 AND #25** |  |  |  |
|  | Limiters | Humans  Year: 2000-2021 |  |  |  |

|  |
| --- |

|  | **Database: PubMed**  **Date of last search:** 01/07/2021 | |  |  |  |
| --- | --- | --- | --- | --- | --- |
|  | **Search Field** |  |  |  |  |
|  | **Concept 1 Multiple Pregnancy** | |  |  |  |
|  | ALL FIELDS | “Twin pregnanc*” |  |  |  |
|  | ALL FIELDS | “Multifetal gestation*” |  |  |  |
|  | ALL FIELDS | “Multifetal pregnanc*” |  |  |  |
|  | ALL FIELDS | “Multiple gestation” |  |  |  |
|  | ALL FIELDS | “Multiple pregnanc*” |  |  |  |
|  | ALL FIELDS | “Fraternal twin*” |  |  |  |
|  | ALL FIELDS | “Dichorionic Diamniotic” |  |  |  |
|  | ALL FIELDS | “Dichorionic twin*” |  |  |  |
|  | ALL FIELDS | Dichorionic |  |  |  |
|  | ALL FIELDS | “Dizygotic twin*” |  |  |  |
|  | ALL FIELDS | Dizygotic |  |  |  |
|  | ALL FIELDS | “Nonidentical twin” |  |  |  |
|  | ALL FIELDS | “Non identical twin” |  |  |  |
|  | ALL FIELDS | **#1 OR #2 OR #3 OR #4 OR #5 OR #6 OR #7 OR #8 OR #9 OR #10 OR #11 OR #12 OR #13** |  |  |  |
|  | **Concept 2 Clinical practice guidelines** | |  | | 714 |
|  | ALL FIELDS | “Antenatal management” |  |  |  |
|  | ALL FIELDS | “Clinical practice guideline*” |  |  |  |
|  | ALL FIELDS | “Clinical Guideline*” |  |  |  |
|  | ALL FIELDS | Guideline* |  |  |  |
|  | ALL FIELDS | Guidance |  |  |  |
|  | ALL FIELDS | “Best practice” |  |  |  |
|  | ALL FIELDS | Standard* |  |  |  |
|  | ALL FIELDS | “Practice guideline*” |  |  |  |
|  | ALL FIELDS | “Practice bulletin*” |  |  |  |
|  | ALL FIELDS | Management OR Managing |  |  |  |
|  | ALL FIELDS | **#15 OR #16 OR #17 OR #18 OR #19 OR #20 OR #21 OR #22 OR #23 OR #24** |  |  |  |
|  | ALL FIELDS | **#14 AND #25** |  |  |  |
|  | Limiters | Humans  Year: 2000-2021 |  |  |  |

|  | **Database: Web of Science**  **Date of last search:** 01/07/2021 | |  |  |  |
| --- | --- | --- | --- | --- | --- |
|  | **Search Field** |  |  |  |  |
|  | **Concept 1 Multiple Pregnancy** | |  |  |  |
|  | ALL FIELDS | ALL=“Twin pregnanc*” |  |  |  |
|  | ALL FIELDS | ALL=“Multifetal gestation*” |  |  |  |
|  | ALL FIELDS | ALL=“Multifetal pregnanc*” |  |  |  |
|  | ALL FIELDS | ALL=“Multiple gestation” |  |  |  |
|  | ALL FIELDS | ALL=“Multiple pregnanc*” |  |  |  |
|  | ALL FIELDS | ALL=“Fraternal twin*” |  |  |  |
|  | ALL FIELDS | ALL=“Dichorionic Diamniotic” |  |  |  |
|  | ALL FIELDS | ALL=“Dichorionic twin*” |  |  |  |
|  | ALL FIELDS | ALL=Dichorionic |  |  |  |
|  | ALL FIELDS | ALL=“Dizygotic twin*” |  |  |  |
|  | ALL FIELDS | ALL=Dizygotic |  |  |  |
|  | ALL FIELDS | ALL=“Nonidentical twin” |  |  |  |
|  | ALL FIELDS | ALL=“Non identical twin” |  |  |  |
|  | ALL FIELDS | **#1 OR #2 OR #3 OR #4 OR #5 OR #6 OR #7 OR #8 OR #9 OR #10 OR #11 OR #12 OR #13** |  |  |  |
|  | **Concept 2 Clinical practice guidelines** | |  | | 714 |
|  | ALL FIELDS | ALL=“Antenatal management” |  |  |  |
|  | ALL FIELDS | ALL=“Clinical practice guideline*” |  |  |  |
|  | ALL FIELDS | ALL=“Clinical Guideline*” |  |  |  |
|  | ALL FIELDS | ALL=Guideline* |  |  |  |
|  | ALL FIELDS | ALL=Guidance |  |  |  |
|  | ALL FIELDS | ALL=“Best practice” |  |  |  |
|  | ALL FIELDS | ALL=Standard* |  |  |  |
|  | ALL FIELDS | ALL=“Practice guideline*” |  |  |  |
|  | ALL FIELDS | ALL=“Practice bulletin*” |  |  |  |
|  | ALL FIELDS | ALL=Management OR Managing |  |  |  |
|  | ALL FIELDS | **#15 OR #16 OR #17 OR #18 OR #19 OR #20 OR #21 OR #22 OR #23 OR #24** |  |  |  |
|  | ALL= (ALL FIELDS) | **#14 AND #25** |  |  |  |
|  | Limiters | Humans  Year: 2000-2021 |  |  |  |

***Guideline repositories***

|  | **Guideline repositories**  **Date of least search:** 05/07/2021 | **Website** |
| --- | --- | --- |
| 1 | Agency for Healthcare Research and Quality (AHRQ) | <https://www.ahrq.gov/> |
| 2 | Australian Clinical Practice Guidelines | <https://www.clinicalguidelines.gov.au/> |
| 3 | Canadian Agency for Drugs and Technology in Health (CADTH) | <https://www.cadth.ca/> |
| 4 | Canadian Medical Association (CMA) Clinical Practice guidelines database | <https://joulecma.ca/cpg/homepage> |
| 5 | Emergency Care Research Institute (ECRI) | <https://www.ecri.org/> |
| 6 | Geneva Foundation for Medical Education and Research. Obstetrics and gynecology guidelines | <https://www.gfmer.ch/Guidelines/Obstetrics_gynecology_guidelinesTemplate.htm> |
| 7 | Guidelines International Network (GIN) | <https://g-i-n.net/> |
| 8 | Institute for Clinical Systems Improvement (ICSI) | <https://www.icsi.org/guidelines/> |
| 9 | Lenus: The Irish Health Repository | <https://www.lenus.ie/> |
| 10 | National Institute for Health and Care Excellence (NICE) | <https://www.nice.org.uk/guidance> |
| 11 | Scottish Intercollegiate Guidelines Network (SIGN) | <https://www.sign.ac.uk/> |
| 12 | Turning Research Into Practice (TRIP) database | <https://www.tripdatabase.com/> |
| 13 | World Health Organisation (WHO) | <https://www.who.int/publications/who-guidelines> |

***Professional bodies and Organisations***

|  | **Professional bodies and Organisations**  **Date of last search:** 07/07/2021 | **Country/Region** |
| --- | --- | --- |
| 1 | Oesterreichische Gesellschaft fur Gynakologie und Geburtshilfe (OEGGG) | [Austria](https://www.oeggg.at/) |
| 2 | Royal Belgian Society for Obstetrics and Gynaecology (RBSOG) | [Belgium](http://www.rbsog.be/) |
| 3 | Hrvatsko drustvo Ginekolozi Opstetricari (HDGO). | [Croatia](https://www.hdgo.hr/) |
| 4 | Cyprus Gynaecological and Obstetrics Society (COGS) | Cyprus |
| 5 | Czech Gynecological and Obstetrical Society (CGPS) | [Czechia](https://www.cgps.cz/) |
| 6 | Dansk Selskab for Obstetrik og Gynaekologi (DSOG) | [Denmark](https://www.dsog.dk/) |
| 7 | Eesti Naistearstide Selts (ENS) | [Estonia](https://www.ens.ee/) |
| 8 | Suomen Gynekologiyhdistys (SYG) | [Finland](https://gynekologiyhdistys.fi/) |
| 9 | Collège National des Gynécologues et Obstétriciens Français (CNGOF) | [France](http://www.cngof.fr/) |
| 10 | Deutsche Gesellschaft für Gynäkologie und Geburtshilfe (DGGG) | [Germany](https://www.dggg.de/) |
| 11 | The Hellenic Obstetrical and Gynecological Society (HSOG) | [Greece](https://www.hsoge.gr/?lang=en) |
| 12 | Magyar Nőorvos Társaság (MNT) | [Hungary](http://mnt.olo.hu/) |
| 13 | Institute of Obstetricians and Gynaecologists of the Royal College of Physicians of Ireland (RCPI) | [Ireland](https://www.rcpi.ie/faculties/obstetricians-and-gynaecologists/) |
| 14 | Società Italiana di Ginecologia e Ostetricia (SIGO) | [Italy](https://www.sigo.it/) |
| 15 | Associazione dei Ginecologi Italiani: oppedalieri, del territorio e liberi professionisti I (AOGOI) | [Italy](https://www.aogoi.it/) |
| 16 | Societa Italiana di Medicina Perinatale (SIMP) | [Italy](https://simponline.it/) |
| 17 | Latvian Association of Gynaecologists and Obstetricians | [Latvia](http://www.ginasoc.lv/) |
| 18 | Lietuvos akušerių ginekologų draugija (LAGD) | [Lithuania](https://www.lagd.lt/) |
| 19 | Société Luxembourgeoise de Gynécologie et d’Obstétrique (SLGO) | [Luxembourg](https://www.slgo.lu/) |
| 20 | Malta College of Obstetricians and Gynaecologists | Malta |
| 21 | De Nederlandse Vereniging voor Obstetrie en Gynaecologie (NVOG) | [The Netherlands](https://www.nvog.nl/) |
| 22 | Polskie Towarzystwo Ginekologów I Położników (PTGIN) | [Poland](https://www.ptgin.pl/) |
| 23 | Sociedade Portuguesa de Obstetricia e Medicina Materno-Fetal (SPOMMF) | [Portugal](https://www.spommf.pt/) |
| 24 | Normas De Orientacao Clinica (NOCS) | [Portugal](https://nocs.pt/) |
| 25 | Servico Nacional De Saude (SNS) | [Portugal](https://www.sns.gov.pt/) |
| 26 | Slovenskej gynekologicko-pôrodníckej spoločnosti (SGPS) | [Slovakia](http://sgps.sk/) |
| 27 | Slovene Association of Trainees in Gynaecologists and Obstetricians | [Slovenia](https://www.satog.org/) |
| 28 | Sociedad Española de Ginecología y Obstetricia (SEGO) | [Spain](https://sego.es/) |
| 29 | Svensk förening för Obstetrik och Gynekologi (SFOG) | [Sweden](https://www.sfog.se/start/) |
| 30 | Norsk gynekologisk forening (NFG) | [Norway](https://www.legeforeningen.no/foreningsledd/fagmed/norsk-gynekologisk-forening/) |
| 31 | Royal College of Obstetricians and Gynaecologists (RCOG) | [U.K.](https://www.rcog.org.uk/) |
| 32 | The Royal Australian and New Zealand College of Obstetricians and Gynaecologists (RANZCOG) | [Australia & New Zealand](https://ranzcog.edu.au/) |
| 33 | American Society of Reproductive Medicine (ASRM) | [U.S.](https://www.asrm.org/) |
| 34 | The American College of Obstetricians and Gynaecologists (ACOG) | [U.S.](https://www.acog.org/) |
| 35 | The Society of Obstetricians and Gynaecologists of Canada/Societé des Obstétriciens et Gynécolgues du Canada (SOGC) | [Canada](https://sogc.org/) |
| 36 | European Board and College of Obstetrics and Gynecology (EBCOG) | [Europe](https://www.ebcog.eu/start) |
| 37 | European Association of Perinatal Medicine (EAPM) | [Europe](https://www.europerinatal.eu/) |
| 38 | European Society of Human Reproduction and Embryology (ESHRE) | [Europe](https://www.eshre.eu/) |
| 39 | Society for Maternal-Fetal Medicine (SMFM) | [International](https://www.smfm.org/) |
| 40 | World Association of Perinatal Medicine (WAPM) | [International](https://www.worldperinatal.org/) |
| 41 | The International Federation of Gynaecology and Obstetrics (FIGO) | [International](https://www.figo.org/) |
| 42 | International Society for Prenatal Diagnosis (ISPD) | [International](https://www.ispdhome.org/) |
| 43 | The International Society of Ultrasound in Obstetrics and Gynecology (ISUOG) | [International](https://www.isuog.org/) |
| 44 | Nordic Federation of Societies of Obstetric and Gynaecology (NFOG) | [Scandinavia](https://nfog.org/) |

**Article Title:** Clinical practice guidelines for the antenatal management of dichorionic diamniotic twin pregnancies: a systematic review.

**Author names:**

Caroline O’Connor^1, 2*^, Emily O’Connor^1, 2, 3^, Sara Leitao^2, 3^, Shauna Barrett^4^, Keelin O’Donoghue^1, 2^

**Affiliations**

^1^ INFANT Research Centre, University College Cork, Cork, Ireland

^2^ Pregnancy Loss Research Group, Department of Obstetrics & Gynecology, University College Cork, Cork, Ireland

^3^ National Perinatal Epidemiology Center (NPEC), University College Cork, Cork, Ireland

^4^ Cork University Hospital Library, Cork University Hospital, Cork, Ireland

**Corresponding author:** *Caroline O’Connor

E-mail: carolineoconnor@ucc.ie
