# Supplementary material for: Clinical practice guidelines for the antenatal management of dichorionic diamniotic twin pregnancies: a systematic review
Source: BMC Pregnancy Childbirth. 2023 May 13;23:347. doi: 10.1186/s12884-023-05652-z (PMC10182673; doi:10.1186/s12884-023-05652-z)
Supplement: Supplementary file 2 — Additional file 2 [file 12884_2023_5652_MOESM2_ESM.docx]

| **Guideline title** | **Author** | **Year** | **Recommendation No.** | **Recommendation** | | **Strength of Recommendation** | | **Quality of evidence** | **Recommendation category specified within guideline** | **Category** |
| --- | --- | --- | --- | --- | --- | --- | --- | --- | --- | --- |
| **Multifetal Gestations: Twin, Triplet, and Higher-Order Multifetal Pregnancies (Practice Bulletin No 231).** | ACOG | 2021 | NS | The chorionicity of a multifetal pregnancy should be established as early in pregnancy as possible, and the optimal timing for determination of chorionicity by ultrasonography is in the first trimester or early second trimester | | Level B | | NS | How is chorionicity determined | Chorionicity and dating |
| **Management of Monochorionic Twin Pregnancy: Greentop Guideline No. 51** | RCOG | 2016 | NS | All women with a twin pregnancy should be offered an ultrasound examination between 11+0 weeks and 13+6 weeks of gestation (crown–rump length 45–84 mm) to assess fetal viability, gestational age and chorionicity, and to exclude major congenital malformations.  *Note : ﻿First trimester ultrasound scanning is important in multiple pregnancies as it confirms viability, determines gestational age, defines chorionicity (by denoting placental mass numbers and membrane thickness), determines the number of amniotic sacs and may identify fetal anomalies (i.e. large cystic hygroma, anencephaly).1,6 In monochorionic twins, it is also important to exclude ‘acardiac twinning’, which is associated with TRAP. At this ultrasound examination, screening for Down syndrome may also be offered to couples (see below). ﻿*  *In spontaneously conceived twins, gestational age can be determined at the first trimester scan by using the*  *crown–rump length of the larger fetus to avoid the risk of estimating it from a baby with early growth pathology.* | | B | | 2++ | Diagnosis of MC twin pregnancy | Chorionicity and dating |
| **Management of Monochorionic Twin Pregnancy: Greentop Guideline No. 51** | RCOG | 2016 | NS | Chorionicity should be determined at the time the twin pregnancy is detected by ultrasound based upon the number of placental masses, the appearance of the membrane attachment to the placenta and the membrane thickness. This scan is best performed before 14 weeks of gestation. | | D | | 4 | Diagnosis of MC twin pregnancy | Chorionicity and dating |
|  |  |  |  |  | |  | |  |  |  |
| **Management of Monochorionic Twin Pregnancy: Greentop Guideline No. 51** | RCOG | 2016 | NS | ﻿A photographic (thermal copy) record should be taken and placed in the patient’s notes documenting the ultrasound appearance of the membrane attachment to the placenta and an electronic copy stored. | | ✔ | | NS | Diagnosis of MC twin pregnancy | Chorionicity and dating |
|  |  |  |  |  | |  | |  |  |  |
| **Management of Monochorionic Twin Pregnancy: Greentop Guideline No. 51** | RCOG | 2016 | NS | ﻿If there is uncertainty about the diagnosis of chorionicity, a photographic record of the ultrasound appearance of the membrane attachment to the placenta should be retained and a second opinion should be sought. | | ✔ | | 3 | Diagnosis of MC twin pregnancy | Chorionicity and dating |
| **Management of Monochorionic Twin Pregnancy: Greentop Guideline No. 51** | RCOG | 2016 | NS | ﻿If there is still doubt in the diagnosis of chorionicity, the woman should be referred to a specialist without delay, as chorionicity is best determined before 14 weeks of gestation. | | D | | 3 | Diagnosis of MC twin pregnancy | Chorionicity and dating |
| **Management of Monochorionic Twin Pregnancy: Greentop Guideline No. 51** | RCOG | 2016 | NS | ﻿On ultrasound, the fetuses in twin pregnancies should be assigned nomenclature (i.e. upper and lower, or left and right) and this should be clearly documented in the woman’s case notes to ensure consistency throughout pregnancy. [New | | C | | 2+ | Diagnosis of MC twin pregnancy | Chorionicity and dating |
|  |  |  |  |  | |  | |  |  |  |
| **ISUOG Practice Guidelines: role of ultrasound in twin pregnancy** | ISUOG | 2016 | NS | ﻿Twin pregnancies should ideally be dated when the crown–rump length (CRL) measurement is between 45 and 84mm (i.e. 11+0 to 13+6weeks of gestation) | | D | | NS | Dating of twin pregnancy | Chorionicity and dating |
| **ISUOG Practice Guidelines: role of ultrasound in twin pregnancy** | ISUOG | 2016 | NS | ﻿  In pregnancies conceived spontaneously, the larger of the two CRLs should be used to estimate gestational age. | | C | | NS | Dating of twin pregnancy | Chorionicity and dating |
| **ISUOG Practice Guidelines: role of ultrasound in twin pregnancy** | ISUOG | 2016 | NS | ﻿Chorionicity should be determined before 13+6 weeks of gestation using the membrane thickness at the site of insertion of the amniotic membrane into the placenta, identifying the T sign or lambda sign, and the number of placental masses. An ultrasound image demonstrating the chorionicity should be kept in the records for future reference. | | D | | 3 | ﻿Determining chorionicity/amnionicity in twin pregnancy | Chorionicity and dating |
| **ISUOG Practice Guidelines: role of ultrasound in twin pregnancy** | ISUOG | 2016 | NS | ﻿ NS  If it is not possible to determine chorionicity by transabdominal or transvaginal ultrasound in the routine setting, a second opinion should be sought from a tertiary referral center. If the center is uncertain about the chorionicity, it is safer to classify the pregnancy as monochorionic | | | | 3 | ﻿Determining chorionicity/amnionicity in twin pregnancy | Chorionicity and dating |
| **ISUOG Practice Guidelines: role of ultrasound in twin pregnancy** | ISUOG | 2016 | NS | If the woman presents after 14 weeks gestation, the larger head circumference should be used. | | NS | | NS | Dating of twin pregnancy | Chorionicity and dating |
|  |  |  |  |  | |  | |  |  |  |
| **ISUOG Practice Guidelines: role of ultrasound in twin pregnancy** | ISUOG | 2016 | NS | Twin pregnancies conceived via in-vitro fertilization should be dated using the oocyte retrieval date or the embryonic age from fertilization | | | | 2+ | Dating of twin pregnancy | Chorionicity and dating |
| **ISUOG Practice Guidelines: role of ultrasound in twin pregnancy** | ISUOG | 2016 | NS | At the time at which chorionicity is determined, amnionicity (i.e. whether or not the twins share the same amniotic sac) should be determined and documented. In case of doubt, absence of the intertwin membrane  is best confirmed by transvaginal scan. Another useful finding is demonstration of cord entanglement, which is almost universal in MCMA twin pregnancy, using color and pulsed-wave Doppler ultrasound. Using pulsed-wave  Doppler, two distinct arterial waveform patterns with different heart rates are seen within the same sampling gate | | | | 4 | Dating of twin pregnancy | Chorionicity and dating |
| **ISUOG Practice Guidelines: role of ultrasound in twin pregnancy** | ISUOG | 2016 | NS | ﻿The labelling of twin fetuses should follow a reliable and consistent strategy and should be documented clearly in the woman’s notes. *NOTE ﻿Options include: labelling according to their site, either left and right, or upper and lower; or mapping in the first trimester according to the insertion of their cords relative to the placental edges and membrane insertion. In some healthcare settings, Twin A is the fetus on the right side, while Twin B is the one on the left. This information should be documented clearly in the woman’s notes in order to ensure consistent labelling during follow-up scans17. It is advisable to describe each twin using as many features as possible so as to enable others to identify them accurately; e.g. ‘Twin A (female) is on the maternal right with a posterior placenta ﻿and marginal cord insertion’.* | | Good practice point | | NS | Labelling of twin fetuses | Chorionicity and dating |
| **Ultrasound in twin pregnancies: SOGC Clinical practice guideline No. 260** | SOGC | 2011 | 1 | ﻿All patients who are suspected to have a twin pregnancy on first trimester physical examination or who are at risk (e .g ., pregnancies resulting from assisted reproductive technologies) should have first trimester ultrasound performed. | | A | | II-2 | Number of distinct placentas | Chorionicity and dating |
| **Ultrasound in twin pregnancies: SOGC Clinical practice guideline No. 260** | SOGC | 2011 | 2 | ﻿Every attempt should be made to determine and report amnionicity and chorionicity when a twin pregnancy is identified. | | A | | II-2 | Number of distinct placentas | Chorionicity and dating |
|  |  |  |  |  | |  | |  |  |  |
| **Ultrasound in twin pregnancies: SOGC Clinical practice guideline No. 260** | SOGC | 2011 | 3 | ﻿Although the accuracy in confirmation of gestational age at the first and second trimester is comparable, dating should be done with first trimester ultrasound. | | A | | II-2 | Determining gestational age in twin pregnancies | Chorionicity and dating |
|  |  |  |  |  | |  | |  |  |  |
| **Ultrasound in twin pregnancies: SOGC Clinical practice guideline No. 260** | SOGC | 2011 | 4 | ﻿Beyond the first trimester, it is suggested that a combination of parameters rather than a single parameter should be used to confirm gestational age | | C | | II-2 | Determining gestational age in twin pregnancies | Chorionicity and dating |
| **Ultrasound in twin pregnancies: SOGC Clinical practice guideline No. 260** | SOGC | 2011 | 5 | ﻿When twin pregnancy is the result of in vitro fertilization, accurate determination of gestational age should be made from the date of embryo transfer | | A | | II-1 | Determining gestational age in twin pregnancies | Chorionicity and dating |
| **Ultrasound in twin pregnancies: SOGC Clinical practice guideline No. 260** | SOGC | 2011 | 6 | ﻿**There is insufficient evidence to make a recommendation of which fetus (when discordant for size) to use to date a twin pregnancy**. However, to avoid missing a situation of early intrauterine growth restriction in one twin, most experts agree that the clinician may consider dating pregnancy using the larger fetus. | | C | | III | Determining gestational age in twin pregnancies | Chorionicity and dating |
| **Prenatal Screening for and Diagnosis of Aneuploidy in Twin Pregnancies: Joint SOGC-CCMG Clinical Practice Guideline No, 262** | SOGC | 2011 | 4 | ﻿Chorionicity has a major impact on the prenatal screening process and should be determined by ultrasound in the first trimester of all twin pregnancies. | | A | | II-2 | Prenatal screening in twins | Chorionicity and dating |
|  |  |  |  |  | |  | |  |  |  |
| **Clinical practice guideline: Management of multiple pregnancy** | HSE | 2012 | NS | Where multiple gestation is identified on ultrasound examination, chorionicity should be assigned at the earliest opportunity. This is best achieved before 14 weeks gestation, by determining the number of placental masses, the lamda or T sign and thickness of the intertwin membrane. If in doubt a second opinion should be sought. | | NS | | NS | Diagnosis of multiple pregnancy | Chorionicity and dating |
| **Clinical practice guideline: Management of multiple pregnancy** | HSE | 2012 | NS | When chorionicity is assigned, a photographic record of the ultrasound image that supports that assignment should be kept in the woman's record. | | NS | | NS | Diagnosis of multiple pregnancy | Chorionicity and dating |
| **Clinical practice guideline: Management of multiple pregnancy** | HSE | 2012 | NS | If chorionicity cannot be determined, the pregnancy should be described as of 'undetermined chorionicity' and monochorionicity should be assumed until proven otherwise. | | NS | | NS | Diagnosis of multiple pregnancy | Chorionicity and dating |
| **Twin and Triplet Pregnancy: NG137** | NICE | 2019 | 1.1.1 | ﻿Offer women with a twin or triplet pregnancy a first trimester ultrasound scan to estimate gestational age and determine chorionicity and amnionicity (ideally, these should all be performed at the same scan). | | NS | | NS | Determining gestational age | Chorionicity and dating |
| **Twin and Triplet Pregnancy: NG137** | NICE | 2019 | 1.1.2 | ﻿Estimate gestational age from the largest baby in a twin or triplet pregnancy to avoid the risk of estimating it from a baby with early growth pathology. | | NS | | NS | Determining gestational age | Chorionicity and dating |
| **Twin and Triplet Pregnancy: NG137** | NICE | 2019 | 1.1.3 | ﻿Determine chorionicity and amnionicity at the time of detecting a twin or triplet pregnancy by ultrasound using:  • the number of placental masses • the presence of amniotic membrane(s) and membrane thickness • the lambda or T-sign. | | NS | | NS | Chorionicity and amnionicity | Chorionicity and dating |
|  |  |  |  |  | |  | |  |  |  |
| **Twin and Triplet Pregnancy: NG137** | NICE | 2019 | 1.1.4 | ﻿Assign nomenclature to babies (for example, upper and lower, or left and right) in a twin or triplet pregnancy, and document this clearly in the woman's notes to ensure consistency throughout pregnancy. | | NS | | NS | Chorionicity and amnionicity | Chorionicity and dating |
| **Twin and Triplet Pregnancy: NG137** | NICE | 2019 | 1.1.5 | ﻿If a woman with a twin or triplet pregnancy presents after 14+0 weeks,  determine chorionicity and amnionicity at the earliest opportunity by ultrasound using all of the following:  • the number of placental masses • the presence of amniotic membrane(s) and membrane thickness • the lambda or T-sign • discordant fetal sex. | | NS | | NS | Chorionicity and amnionicity | Chorionicity and dating |
| **Twin and Triplet Pregnancy: NG137** | NICE | 2019 | 1.1.6 | If it is not possible to determine chorionicity or amnionicity by ultrasound at the time of detecting the twin or triplet pregnancy, seek a second opinion from a senior sonographer or refer the woman to a healthcare professional who is competent in determining chorionicity and amnionicity by ultrasound scan as soon as possible. | | NS | | NS | Chorionicity and amnionicity | Chorionicity and dating |
| **Twin and Triplet Pregnancy: NG137** | NICE | 2019 | 1.1.8 | Provide regular training so that sonographers can identify the lambda or T-sign accurately and confidently. Less experienced sonographers should have support from senior colleagues. | | NS | | NS | Chorionicity and amnionicity | Chorionicity and dating |
| **Twin and Triplet Pregnancy: NG137** | NICE | 2019 | 1.1.9 | ﻿Training should cover ultrasound scan measurements needed for women who book after 14+0 weeks and should emphasise that the risks associated with twin and triplet pregnancy are determined by chorionicity and not zygosity. | | NS | | NS | Chorionicity and amnionicity | Chorionicity and dating |
| **Twin and Triplet Pregnancy: NG137** | NICE | 2019 | 1.1.10 | ﻿Conduct regular clinical audits to evaluate the accuracy of determining chorionicity and amnionicity. | | NS | | NS | Chorionicity and amnionicity | Chorionicity and dating |
|  |  |  |  |  | |  | |  |  |  |
| **Twin pregnancy** | South Australian Perinatal Practice Guideline | 2018 | NS | Early assessment of chorionicity by ultrasound is essential in the management of multiple pregnancies. | | NS | | NS | Summary of Practice Recommendations | Chorionicity and dating |
| **Twin pregnancy** | South Australian Perinatal Practice Guideline | 2018 | NS | Early (first trimester) ultrasound is recommended to ascertain or confirm gestational age, number of fetuses and their chorionicity. | | NS | | NS | Antenatal care in pregnancy | Chorionicity and dating |
| **Twin pregnancy** | South Australian Perinatal Practice Guideline | 2018 | NS | Assign nomenclature to babies (for example, upper and lower, or left and right) and document this clearly in the woman’s notes to ensure consistency throughout pregnancy. | | NS | | NS | Antenatal care in pregnancy | Chorionicity and dating |
|  |  |  |  |  | |  | |  |  |  |
| **SMFM Special Statement: State of the science on multifetal gestations: unique considerations and importance** | The Society of Maternal-Fetal Medicine | 2020 | NS | Procedures to maintain twin designation are necessary if in utero factors are an important variable. | | NS | | NS |  | Chorionicity and dating |
| **FIGO Good clinical practice advice: management of twin pregnancy** | FIGO | 2019 | NS | Twins conceived with IVF should be dated using the date of fertilisation | | NS | | NS | Dating of the pregnancy | Chorionicity and dating |
| **FIGO Good clinical practice advice: management of twin pregnancy** | FIGO | 2019 | NS | In spontaneously conceived twins, date according to CRL for larger twin | | NS | | NS | Dating of the pregnancy | Chorionicity and dating |
| **FIGO Good clinical practice advice: management of twin pregnancy** | FIGO | 2019 | NS | Dating should take place when CRL = 45-84mm (between 11+0 and 13+6 weeks’ gestation) | | NS | | NS | Dating of the pregnancy | Chorionicity and dating |
| **FIGO Good clinical practice advice: management of twin pregnancy** | FIGO | 2019 | NS | For twins presenting >14 weeks’ dating should be by head circumference of larger twin | | NS | | NS | Dating of the pregnancy | Chorionicity and dating |
| **FIGO Good clinical practice advice: management of twin pregnancy** | FIGO | 2019 | NS | The chorionicity and amnionicity should be determined in first trimester. | | NS | | NS | Determining chorionicity and amnionicty of twin pregnancy | Chorionicity and dating |
| **FIGO Good clinical practice advice: management of twin pregnancy** | FIGO | 2019 | NS | For chronicity: by examining membrane thickness at site of insertion into placenta  1. T sign for monochorionicity, 2. lambda sign for dichorionicity | | NS | | NS | Determining chorionicity and amnionicty of twin pregnancy | Chorionicity and dating |
| **FIGO Good clinical practice advice: management of twin pregnancy** | FIGO | 2019 | NS | Twins should be labelled and described using as many features as possible during ultrasound scan. | | NS | | NS | Labelling of twins | Chorionicity and dating |
| **ACR Appropriateness Criteria: Multiple Gestation** | American College of Radiology | 2017 | Variant 1: MC or DC. First trimester US | US pregnant uterus transvaginal: usually appropriate | | 9 | | NS | Variant 1: Known or suspected multiple gestations. Monochorionic or dichorionic. 1st trimester ultrasound | Chorionicity and dating |
|  |  |  |  |  | |  | |  |  |  |
| **ACR Appropriateness Criteria: Multiple Gestation** | American College of Radiology | 2017 | Variant 1: MC or DC. First trimester US | US pregnant uterus transabdominal: usually appropriate | | 9 | | NS | Variant 1: Known or suspected multiple gestations. Monochorionic or dichorionic. 1st trimester ultrasound | Chorionicity and dating |
| **ACR Appropriateness Criteria: Multiple Gestation** | American College of Radiology | 2017 | Variant 1: MC or DC. First trimester US | US cervix transvaginal: usually not appropriate | | 2 | | NS | Variant 1: Known or suspected multiple gestations. Monochorionic or dichorionic. 1st trimester ultrasound | Chorionicity and dating |
| **ACR Appropriateness Criteria: Multiple Gestation** | American College of Radiology | 2017 | Variant 1: MC or DC. First trimester US | US duplex Doppler velocimetry: usually not appropriate | | 2 | | NS | Variant 1: Known or suspected multiple gestations. Monochorionic or dichorionic. 1st trimester ultrasound | Chorionicity and dating |
|  |  |  |  |  | |  | |  |  |  |
| **ACR Appropriateness Criteria: Multiple Gestation** | American College of Radiology | 2017 | Variant 1: MC or DC. First trimester US | US pregnant uterus biophysical profile: usually not appropriate | | 1 | | NS | Variant 1: Known or suspected multiple gestations. Monochorionic or dichorionic. 1st trimester ultrasound | Chorionicity and dating |
| **ACR Appropriateness Criteria: Multiple Gestation** | American College of Radiology | 2017 | Variant 1: MC or DC. First trimester US | US echocardiography fetal: usually not appropriate | | 1 | | NS | Variant 1: Known or suspected multiple gestations. Monochorionic or dichorionic. 1st trimester ultrasound | Chorionicity and dating |
| **ACR Appropriateness Criteria: Multiple Gestation** | American College of Radiology | 2017 | NS | Transabdominal US and transvaginal US are recommended in the first trimester when a twin pregnancy is known or suspected. Chorionicity and amnionicity are most accurately evaluated in the first trimester. | | NS | | NS | Summary of recommendations | Chorionicity and dating |
| **RANZCOG Best Practice Statement: Management of monochorionic twin pregnancy** | RANZCOG Women's Health Committee | 2021 | 1 | Chorionicity is a critical consideration in the management of twin pregnancies and should be determined by ultrasound and documented in all twin pregnancies prior to 14 weeks gestation | NS | | | Consensus-based recommendation | How is the chorionicity determined in multiple pregnancy? | Chorionicity and dating |
|  |  |  |  |  | | | |  |  |  |
| **AWMF 015-087 S2e Guideline Monitoring and Care of Twin Pregnancies** | AWMF | 2020 | 1 | The gestational age of twin pregnancies should be determined at a crown-rump length (CRL) of 45–84mm (11+0 to 13+6 weeks of gestation) | | NS | | EK | Twin pregnancy dating | Chorionicity and dating |
| **AWMF 015-087 S2e Guideline Monitoring and Care of Twin Pregnancies** | AWMF | 2020 | 2 | For spontaneously conceived twins, the larger CRL should be used for estimation of gestational age. | | C | | 2+ | Twin pregnancy dating | Chorionicity and dating |
| **AWMF 015-087 S2e Guideline Monitoring and Care of Twin Pregnancies** | AWMF | 2020 | 3 | Twins conceived after IVF should be dated based on date of egg retrieval or the age of embryo (in days) at implantation. | | C | | 2+ | Twin pregnancy dating | Chorionicity and dating |
| **AWMF 015-087 S2e Guideline Monitoring and Care of Twin Pregnancies** | AWMF | 2020 | 4 | The chorionicity should be determined before 13+6 weeks of pregnancy through: assessment of the thickness of the membrane at insertion site of the amniotic membrane into the placenta, the determination of the T or lambda symbol as well as the number of placental masses. An ultrasound image showing chorionicity should be archived in the documents for later inspection. | | A | | EK, 1+ | Determination of amnionicity and chorionicity for twin pregnancies | Chorionicity and dating |
| **AWMF 015-087 S2e Guideline Monitoring and Care of** | AWMF | 2020 | 5 | The second opinion of a specialized center should be obtained if it is not possible to determine chorionicity in a routine setting by transabdominal or transvaginal ultrasound. | | NS | | EK | Determination of amnionicity and chorionicity for twin | Chorionicity and dating |
| **Twin Pregnancies** |  |  |  |  | |  | |  | pregnancies |  |
| **AWMF 015-087 S2e Guideline Monitoring and Care of Twin Pregnancies** | AWMF | 2020 | 6 | If the determination of chorionicity is also not possible there, the pregnancy should be treated as an MC pregnancy | | NS | | EK | Determination of amnionicity and chorionicity for twin pregnancies | Chorionicity and dating |
| **AWMF 015-087 S2e Guideline Monitoring and Care of Twin Pregnancies** | AWMF | 2020 | 7 | The labelling of twin fetuses should follow a reliable and uniform strategy and be clearly documented. Several parameters should be used for this (e. g. which is to the front, the side, the position, the location of the placenta and umbilical insertion, and the sex) | | C | | EK, 2+ | Designation of twin fetuses | Chorionicity and dating |
| **Tvillinger - ﻿håndtering af graviditet og fødsel (twins- handling pregnancy and childbirth)** | Sandbjerg | 2010 | NS | The complication profile and thus the control pattern is completely different depending on whether there is speak of a dichorionic or a monochronic placentation, this is why determining chorionicity before 15 weeks gestations is recommended. | | B | | NS | Recommendations with strengths | Chorionicity and dating |
| **Tvillinger - ﻿håndtering af graviditet og fødsel (twins- handling pregnancy and childbirth)** | Sandbjerg | 2010 | NS | We recommend that you describe the following as early as possible for both TV 1 and TV 2.Placenta: describe the location of the placenta and septum as well as where the umbilical cords inserts itself. Gender: describe the sex of the fetuses (especially for different sexes).Discordant growth: describe who is the big one and who is the smaller one. Other characteristics that can distinguish between the 2 twins. TV 1: The fetus that at the neck fold scan lies most with head / body in maternal left side.TV 2: The fetus that at the neck fold scan lies most with head / body in the right of the feeder page | NS | | | NS | Nomenclature in relation to the location of the twins | Chorionicity and dating |
| **Maternity- Management of monochorionic twin pregnancy** | New South Wales Government | 2020 | 1 | Chorionicity is a critical consideration in the management of twin pregnancies and should be determined by ultrasound and documented in all twin pregnancies prior to 14 weeks gestation.  NOTE: If there is difficulty determining chorionicity and/or amnionicity, referral should be made for a maternal fetal medicine review, where possible within the Tiered Perinatal Network, ideally before 14 weeks gestation. | | NS | | NS | Chorionicity | Chorionicity and dating |
| **Ultrasound for twin and multiple pregnancies** | Toward optimized practice (TOP) | 2017 | NS | Perform a first trimester ultrasound routinely for dating in all pregnant patients (see the Toward Optimized Practice Determination of Gestational Age CPG). | | NS | | NS | PREGNANCY DATING AND FIRST TRIMESTER ULTRASOUND ASSESSMENT | Chorionicity and dating |
| **Ultrasound for twin and multiple pregnancies** | Toward optimized practice (TOP) | 2017 | NS | Determine the gestational age at the first ultrasound at or beyond seven weeks.  o The estimated date of confinement (EDC) is then assigned and should not be altered later in the pregnancy.  o For in-vitro fertilization (IVF) pregnancies, the gestational age should be determined and reported as date of conception (if available) rather than first trimester ultrasound. | | NS | | NS | PREGNANCY DATING AND FIRST TRIMESTER ULTRASOUND ASSESSMENT | Chorionicity and dating |
| **Ultrasound for twin and multiple pregnancies** | Toward optimized practice (TOP) | 2017 | NS | Suggest dating the pregnancy using the larger fetus (when discordant for size) in a twin pregnancy to avoid missing an early-onset intrauterine growth restriction in one twin. | | NS | | NS | PREGNANCY DATING AND FIRST TRIMESTER ULTRASOUND ASSESSMENT | Chorionicity and dating |
| **Ultrasound for twin and multiple pregnancies** | Toward optimized practice (TOP) | 2017 | NS | Determine definitively, and report clearly as possible, the amnionicity and chorionicity when a multiple pregnancy is identified.  o Include in report presence of lambda/twin peak sign or “T” sign, number/location of placentas, and presence of a free floating dividing membrane. | | NS | | NS | PREGNANCY DATING AND FIRST TRIMESTER ULTRASOUND ASSESSMENT | Chorionicity and dating |
| **Ultrasound for twin and multiple pregnancies** | Toward optimized practice (TOP) | 2017 | NS | Ultrasound examination between 18-20 weeks: If there has not been a prior ultrasound, determine gestational age, chorionicity, and amnionicity. | | NS | | NS | Second and third trimester studies | Chorionicity and dating |
| **Ultrasound for twin and multiple pregnancies** | Toward optimized practice (TOP) | 2017 | NS | Verify consistent determination of chorionicity and amnionicity based on prior (if available) and current imaging, and include in the report. | | NS | | NS | Second and third trimester studies | Chorionicity and dating |
| **Ultrasound for twin and multiple pregnancies** | Toward optimized practice (TOP) | 2017 | NS | If lambda or “T” signs are not evident, consider number of placental masses, assessment of thickness of dividing membrane, and fetal genders. If unable to determine chorionicity with certainty, suggest managing as per monochorionic pregnancy, or consult MFM. | | NS | | NS | Second and third trimester studies | Chorionicity and dating |
|  |  |  |  |  | |  | |  |  |  |
|  |  |  |  |  |  |  |  |  |  |  |
| **Ultrasound for twin and multiple pregnancies** | Toward optimized practice (TOP) | 2017 | NS | Report (in addition to fetal anatomical surveys):  o Identifying features of each fetus (presentation, presenting/trailing, left/right side, gender if known, larger/smaller).  o Assignment of each individual e.g., as Fetus 1/Fetus 2 early in the pregnancy and do not change assignment.  o Level of amniotic fluid in multiples at each ultrasound visit using deepest vertical pocket (DVP) measured in both gestational sacs and compare:   Oligohydramnios is defined as <2 cm.   Polyhydramnios is defined as >8 cm.   At each assessment, a free-floating dividing membrane should be visualized, and ideally the DVP should be imaged in view of the dividing membrane. | | NS | | NS | Second and third trimester studies | Chorionicity and dating |
| **SMFM Special Statement: Updated checklist for management of monochorionic twin pregnancy** | Patient safety and quality committee, SMFM, Iffath Abbasi Hoskins, C. Andrew Combs | 2020 | NS | Establish EDC, chorionicity, and amnionicity, preferably before 14 weeks of gestation | | NS | | NS | Sample checklist for management of monochorionic/diamniotic twin pregnancy | Chorionicity and dating |
| **Management of multiple pregnancy** | SIGO, AOGOI, AGUI | 2016 | NS | In all cases of twin pregnancy, a US should be done between 11+0 and 13+6 weeks of gestation to evaluate vitality, chorionicity, position and gestational age | | A | | 2 | Diagnosis of chorionicity and amnionicity | Chorionicity and dating |
|  |  |  |  |  | |  | |  |  |  |
| **Management of multiple pregnancy** | SIGO, AOGOI, AGUI | 2016 | NS | It is advisable to attach to the ultrasound report and picture documentation of the chorionicity | | NS | | NS | Diagnosis of chorionicity and amnionicity | Chorionicity and dating |
| **Management of multiple pregnancy** | SIGO, AOGOI, AGUI | 2016 | NS | In case of doubtful chorionicity, send to a tertiary center by week 14+0. | | C | | 2 | Diagnosis of chorionicity and amnionicity | Chorionicity and dating |
| **Management of multiple pregnancy** | SIGO, AOGOI, AGUI | 2016 | NS | Dating is based on the measurement of CRL of fetuses performed in the sagital position and in neutral position using the same biometric curves of singleton pregnancies. | | B | | 2 | Diagnosis of chorionicity and amnionicity | Chorionicity and dating |
|  |  |  |  |  | |  | |  |  |  |
| **Management of multiple pregnancy** | SIGO, AOGOI, AGUI | 2016 | NS | In case of pregnancy obtained by ART techniques, the gestational age is always defined by the date egg retrieval. | | A | | 2 | Diagnosis of chorionicity and amnionicity | Chorionicity and dating |
| **Management of multiple pregnancy** | SIGO, AOGOI, AGUI | 2016 | NS | The technique of "labelling" should be performed for nomenclature of fetuses defining each twin uniquely. Such a determination is based on the evaluation of the following parameters: reciprocal position of the fetuses, relationship with the cervical canal, position relative to the placenta, ultrasound features. | | B | | 3 | Diagnosis of chorionicity and amnionicity | Chorionicity and dating |
| **Management of multiple pregnancy** | SIGO, AOGOI, AGUI | 2016 | NS | After diagnosing chorionicity it must be explained to the patient that zygosity cannot always be determined, especially in pregnancies originating from ART. | | A | | 5 | Combined 1st Trim screening in multiple pregnancy | Chorionicity and dating |
| **Twin pregnancies: guidelines for clinical practice from the French College of Gynaecologists and Obstetricians (CNGOF)** | Christophe Vayssiere | 2011 | NS | Every report of an ultrasound examination of a twin pregnancy (especially during the first trimester) must include information about chorionicity (Professional Consensus) | NS | | | NS | Professional consensus | Chorionicity and dating |
| **Twin pregnancies: guidelines for clinical practice from the French College of Gynaecologists and Obstetricians (CNGOF)** | Christophe Vayssiere | 2011 | NS | It is recommended that chorionicity be diagnosed as early as possible in twin pregnancies, because the earlier the diagnosis, the more reliable it is (Professional Consensus). | NS | | | NS | Professional consensus | Chorionicity and dating |
| **Twin pregnancies: guidelines for clinical practice from the French College of Gynaecologists and Obstetricians (CNGOF)** | Christophe Vayssiere | 2011 | NS | If chorionicity cannot be diagnosed during the first trimester, the patient must be referred to a specialist ultrasonographer at an approved prenatal diagnostic center (CPDPN) (Professional Consensus) | | | NS | NS | Professional consensus | Chorionicity and dating |
|  |  |  |  |  | | |  |  |  |  |
| **Twin pregnancies: guidelines for clinical practice from the French College of Gynaecologists and Obstetricians (CNGOF)** | Christophe Vayssiere | 2011 | NS | The most relevant signs (close to 100%) are the number of gestational sacs between 7 and 10 weeks and the presence of a lambda sign between 11 and 14 weeks (Professional Consensus) | | | NS | NS | Professional consensus | Chorionicity and dating |
| **Twin pregnancies: guidelines for clinical practice from the French College of Gynaecologists and Obstetricians (CNGOF)** | Christophe Vayssiere | 2011 | NS | If chorionicity was appropriately diagnosed during the first trimester of pregnancy and the ‘‘explicit photograph of the ultrasound image allowing diagnosis of chorionicity’’ can be furnished, this diagnosis is permanent and need not be reconsidered later (Professional Consensus) | | | NS | NS | Professional consensus | Chorionicity and dating |
|  |  |  |  |  | | | |  |  |  |
| **Multiple Pregnancy** | Lithuanian Society of Obstetricians and Gynaecologists, Lithuanian Midwives Association | 2014 | 4.2 | An ultrasound should be performed for: pregnancy confirmation, number of fetuses, implantation location, number of amniotic sacs, and chorionicity | | NS | | NS | Diagnostics | Chorionicity and dating |
| **Multiple Pregnancy** | Lithuanian Society of Obstetricians and Gynaecologists, Lithuanian Midwives Association | 2014 | 4.4 | In the case of multiple pregnancies, determining chorionicity is very important in predicting complications, risks and developing a pregnancy care plan. If chorionicity cannot be determined, refer to a more experienced person for consultation. | | NS | | NS | Diagnostics | Chorionicity and dating |
|  |  |  |  |  | |  | |  |  |  |
| **Multiple Pregnancy** | Lithuanian Society of Obstetricians and Gynaecologists, Lithuanian Midwives Association | 2014 | 4.5 | The optimal time to detect chorionicity is 10 to 13 weeks of gestation. | | NS | | NS | Diagnostics | Chorionicity and dating |
| **Multiple Pregnancy** | Lithuanian Society of Obstetricians and Gynaecologists, Lithuanian Midwives Association | 2014 | 4.6 | During the first pregnancy, ultrasound is recommended at weeks 11 to 13, when the CRL measures 45 to 84 mm. During this one study it is possible to determine the gestational age, chorionicity and measure nuchal translucency (checking for Down syndrome). | | NS | | NS | Diagnostics | Chorionicity and dating |
|  |  |  |  |  | |  | |  |  |  |
| **Multiple Pregnancy** | Lithuanian Society of Obstetricians and Gynaecologists, Lithuanian Midwives Association | 2014 | 4.7 | Pregnancy is dated according to the larger CRL dimension to reduce the risk of that the time of pregnancy will be erroneously determined by a fetus with early growth failure. | | NS | | NS | Diagnostics | Chorionicity and dating |
| **Multiple Pregnancy** | Lithuanian Society of Obstetricians and Gynaecologists, Lithuanian Midwives Association | 2014 | 4.8 | The most commonly used terms to describe the position of the fetus are: upper and lower, left and right. | | NS | | NS | Diagnostics | Chorionicity and dating |
|  |  |  |  |  | |  | |  |  |  |
| **Multiple Pregnancy** | Lithuanian Society of Obstetricians and Gynaecologists, Lithuanian Midwives Association | 2014 | 4.9 | If the pregnant woman visits after 14 weeks, an ultrasound should be performed as soon as possible. In this study currently set:● number of placenta;● λ and T features;● cover partition thickness;● sex of the fetus;● The deepest water pockets of all fetuses | | NS | | NS | Diagnostics | Chorionicity and dating |
| **Multiple Pregnancy** | Lithuanian Society of Obstetricians and Gynaecologists, Lithuanian Midwives Association | 2014 | 4.10 | Do not use 3D ultrasound to determine chorionicity | | NS | | NS | Diagnostics | Chorionicity and dating |
|  |  |  |  |  | |  | |  |  |  |
| **Multiple Pregnancy** | Lithuanian Society of Obstetricians and Gynaecologists, Lithuanian Midwives Association | 2014 | 4.11 | If chorionicity cannot be determined, the pregnancy should be monitored as monochorionic until it can be confirmed otherwise | | NS | | NS | Diagnostics | Chorionicity and dating |
| **Role of ultrasonography in the management of twin gestation** | International Federation of Gynecology and Obstetrics (FIGO) | 2018 | NS | Chorionicity is best determined by ultrasonography before 14 weeks of gestation and has an accuracy of 99%. | | NS | | NS | Chorionicity and Amnionicity | Chorionicity and dating |
| **Role of ultrasonography in the management of twin gestation** | International Federation of Gynecology and Obstetrics (FIGO) | 2018 | NS | If an ultrasonography examination has not been performed before 14 weeks of gestation to determine chorionicity, ultrasonography determination of chorionicity should be attempted at the time of presentation. | | NS | | NS | Chorionicity and Amnionicity | Chorionicity and dating |
|  |  |  |  |  | |  | |  |  |  |
| **Role of ultrasonography in the management of twin gestation** | International Federation of Gynecology and Obstetrics (FIGO) | 2018 | NS | If the chorionicity of the pregnancy cannot be determined, the pregnancy should be classified as “presumed monochorionic,” and monitored and managed as such for the remainder of the gestation | | NS | | NS | Chorionicity and Amnionicity | Chorionicity and dating |
| **Role of ultrasonography in the management of twin gestation** | International Federation of Gynecology and Obstetrics (FIGO) | 2018 | NS | As in singleton pregnancies, if the crown rump length (CRL) is consistent with a sure last menstrual period (LMP), the LMP should be used for pregnancy dating | | NS | | NS | Chorionicity and Amnionicity | Chorionicity and dating |
| **Role of ultrasonography in the management of twin gestation** | International Federation of Gynecology and Obstetrics (FIGO) | 2018 | NS | The larger CRL should be used for dating if the CRLs of the two fetuses are discordant. | | NS | | NS | Chorionicity and Amnionicity | Chorionicity and dating |
|  |  |  |  |  | |  | |  |  |  |
| **Role of ultrasonography in the management of twin gestation** | International Federation of Gynecology and Obstetrics (FIGO) | 2018 | NS | If the first ultrasonography exam is not performed until after 14 weeks, biometric parameters should be used to determine gestational age rather than CRL. Again, the larger head circumference should be used to determine pregnancy dating if the biometric parameters of the two fetuses are discordant. | | NS | | NS | Chorionicity and Amnionicity | Chorionicity and dating |
| **Role of ultrasonography in the management of twin gestation** | International Federation of Gynecology and Obstetrics (FIGO) | 2018 | NS | Dichorionic/diamniotic gestation and pregnancy dating are ideally determined in the first trimester. | | NS | | NS | Diamniotic/dichorionic twins | Chorionicity and dating |
| **Role of ultrasonography in the management of twin gestation** | International Federation of Gynecology and Obstetrics (FIGO) | 2018 | Table 4 | First trimester: Document chorionicity, document viability, establish dating based on larger CRL, offer first-trimester screening | | NS | | NS | General guidelines for ultrasonography imaging in twin pregnancies TABLE 4 | Chorionicity and dating |

**Article Title:** Clinical practice guidelines for the antenatal management of dichorionic diamniotic twin pregnancies: a systematic review.

**Author names:**

Caroline O’Connor^1, 2*^, Emily O’Connor^1, 2, 3^, Sara Leitao^2, 3^, Shauna Barrett^4^, Keelin O’Donoghue^1, 2^

**Affiliations**

^1^ INFANT Research Centre, University College Cork, Cork, Ireland

^2^ Pregnancy Loss Research Group, Department of Obstetrics & Gynecology, University College Cork, Cork, Ireland

^3^ National Perinatal Epidemiology Center (NPEC), University College Cork, Cork, Ireland

^4^ Cork University Hospital Library, Cork University Hospital, Cork, Ireland

**Corresponding author:** *Caroline O’Connor

E-mail: carolineoconnor@ucc.ie
