# Supplementary material for: Clinical practice guidelines for the antenatal management of dichorionic diamniotic twin pregnancies: a systematic review
Source: BMC Pregnancy Childbirth. 2023 May 13;23:347. doi: 10.1186/s12884-023-05652-z (PMC10182673; doi:10.1186/s12884-023-05652-z)
Supplement: Supplementary file 3 — Additional file 3 [file 12884_2023_5652_MOESM3_ESM.docx]

| **Guideline title** | **Author** | **Year** | **Recommendation No.** | **Recommendation** | **Strength of Recommendation** | **Quality of evidence** | **Recommendation category specified within guideline** | **Category** | **Subcategory** |
| --- | --- | --- | --- | --- | --- | --- | --- | --- | --- |
| **Multifetal Gestations: Twin, Triplet, and Higher-Order Multifetal Pregnancies (Practice Bulletin No 231).** | ACOG | 2021 | NS | For women with dichorionic twin gestations, there are no evidence-based recommendations on the frequency of fetal growth scans after 20 weeks of gestation; however, it seems reasonable that serial ultra-sonographic surveillance be performed every 4 weeks in the absence of evidence of fetal growth restriction or other pregnancy complications | NS | NS | None | Fetal growth | Scanning |
| **Multifetal Gestations: Twin, Triplet, and Higher-Order Multifetal Pregnancies (Practice Bulletin No 231).** | ACOG | 2021 | NS | The optimal gestational age for initiation of surveillance in pregnant individuals with uncomplicated dichorionic twins is not known. However, for patients with uncomplicated dichorionic twin pregnancies, weekly antenatal fetal surveillance may be considered at 36 0/7 weeks of gestation. | Level C | NS | None | Fetal growth | Scanning |
| **Multifetal Gestations: Twin, Triplet, and Higher-Order Multifetal Pregnancies (Practice Bulletin No 231).** | ACOG | 2021 | NS | Discordant fetal growth in women with multifetal gestations is most commonly defined as a 20% difference in estimated fetal weight between larger and smaller fetus | NS | NS | None | Fetal growth | Scanning |
| **Multifetal Gestations: Twin, Triplet, and Higher-Order Multifetal Pregnancies (Practice Bulletin No 231).** | ACOG | 2021 | NS | For patients with a dichorionic twin pregnancy complicated by maternal or fetal disorders such as fetal growth restriction, antenatal fetal surveillance should be individualized and may be considered upon diagnosis, or at a gestational age after which delivery would be considered for abnormal testing. | Level C | NS | None | Fetal growth | Scanning |
| **ISUOG Practice Guidelines: role of ultrasound in twin pregnancy** | ISUOG | 2016 | NS | ﻿Women with an uncomplicated dichorionic twin pregnancy should have a first-trimester scan, a detailed second-trimester scan, and scans every 4 weeks there- after. Complicated dichorionic twins should be scanned more frequently, depending on the condition and its severity. *NOTE: ﻿In uncomplicated dichorionic twin pregnancy, ultrasound imaging should be performed in the first trimester, again at around 20 weeks’ gestation (second-trimester ﻿anomaly scan), and every 4 weeks thereafter (unless a complication is detected which might require more frequent scans).* | Good practice point | NS | ﻿Routine monitoring of twin pregnancy with ultrasound | Fetal growth | Scanning |
| **ISUOG Practice Guidelines: role of ultrasound in twin pregnancy** | ISUOG | 2016 | NS | The management of twin pregnancy with CRL discordance ≥ 10% or of NT discordance ≥20% should be discussed with a fetal medicine expert. | B |  | Implications for discordance in NT or CRL in the first trimester. | Fetal growth | Management |
| **ISUOG Practice Guidelines: role of ultrasound in twin pregnancy** | ISUOG | 2016 | NS | Selective fetal growth restriction (sFGR), conventionally, is defined as a condition in which one fetus has EFW<10th centile and the intertwin EFW discordance is>25%. | Good practice point |  | ﻿Screening, diagnosis and management of fetal growth restriction (FGR). | Fetal growth | Scanning |
| **ISUOG Practice Guidelines: role of ultrasound in twin pregnancy** | ISUOG | 2016 | NS | EFW discordance is calculated by the following formula: ((weight of larger twin – weight of smaller twin)×100)/weight of larger twin |  | 2++ | ﻿Screening, diagnosis and management of fetal growth restriction (FGR). | Fetal growth | Scanning |
| **ISUOG Practice Guidelines: role of ultrasound in twin pregnancy** | ISUOG | 2016 | NS | Once a diagnosis (of growth discordance) has been made, a cause should be sought. This search should include a detailed anomaly scan and screening for viral infections (cytomegalovirus, rubella and toxoplasmosis). Amniocentesis may also be required to exclude chromosomal abnormalities as a cause of FGR. |  |  | ﻿Screening, diagnosis and management of fetal growth restriction (FGR). | Fetal growth | Management |
| **ISUOG Practice Guidelines: role of ultrasound in twin pregnancy** | ISUOG | 2016 | NS | ﻿A discordance cut-off of 20% seems acceptable to distinguish pregnancies at increased risk of adverse outcome. | B |  | ﻿Screening, diagnosis and management of fetal growth restriction (FGR). | Fetal growth | Scanning |
| **ISUOG Practice Guidelines: role of ultrasound in twin pregnancy** | ISUOG | 2016 | NS | ﻿A combination of head, abdomen and femur measurements performs best in calculating EFW | B |  | ﻿Screening, diagnosis and management of fetal growth restriction (FGR). | Fetal growth | Scanning |
| **ISUOG Practice Guidelines: role of ultrasound in twin pregnancy** | ISUOG | 2016 | NS | ﻿If intertwin discordance is ≥25%, a referral should be made to a tertiary fetal medicine center. | Good practice point | 2++ | ﻿Screening, diagnosis and management of fetal growth restriction (FGR). | Fetal growth | Management |
| **ISUOG Practice Guidelines: role of ultrasound in twin pregnancy** | ISUOG | 2016 | NS | EFW charts that include a combination of head, abdomen and femur measurements perform best in both singleton and twin pregnancy. Currently, the charts used to monitor fetal growth in twin pregnancy are the same as those used for singletons. |  | 2++ | ﻿Screening, diagnosis and management of fetal growth restriction (FGR). | Fetal growth | Fetal growth charts |
| **ISUOG Practice Guidelines: role of ultrasound in twin pregnancy** | ISUOG | 2016 | NS | ﻿In dichorionic pregnancies, sFGR should be followed as in growth-restricted singletons . | Good practice point | 2- | ﻿Screening, diagnosis and management of fetal growth restriction (FGR). | Fetal growth | Fetal growth charts |
| **ISUOG Practice Guidelines: role of ultrasound in twin pregnancy** | ISUOG | 2016 | NS | ﻿In dichorionic twin pregnancy complicated by sFGR, fetal Doppler should be assessed approximately every 2 weeks, depending on the severity. | Good practice point |  | ﻿Screening, diagnosis and management of fetal growth restriction (FGR). | Fetal growth | Scanning |
| **Ultrasound in twin pregnancies: SOGC Clinical practice guideline No. 260** | SOGC | 2011 | Summary statement 3 | ﻿Singleton growth curves currently provide the best predictors of adverse outcome in twins and may be used for evaluating growth abnormalities. | NS | III | Assessment of fetal growth | Fetal growth | Fetal growth charts |
| **Ultrasound in twin pregnancies: SOGC Clinical practice guideline No. 260** | SOGC | 2011 | Summary statement 4 | ﻿It is suggested that growth discordance be defined using either a difference (20 mm) in absolute measurement in abdominal circumference or a difference of 20% in ultrasound derived estimated fetal weight. | NS | II-2 | Assessment of fetal growth | Fetal growth | Scanning |
| **Ultrasound in twin pregnancies: SOGC Clinical practice guideline No. 260** | SOGC | 2011 | 10 | ﻿Increased fetal surveillance should be considered when there is either growth restriction diagnosed in one twin or significant growth discordance. | A | II-2 | Fetal surveillance | Fetal growth | Scanning |
| **Ultrasound in twin pregnancies: SOGC Clinical practice guideline No. 260** | SOGC | 2011 | Summary statement 5 | ﻿Although there is insufficient evidence to recommend a specific schedule for ultrasound assessment of twin gestation, most experts recommend serial ultrasound assessment every 3 to 4 weeks, starting from the anatomy scan (18 to 22 weeks) for dichorionic pregnancies. | NS | II-1 | Fetal surveillance | Fetal growth | Scanning |
| **Ultrasound in twin pregnancies: SOGC Clinical practice guideline No. 260** | SOGC | 2011 | 11 | ﻿Umbilical artery doppler should not be routinely offered in uncomplicated twin pregnancies . | E | I | Use of umbilical artery doppler velocimetry in twins | Fetal growth | Scanning |
| **Ultrasound in twin pregnancies: SOGC Clinical practice guideline No. 260** | SOGC | 2011 | 12 | ﻿For defining oligohydramnios and polyhydramnios, the ultrasonographer should use the deepest vertical pocket in either sac: oligohydramnios when < 2 cm and polyhydramnios when > 8 cm . | B | II-2 | Assessment of amniotic fluid | Fetal growth | Scanning |
| **Ultrasound in twin pregnancies: SOGC Clinical practice guideline No. 260** | SOGC | 2011 | Summary statement 7 | ﻿Although many methods of evaluating the level of amniotic fluid in twins (deepest vertical pocket, single pocket, amniotic fluid index) have been described, there is not enough evidence to suggest that one method is more predictive than the others of adverse pregnancy outcome. | NS | II-3 | Assessment of amniotic fluid | Fetal growth | Scanning |
| **Clinical practice guideline: Management of multiple pregnancy** | HSE | 2012 | NS | In the case of uncomplicated dichorionic twin gestation, serial growth evaluation should be performed at least every 4 weeks from 24 weeks gestation until 36 weeks' gestation and weekly thereafter. | NS | NS | Ultrasound surveillence | Fetal growth | Scanning |
| **Twin and Triplet Pregnancy: NG137** | NICE | 2019 | 1.4.10 | ﻿Consider scheduling ultrasound scans in twin and triplet pregnancies at a slightly later gestational age than in singleton pregnancies and be aware that the scans will take longer to perform. | NS | NS | Screening for structural abnormalities | Fetal growth | Scanning |
| **Twin and Triplet Pregnancy: NG137** | NICE | 2019 | 1.4.12 | ﻿Allow 30 minutes for growth scans in twin and triplet pregnancies. | NS | NS | Screening for structural abnormalities | Fetal growth | Scanning |
| **Twin and Triplet Pregnancy: NG137** | NICE | 2019 | 1.4.16 | ﻿Do not offer women with a twin or triplet pregnancy screening for fetal growth restriction or feto-fetal transfusion syndrome in the first trimester. | NS | NS | ﻿Screening for fetal growth restriction and feto-fetal transfusion syndrome in the first trimester | Fetal growth | Scanning |
| **Twin and Triplet Pregnancy: NG137** | NICE | 2019 | 1.4.18 | ﻿At each ultrasound scan from 24 weeks, offer women with a dichorionic twin or trichorionic triplet pregnancy diagnostic monitoring for fetal weight discordance using 2 or more biometric parameters and amniotic fluid levels. To assess amniotic fluid levels, measure the deepest vertical pocket (DVP) on either side of the amniotic membrane. | NS | NS | ﻿Diagnostic monitoring for fetal growth restriction in dichorionic twin and trichorionic triplet pregnancies | Fetal growth | Scanning |
| **Twin and Triplet Pregnancy: NG137** | NICE | 2019 | 1.4.19 | ﻿Continue monitoring for fetal weight discordance at intervals that do not exceed: • 28 days for women with a dichorionic twin pregnancy • 14 days for women with a trichorionic triplet pregnancy. | NS | NS | ﻿Diagnostic monitoring for fetal growth restriction in dichorionic twin and trichorionic triplet pregnancies | Fetal growth | Scanning |
| **Twin and Triplet Pregnancy: NG137** | NICE | 2019 | 1.4.20 | ﻿Calculate and document estimated fetal weight (EFW) discordance for dichorionic twins using the formula below [2019]: (EFW larger fetus − EFW smaller fetus) ÷ EFW larger fetus | NS | NS | ﻿Diagnostic monitoring for fetal growth restriction in dichorionic twin and trichorionic triplet pregnancies | Fetal growth | Scanning |
| **Twin and Triplet Pregnancy: NG137** | NICE | 2019 | 1.4.22 | ﻿Increase diagnostic monitoring in the second and third trimesters to at least weekly, and include doppler assessment of the umbilical artery flow for each baby, if: • there is an EFW discordance of 20% or more and/or • the EFW of any of the babies is below the 10th centile for gestational age. | NS | NS | ﻿Diagnostic monitoring for fetal growth restriction in dichorionic twin and trichorionic triplet pregnancies | Fetal growth | Scanning |
| **Twin and Triplet Pregnancy: NG137** | NICE | 2019 | 1.4.23 | ﻿Refer women with a dichorionic twin or trichorionic triplet pregnancy to a tertiary level fetal medicine centre if there is an EFW discordance of 25% or more and the EFW of any of the babies is below the 10th centile for gestational age because this is a clinically important indicator of selective fetal growth restriction. | NS | NS | ﻿Diagnostic monitoring for fetal growth restriction in dichorionic twin and trichorionic triplet pregnancies | Fetal growth | Management |
| **Twin pregnancy** | South Australian Perinatal Practice Guideline | 2018 | NS | Twin pregnancies require additional ultrasound monitoring. The frequency of this is determined by chorionicity and fetal growth patterns. | NS | NS | Summary of Practice Recommendations | Fetal growth | Scanning |
| **Twin pregnancy** | South Australian Perinatal Practice Guideline | 2018 | NS | Discordant fetal growth requires further investigation and/or referral to specialist services. | NS | NS | Summary of Practice Recommendations | Fetal growth | Management |
| **Twin pregnancy** | South Australian Perinatal Practice Guideline | 2018 | NS | In dichorionic twin pregnancy, ultrasound is recommended every 3-4 weeks from 24 weeks onwards to detect discordance in fetal size, amniotic fluid volume and umbilical artery Dopplers. Umbilical artery flow velocity studies are indicated especially in monochorionic pregnancies and when there are signs of discordancy. | NS | NS | Subsequent care in pregnancy | Fetal growth | Scanning |
| **Twin pregnancy** | South Australian Perinatal Practice Guideline | 2018 | NS | Twins growing to their full potential should follow the singleton growth curve until 32 – 35 weeks. | NS | NS | Subsequent care in pregnancy | Fetal growth | Fetal growth charts |
| **Twin pregnancy** | South Australian Perinatal Practice Guideline | 2018 | NS | Consider further investigations or delivery depending on gestation if growth is below the 10th percentile for the singleton curve or showing significant disparity between twin measurements. | NS | NS | Subsequent care in pregnancy | Fetal growth | Scanning |
| **Twin pregnancy** | South Australian Perinatal Practice Guideline | 2018 | NS | Twin pregnancies require specialist antenatal care and referral to hospitals with adequate facilities when complications such as inadequate or discordant fetal growth occur | NS | NS | Subsequent care in pregnancy | Fetal growth | Management |
| **FIGO Good clinical practice advice: management of twin pregnancy** | FIGO | 2019 | NS | Following 1st trim scans, subsequent scans should be done around weeks 20 (including anomaly scan), 24, 28, 32 and 36. | NS | NS | Timing, frequency and content of ultrasound assessment | Fetal growth | Scanning |
| **FIGO Good clinical practice advice: management of twin pregnancy** | FIGO | 2019 | NS | At each scan, measure: biometry, AFI, EFW. | NS | NS | Timing, frequency and content of ultrasound assessment | Fetal growth | Scanning |
| **FIGO Good clinical practice advice: management of twin pregnancy** | FIGO | 2019 | NS | If weight discordance, do umbilical artery Doppler assessment. | NS | NS | Timing, frequency and content of ultrasound assessment | Fetal growth | Scanning |
| **FIGO Good clinical practice advice: management of twin pregnancy** | FIGO | 2019 | NS | Estimated fetal weight discordance is calculated with this formula: (weight of larger twin - weight of smaller twin) x100 / weight of larger twin. | NS | NS | Screening, diagnosis and management of fetal growth restriction | Fetal growth | Scanning |
| **FIGO Good clinical practice advice: management of twin pregnancy** | FIGO | 2019 | NS | When selective FGR is diagnosed, an underlying cause should be sought. | NS | NS | Screening, diagnosis and management of fetal growth restriction | Fetal growth | Management |
| **FIGO Good clinical practice advice: management of twin pregnancy** | FIGO | 2019 | NS | Use of twin-specific growth charts may reduce unnecessary medical intervention. | NS | NS | Screening, diagnosis and management of fetal growth restriction | Fetal growth | Fetal growth charts |
| **FIGO Good clinical practice advice: management of twin pregnancy** | FIGO | 2019 | NS | If weight discordance >25%, refer to a tertiary unit for further management. | NS | NS | Screening, diagnosis and management of fetal growth restriction | Fetal growth | Management |
| **ACR Appropriateness Criteria: Multiple Gestation** | American College of Radiology | 2017 | Variant 1: MC or DC. First trimester US | US pregnant uterus transabdominal: usually appropriate | 9 |  | Variant 2: multiple gestations. DC. 2nd trimester US. Anatomy scan. | Fetal growth | Scanning |
| **ACR Appropriateness Criteria: Multiple Gestation** | American College of Radiology | 2017 | Variant 1: MC or DC. First trimester US | US cervix transvaginal: usually appropriate | 7 |  | Variant 2: multiple gestations. DC. 2nd trimester US. Anatomy scan. | Fetal growth | Scanning |
| **ACR Appropriateness Criteria: Multiple Gestation** | American College of Radiology | 2017 | Variant 1: MC or DC. First trimester US | US echocardiography fetal: may be appropriate | 4 |  | Variant 2: multiple gestations. DC. 2nd trimester US. Anatomy scan. | Fetal growth | Scanning |
| **ACR Appropriateness Criteria: Multiple Gestation** | American College of Radiology | 2017 | Variant 1: MC or DC. First trimester US | US duplex Doppler velocimetry: usually not appropriate | 3 |  | Variant 2: multiple gestations. DC. 2nd trimester US. Anatomy scan. | Fetal growth | Scanning |
| **ACR Appropriateness Criteria: Multiple Gestation** | American College of Radiology | 2017 | Variant 1: MC or DC. First trimester US | US pregnant uterus biophysical profile: usually not appropriate | 2 |  | Variant 2: multiple gestations. DC. 2nd trimester US. Anatomy scan. | Fetal growth | Scanning |
| **ACR Appropriateness Criteria: Multiple Gestation** | American College of Radiology | 2017 | Variant 1: MC or DC. First trimester US | US pregnant uterus transvaginal: usually not appropriate | 2 |  | Variant 2: multiple gestations. DC. 2nd trimester US. Anatomy scan. | Fetal growth | Scanning |
| **ACR Appropriateness Criteria: Multiple Gestation** | American College of Radiology | 2017 | Variant 4. DC. Growth and antepartum surveillance | US pregnant uterus transabdominal: usually appropriate | 9 |  | Variant 4: multiple gestations. DC. Growth and antepartum surveillance | Fetal growth | Scanning |
| **ACR Appropriateness Criteria: Multiple Gestation** | American College of Radiology | 2017 | Variant 4. DC. Growth and antepartum surveillance | US pregnant uterus biophysical profile: usually appropriate | 8 |  | Variant 4: multiple gestations. DC. Growth and antepartum surveillance | Fetal growth | Scanning |
| **ACR Appropriateness Criteria: Multiple Gestation** | American College of Radiology | 2017 | Variant 4. DC. Growth and antepartum surveillance | US cervix transvaginal: may be appropriate | 5 |  | Variant 4: multiple gestations. DC. Growth and antepartum surveillance | Fetal growth | Scanning |
| **ACR Appropriateness Criteria: Multiple Gestation** | American College of Radiology | 2017 | Variant 4. DC. Growth and antepartum surveillance | US duplex Doppler velocimetry: may be appropriate | 5 |  | Variant 4: multiple gestations. DC. Growth and antepartum surveillance | Fetal growth | Scanning |
| **ACR Appropriateness Criteria: Multiple Gestation** | American College of Radiology | 2017 | Variant 4. DC. Growth and antepartum surveillance | US echocardiography fetal: usually not appropriate | 3 |  | Variant 4: multiple gestations. DC. Growth and antepartum surveillance | Fetal growth | Scanning |
| **ACR Appropriateness Criteria: Multiple Gestation** | American College of Radiology | 2017 | Variant 4. DC. Growth and antepartum surveillance | US pregnant uterus transvaginal: usually not appropriate | 2 |  | Variant 4: multiple gestations. DC. Growth and antepartum surveillance | Fetal growth | Scanning |
| **ACR Appropriateness Criteria: Multiple Gestation** | American College of Radiology | 2017 | Variant 6: Known twin discordance> DC or MC | US pregnant uterus transabdominal: usually appropriate | 9 |  | Variant 6: Multiple gestations. Known twin discordance. MC or DC. | Fetal growth | Scanning |
| **ACR Appropriateness Criteria: Multiple Gestation** | American College of Radiology | 2017 | Variant 6: Known twin discordance> DC or MC | US duplex Doppler velocimetry: usually appropriate | 9 |  | Variant 6: Multiple gestations. Known twin discordance. MC or DC. | Fetal growth | Scanning |
| **ACR Appropriateness Criteria: Multiple Gestation** | American College of Radiology | 2017 | Variant 6: Known twin discordance> DC or MC | US pregnant uterus biophysical profile: usually appropriate | 8 |  | Variant 6: Multiple gestations. Known twin discordance. MC or DC. | Fetal growth | Scanning |
| **ACR Appropriateness Criteria: Multiple Gestation** | American College of Radiology | 2017 | Variant 6: Known twin discordance> DC or MC | US cervix transvaginal: may be appropriate | 5 |  | Variant 6: Multiple gestations. Known twin discordance. MC or DC. | Fetal growth | Scanning |
| **ACR Appropriateness Criteria: Multiple Gestation** | American College of Radiology | 2017 | Variant 6: Known twin discordance> DC or MC | US echocardiography fetal: may be appropriate | 5 |  | Variant 6: Multiple gestations. Known twin discordance. MC or DC. | Fetal growth | Scanning |
| **ACR Appropriateness Criteria: Multiple Gestation** | American College of Radiology | 2017 | Variant 6: Known twin discordance> DC or MC | US pregnant uterus transvaginal: usually not appropriate | 3 |  | Variant 6: Multiple gestations. Known twin discordance. MC or DC. | Fetal growth | Scanning |
| **ACR Appropriateness Criteria: Multiple Gestation** | American College of Radiology | 2017 | NS | Transabdominal US is recommended for dichorionic twins when evaluating fetal anatomy. Transvaginal US of the cervix may help triage patients into higher risk group for preterm delivery. Fetal echocardiography may be useful in some instances, such as when twins are conceived through in vitro fertilization. | NS |  | Summary of recommendations | Fetal growth | Scanning |
| **ACR Appropriateness Criteria: Multiple Gestation** | American College of Radiology | 2017 | NS | Transabdominal US is recommended for growth and antepartum surveillance for dichorionic twins with duplex Doppler velocimetry used in cases of growth discrepancy. | NS |  | Summary of recommendations | Fetal growth | Scanning |
| **ACR Appropriateness Criteria: Multiple Gestation** | American College of Radiology | 2017 | NS | Transabdominal US, duplex Doppler velocimetry, and BPP monitoring are recommended for follow-up of known twin discrepancy. Fetal echocardiography is helpful in monochorionic-monoamniotic twins. | NS |  | Summary of recommendations | Fetal growth | Scanning |
| **AWMF 015-087 S2e Guideline Monitoring and Care of Twin Pregnancies** | AWMF | 2020 | 9 | In any general ultrasound examination of twins, the following parameters should be assessed in both twins with 20 weeks or more of gestational age: – biometry, estimated weight and difference of estimated fetal weights (%), amniotic fluid volume (deepest vertical pocket, DVP) and umbilical artery Doppler. – An estimated weight difference ≥ 25% indicates selective fetal growth restriction, sFGR, for which referral to a specialized center is indicated | C | 2+ | Routine monitoring of twin pregnancies by US | Fetal growth | Scanning |
| **AWMF 015-087 S2e Guideline Monitoring and Care of Twin Pregnancies** | AWMF | 2020 | 10 | Uncomplicated DC twin pregnancies should receive first-trimester screening, a detailed second-trimester malformation ultrasound (organ screening), and subsequently serial growth measurements and Doppler ultrasound examinations every 4 weeks. Complicated DC twins should be examined more often, depending on the circumstances and their severity | NS | EK | Routine monitoring of twin pregnancies by US | Fetal growth | Scanning |
| **AWMF 015-087 S2e Guideline Monitoring and Care of Twin Pregnancies** | AWMF | 2020 | 20 | The management of twin pregnancies with an NT discordance >20% or an SSL discordance >10% between 11-13+6 weeks of pregnancy should be discussed with an expert in fetal medicine. | B | 2+, 2++ | Implications of discordances in NT or SSL in 1st trimester | Fetal growth | Management |
| **AWMF 015-087 S2e Guideline Monitoring and Care of Twin Pregnancies** | AWMF | 2020 | 28 | A single estimated weight < 3rd percentile in a twin, independent of chorionicity defines sFGR. For DC twins, at least 2 of the following 3 parameters must be met (fetal weight of one fetus < 10th percentile, EFW differential ≥ 25 %, umbilical artery PI of the smaller fetus > 95th percentile) | D | 4th | Intrauterine growth restriction | Fetal growth | Scanning |
| **AWMF 015-087 S2e Guideline Monitoring and Care of Twin Pregnancies** | AWMF | 2020 | 30 | The estimated weight discordance should be calculated using the following formula: (weight of the larger twin - weight of the smaller twin) x 100/weight of the larger twin. | NS | EK | Intrauterine growth restriction | Fetal growth | Scanning |
| **AWMF 015-087 S2e Guideline Monitoring and Care of Twin Pregnancies** | AWMF | 2020 | 31 | A search for the underlying causes of sFGR should include the following examinations: ultrasound scan, Doppler sonography, genetic family history and testing, infection screening | NS | EK | Intrauterine growth restriction | Fetal growth | Management |
| **AWMF 015-087 S2e Guideline Monitoring and Care of Twin Pregnancies** | AWMF | 2020 | 33 | A combination of head, abdomen and femur measurements is best used to estimate fetal weight | B | 2++ | Intrauterine growth restriction | Fetal growth | Scanning |
| **AWMF 015-087 S2e Guideline Monitoring and Care of Twin Pregnancies** | AWMF | 2020 | 34 | If the difference in the estimated fetal weights is ≥ 25 %, the patient should be referred to a Level 1 Perinatal Medicine Center | NS | EK | Intrauterine growth restriction | Fetal growth | Management |
| **AWMF 015-087 S2e Guideline Monitoring and Care of Twin Pregnancies** | AWMF | 2020 | 36 | DC twin pregnancies with sFGR should be monitored like singletons with FGR. | NS | EK | Intrauterine growth restriction | Fetal growth | Fetal growth charts |
| **AWMF 015-087 S2e Guideline Monitoring and Care of Twin Pregnancies** | AWMF | 2020 | 38 | DC twin pregnancies with sFGR should, according to severity, be monitored with Doppler examinations about every 2 weeks. | NS | EK | Intrauterine growth restriction | Fetal growth | Scanning |
| **Tvillinger - ﻿håndtering af graviditet og fødsel (twins- handling pregnancy and childbirth)** | Sandbjerg | 2010 | NS | The frequency of heart malformations is approx. twice as common in twins compared to singletons (Risk: 2-3%) and possibly even higher in monochorionic twins, especially if they have twin transfusion syndrome, which means that a Fetal heart scan is recommended for monochorionic and should be considered for dichorionic according to http://www.dsog.dk/sandbjerg/foeto/foeto Guideline final .pdf | B | NS | Recommendations with strengths | Fetal growth | Scanning |
| **Tvillinger - ﻿håndtering af graviditet og fødsel (twins- handling pregnancy and childbirth)** | Sandbjerg | 2010 | NS | Screening of dichorionic twins in week 23 and week 28 is recommended for estimated weight discordance with particular emphasis on difference in abdominal circumference. Other growth scans according to the flowchart. At discordance of > 20% or if one or both twins weigh below 2SD of the expected weight for singletons supplementary CTG and flow studies are recommended and growth monitoring should be performed accordingly for fourteen days. The value of a. Cerebri media flow (MCA) in twins is uncertain and since it is difficult to measure, it is initially recommended to measure flow only in the umbilical artery. In case of pathological umbilical flow, supplement with flow in the venous system and possibly MCA. Biophysical profile can be considered especially at low gestational ages. Delivery time for SGA / IUGR / affected flow must be assessed taking into account gestational age for a possibly healthy twin, as well as studies suggesting a longer latency from pathological flow to fetal death in twin pregnancies. | BC | 2 | Recommendations with strengths | Fetal growth | Scanning |
| **Ultrasound for twin and multiple pregnancies** | Toward optimized practice (TOP) | 2017 | NS | Routinely report presence of normal fetal movements, and perform a biophysical profile (BPP) at 28 weeks onward for assessment of fetal well-being and manage as per the Toward Optimized Practice Third Trimester Fetal Well-Being Studies: Criteria and Managing Results CPG. | NS |  | Second and third trimester studies | Fetal growth | Scanning |
| **Ultrasound for twin and multiple pregnancies** | Toward optimized practice (TOP) | 2017 | NS | Routinely report EFW and gender appropriate percentiles for each fetus. | NS |  | Estimated fetal weights (EFW) | Fetal growth | Scanning |
| **Ultrasound for twin and multiple pregnancies** | Toward optimized practice (TOP) | 2017 | NS | Use singleton growth curves for evaluating growth abnormalities. o for 2000-2009 Alberta gender specific live birth weights report2 see: http://www.health.alberta.ca/documents/Reproductive-Health-2011.pdf. o For individual growth charts see Appendices A, B and C. | NS |  | Estimated fetal weights (EFW) | Fetal growth | Fetal growth charts |
| **Ultrasound for twin and multiple pregnancies** | Toward optimized practice (TOP) | 2017 | NS | Use female growth chart if gender unknown. | NS |  | Estimated fetal weights (EFW) | Fetal growth | Fetal growth charts |
| **Ultrasound for twin and multiple pregnancies** | Toward optimized practice (TOP) | 2017 | NS | Define significant growth discordance as the presence of either a >20% difference in ultrasound-derived estimated fetal weight or a (20 mm) absolute measurement difference in abdominal circumference. | NS |  | Estimated fetal weights (EFW) | Fetal growth | Scanning |
| **Ultrasound for twin and multiple pregnancies** | Toward optimized practice (TOP) | 2017 | NS | Calculation for EFW discordance ((EFW larger twin - EFW smaller twin/EFW largest twin) x 100) | NS |  | Estimated fetal weights (EFW) | Fetal growth | Scanning |
| **Ultrasound for twin and multiple pregnancies** | Toward optimized practice (TOP) | 2017 | NS | Increase surveillance and/or refer to MFM if: o Either one or both fetuses EFW <10th percentile o Either one or both fetuses abdominal circumference <10th percentile o Significant growth discordance in EFW or abdominal circumference (as defined above) | NS |  | Estimated fetal weights (EFW) | Fetal growth | Management |
| **Ultrasound for twin and multiple pregnancies** | Toward optimized practice (TOP) | 2017 | NS | Growth discordance in multiples. Requires same or next day referral to MFM. o If there is any delay, MFM must be contacted to advise on and coordinate care. | NS |  | Estimated fetal weights (EFW) | Fetal growth | Management |
| **Ultrasound for twin and multiple pregnancies** | Toward optimized practice (TOP) | 2017 | NS | DCDA: Perform ultrasound assessments at ~12 weeks and ~18 weeks in uncomplicated DCDA pregnancies, and every 3-4 weeks thereafter. | NS |  | Additional requirements for twin/multiple sub-types for DCDC pregnancies | Fetal growth | Scanning |
| **Ultrasound for twin and multiple pregnancies** | Toward optimized practice (TOP) | 2017 | NS | DCDA: More frequent ultrasound assessments may be required if maternal or fetal complications. | NS |  | Additional requirements for twin/multiple sub-types for DCDC pregnancies | Fetal growth | Scanning |
| **Ultrasound for twin and multiple pregnancies** | Toward optimized practice (TOP) | 2017 | NS | DCDA: Consider reporting bladders routinely to avoid misses on unrecognized MCDAs | NS |  | Additional requirements for twin/multiple sub-types for DCDC pregnancies | Fetal growth | Scanning |
| **Ultrasound for twin and multiple pregnancies** | Toward optimized practice (TOP) | 2017 | NS | Same or next day referral to MFM should also be triggered by the following: o Abnormal or significantly discordant nuchal translucencies in the first trimester o IUGR/SGA (EFW or AC <10th percentile) affecting one or more fetuses o Significant growth discordance (>20% difference in EFW or 20 mm difference in ACs) | NS |  | Complications | Fetal growth | Management |
| **Ultrasound for twin and multiple pregnancies** | Toward optimized practice (TOP) | 2017 | NS | Same day referral to hospital (labour and delivery) AND obstetrics/gynecology referral for: o BPP score of 6/8 or less for one or more twins, which may require a non-stress test (NST) | NS |  | Complications | Fetal growth | Management |
| **Management of multiple pregnancy** | SIGO, AOGOI, AGUI | 2016 | NS | For uncomplicated DCDA and MCDA pregnancies, monthly clinical assessment is recommended, which must intensify in the 3rd trim. | B | 6 | Clinical management of uncomplicated multiple pregnancy | Fetal growth | Scanning |
| **Management of multiple pregnancy** | SIGO, AOGOI, AGUI | 2016 | NS | In uncomplicated DCDA pregnancies, US scans are recommended every 4-6 weeks after the 20-week scan. | B | 6 | Clinical management of uncomplicated multiple pregnancy | Fetal growth | Scanning |
| **Management of multiple pregnancy** | SIGO, AOGOI, AGUI | 2016 | NS | In the case of a DCDA twin pregnancy there is no provision for a routine fetal echocardiography. | B | 2 | Maternal and fetal complications | Fetal growth | Scanning |
| **Management of multiple pregnancy** | SIGO, AOGOI, AGUI | 2016 | NS | The definitions of selective IUGR refers to weight estimated less than/equal to 10th percentile in a twin, a weight difference between twins of greater than/equal to 25% or the difference between the 2 abdominal circumferences or greater than 10% in the absence of other complications such as fetal-fetal transfusion (?TTTS). | B | 3 | Maternal and fetal complications | Fetal growth | Scanning |
| **Management of multiple pregnancy** | SIGO, AOGOI, AGUI | 2016 | NS | It is believed that the deviation from the normal growth pattern is considered such after 2 successive surveys (US) in the space of one week or preferably 2 weeks. | C | 4 | Maternal and fetal complications | Fetal growth | Scanning |
| **Twin pregnancies: guidelines for clinical practice from the French College of Gynaecologists and Obstetricians (CNGOF)** | Christophe Vayssiere | 2011 | NS | A monthly ultrasound examination including an estimation of fetal weight and Doppler umbilical artery velocimetry is recommended (Professional Consensus) |  | NS | Professional consensus | Fetal growth | Scanning |
| **Twin pregnancies: guidelines for clinical practice from the French College of Gynaecologists and Obstetricians (CNGOF)** | Christophe Vayssiere | 2011 | NS | More intensive ultrasound monitoring is advised if the weight discordance between the two fetuses exceeds 20–25% (Level B |  | NS | Level B | Fetal growth | Management |
| **Multiple Pregnancy** | Lithuanian Society of Obstetricians and Gynaecologists, Lithuanian Midwives Association | 2014 | 5.4 | Visits for multiple pregnancies without complications are recommended. DCDA Twins: At least 8 visits. Ultrasound examination 11–13, 20, 24, 28, 32, 36 weeks. Ultrasound examination is not required at 16 and 34 weeks. | NS | NS | Antenatal care | Fetal growth | Scanning |
| **Multiple Pregnancy** | Lithuanian Society of Obstetricians and Gynaecologists, Lithuanian Midwives Association | 2014 | 5.9.3 | Unequal growth of twins is diagnosed when the weight of the fetus is measured by ultrasound differs by more than 20% or their abdominal circumference differs by more than 20 mm. If twin growth is uneven, one or both fetuses die more often, and the live fetuses has an increased risk of neurological damage. One or both monochorionic twins growing unevenly, may die suddenly without clear signs of deterioration. When diagnosed with different fetal growth, ultrasound every 1-2 weeks, growth dynamics, AFI, umbilical artery blood flow, biophysical profile is performed | NS | NS | Antenatal care | Fetal growth | Scanning |
| **Role of ultrasonography in the management of twin gestation** | International Federation of Gynecology and Obstetrics (FIGO) | 2018 | NS | In addition to placental location, placental cord insertions should also be evaluated. | NS | NS | Diamniotic/dichorionic twins | Fetal growth | Scanning |
| **Role of ultrasonography in the management of twin gestation** | International Federation of Gynecology and Obstetrics (FIGO) | 2018 | NS | Serial ultrasonography examinations every 4–6 weeks are recommended to assess fetal growth, size concordance, and amniotic fluid volume | NS | NS | Diamniotic/dichorionic twins | Fetal growth | Scanning |
| **Role of ultrasonography in the management of twin gestation** | International Federation of Gynecology and Obstetrics (FIGO) | 2018 | NS | Growth discordance is calculated by the difference in the estimated twin weights divided by the weight of the larger twin, and is expressed as a percentage. | NS | NS | Diamniotic/dichorionic twins | Fetal growth | Scanning |
| **Role of ultrasonography in the management of twin gestation** | International Federation of Gynecology and Obstetrics (FIGO) | 2018 | NS | A growth discordance greater than 20% is associated with a higher incidence of adverse outcomes. | NS | NS | Diamniotic/dichorionic twins | Fetal growth | Scanning |
| **Role of ultrasonography in the management of twin gestation** | International Federation of Gynecology and Obstetrics (FIGO) | 2018 | Table 4 | Second trimester: Anatomy scan at 18-20 weeks, growth scans every 4-6 weeks with more frequent assessment of anomalies present | NS | NS | General guidelines for ultrasonography imaging in twin pregnancies TABLE 4 | Fetal growth | Scanning |
| **Role of ultrasonography in the management of twin gestation** | International Federation of Gynecology and Obstetrics (FIGO) | 2018 | Table 4 | Third trimester: Continue 4-6 weeks growth scans with further assessment as indicated by anomalies | NS | NS | General guidelines for ultrasonography imaging in twin pregnancies TABLE 4 | Fetal growth | Scanning |

**Article Title:** Clinical practice guidelines for the antenatal management of dichorionic diamniotic twin pregnancies: a systematic review.

**Author names:**

Caroline O’Connor^1, 2*^, Emily O’Connor^1, 2, 3^, Sara Leitao^2, 3^, Shauna Barrett^4^, Keelin O’Donoghue^1, 2^

**Affiliations**

^1^ INFANT Research Centre, University College Cork, Cork, Ireland

^2^ Pregnancy Loss Research Group, Department of Obstetrics & Gynecology, University College Cork, Cork, Ireland

^3^ National Perinatal Epidemiology Center (NPEC), University College Cork, Cork, Ireland

^4^ Cork University Hospital Library, Cork University Hospital, Cork, Ireland

**Corresponding author:** *Caroline O’Connor

E-mail: carolineoconnor@ucc.ie
