# Supplementary material for: Clinical practice guidelines for the antenatal management of dichorionic diamniotic twin pregnancies: a systematic review
Source: BMC Pregnancy Childbirth. 2023 May 13;23:347. doi: 10.1186/s12884-023-05652-z (PMC10182673; doi:10.1186/s12884-023-05652-z)
Supplement: Supplementary file 4 — Additional file 4 [file 12884_2023_5652_MOESM4_ESM.docx]

| **Guideline title** | **Author** | **Year** | **Recommendation No.** | **Recommendation** | **Strength of Recommendation** | **Quality of evidence** | **Recommendation category specified within guideline** | **Category** | **Subcategory** |
| --- | --- | --- | --- | --- | --- | --- | --- | --- | --- |
| **ISUOG Practice Guidelines: role of ultrasound in twin pregnancy** | ISUOG | 2016 | NS | ﻿In dichorionic twin pregnancy, selective feticide is performed by ultrasound-guided intracardiac or intra- funicular injection of potassium chloride or lignocaine, preferably in the first trimester. | B | NS | Selective feticide in twin pregnancy | Fetal reduction | TOP |
| **ISUOG Practice Guidelines: role of ultrasound in twin pregnancy** | ISUOG | 2016 | NS | When the diagnosis is made in the second trimester, women might opt for a late selective termination in the third trimester, if the law permits, when the procedure is associated with a risk of preterm birth rather than fetal loss of the unaffected twin. The pros and cons of each option should be considered (prematurity, loss rate, parental stress, availability of a fetal medicine specialist to perform the procedure in the event of preterm labor, and risk of complications associated with the specific anomaly) | NS | 2++ | Selective feticide in twin pregnancy | Fetal reduction | TOP |
| **ISUOG Practice Guidelines: role of ultrasound in twin pregnancy** | ISUOG | 2016 | NS | ﻿If there is a substantial risk of fetal demise of one cotwin before 26 weeks, selective termination may be considered. *NOTE: ﻿Management of these cases is complex and should be coordinated by a tertiary-level fetal medicine centre.* | D | 2- | ﻿Screening, diagnosis and management of fetal growth restriction (FGR). | Fetal reduction | TOP |
| **Prenatal Screening for and Diagnosis of Aneuploidy in Twin Pregnancies: Joint SOGC-CCMG Clinical Practice Guideline No, 262** | SOGC | 2011 | 7 | ﻿Prior to invasive testing or in the context of twins discordant for an abnormality, selective reduction should be discussed and made available to those requesting the procedure after appropriate counselling. | B | III | ﻿MANAGEMENT OF TWINS DISCORDANT FOR KARYOTYPICAL ANOMALIES | Fetal reduction | Selective reduction |
| **Prenatal Screening for and Diagnosis of Aneuploidy in Twin Pregnancies: Joint SOGC-CCMG Clinical Practice Guideline No, 262** | SOGC | 2011 | 8 | ﻿Monitoring for disseminated intravascular coagulopathy is not indicated in dichorionic twin pregnancies undergoing selective reduction. | B | II-2 | Management of twins discordant for karyotypical anomalies | Fetal reduction | Selective reduction |
| **Twin pregnancy** | South Australian Perinatal Practice Guideline | 2018 | NS | Fetal reduction or termination is possible in cases of congenital anomaly in one or both twins. | NS | NS | Antenatal care in pregnancy | fetal reduction | Selective reduction |
| **AWMF 015-087 S2e Guideline Monitoring and Care of Twin Pregnancies** | AWMF | 2020 | 24 | In the case of anomalies in a discordant DC twin pregnancy, embryo reduction can be performed, preferably in the first trimester, by ultrasound-guided intracardiac injection of potassium chloride or lidocaine | C | 2+ | Selective feticide in twin pregnancies | Fetal reduction | Selective reduction |
| **Management of multiple pregnancy** | SIGO, AOGOI, AGUI | 2016 | NS | Doctors should inform the couple about the risks and benefits of embryo reduction. | NS | Level of evidence 6. |  | Fetal reduction | Selective reduction |
| **Management of multiple pregnancy** | SIGO, AOGOI, AGUI | 2016 | NS | The ideal timing of the embryo reduction procedure is between 11+0 and 14+0 weeks gestation. It is possible, within the references of the law, to do a selective reduction of fetuses in higher gestational age. | NS | NS | Embryo or fetal reduction | Fetal reduction | Selective reduction |
| **Twin pregnancies: guidelines for clinical practice from the French College of Gynaecologists and Obstetricians (CNGOF)** | Christophe Vayssiere | 2011 | NS | In the case of an especially severe malformation in a dichorionic twin, selective pregnancy reduction is possible and does not present a direct risk to the healthy twin (Professional Consensus). | NS | NS | Professional consensus | Fetal reduction | Selective reduction |
| **Multiple Pregnancy** | Lithuanian Society of Obstetricians and Gynaecologists, Lithuanian Midwives Association | 2014 | 5.8.3 | When the pathology of the fetal structure is diagnosed, potassium is possible in the case of dichorionic twins chloride injection into the heart of the affected twin, and in the case of monochorionic twins laser coagulation of the damaged fetal umbilical cord is performed | NS | NS | Antenatal care | Fetal reduction | TOP |
| **Multifetal Pregnancy Reduction: Committee Opinion No. 719** | The American College of Obstetricians and Gynecologists (ACOG) | 2017 | NS | During patient counselling, physicians should consider discussing reduction to a singleton pregnancy based on their understanding of the particular patient, her unique medical situation and her values. When a woman with a twin gestation requests such information, weather for medical or non-medical reasons, it should be provided in a timely manner and without bias. | NS | NS | Reduction to a singleton | Fetal reduction | MFPR |

**Article Title:** Clinical practice guidelines for the antenatal management of dichorionic diamniotic twin pregnancies: a systematic review.

**Author names:**

Caroline O’Connor^1, 2*^, Emily O’Connor^1, 2, 3^, Sara Leitao^2, 3^, Shauna Barrett^4^, Keelin O’Donoghue^1, 2^

**Affiliations**

^1^ INFANT Research Centre, University College Cork, Cork, Ireland

^2^ Pregnancy Loss Research Group, Department of Obstetrics & Gynecology, University College Cork, Cork, Ireland

^3^ National Perinatal Epidemiology Center (NPEC), University College Cork, Cork, Ireland

^4^ Cork University Hospital Library, Cork University Hospital, Cork, Ireland

**Corresponding author:** *Caroline O’Connor

E-mail: carolineoconnor@ucc.ie
