# Supplementary material for: Clinical practice guidelines for the antenatal management of dichorionic diamniotic twin pregnancies: a systematic review
Source: BMC Pregnancy Childbirth. 2023 May 13;23:347. doi: 10.1186/s12884-023-05652-z (PMC10182673; doi:10.1186/s12884-023-05652-z)
Supplement: Supplementary file 5 — Additional file 5 [file 12884_2023_5652_MOESM5_ESM.docx]

| **Guideline title** | **Author** | **Year** | **Recommendation No.** | **Recommendation** | **Strength of Recommendation** | **Quality of evidence** | **Recommendation category specified within guideline** | **Category** | **Subcategory** |
| --- | --- | --- | --- | --- | --- | --- | --- | --- | --- |
| Multifetal Gestations: Twin, Triplet, and Higher-Order Multifetal Pregnancies (Practice Bulletin No 231). | ACOG | 2021 | NS | Management of the death of one fetus: Care should be individualized for each patient, and consultation with a physician with training in maternal–fetal medicine is recommended. In the event that a twin pregnancy is diagnosed late enough that chorionicity cannot be established, management should be guided by individualized assessment of fetal growth, growth discordance, and other indicators of fetal well-being. | NS | NS | None | Fetal death | Management |
| ISUOG Practice Guidelines: role of ultrasound in twin pregnancy | ISUOG | 2016 | NS | ﻿When single IUD occurs in a twin pregnancy, the woman should be referred to a tertiary-level center with relevant expertise. | Good practice point | NS | ﻿Managing the surviving twin after demise of its cotwin. | Fetal death | Management |
| Ultrasound in twin pregnancies: SOGC Clinical practice guideline No. 260 | SOGC | 2011 | Summary statement 8 | ﻿Referral to an appropriate high-risk pregnancy centre is indicated when complications unique to twins are suspected on ultrasound. *NOTE: These complications include: Single fetal death in the second or third trimester* | NS | II-2 | Single fetal death | Fetal death | Management |
| Ultrasound for twin and multiple pregnancies | Toward optimized practice (TOP) | 2017 | NS | Same day referral to MFM should be triggered for the following: Single (or impending) fetal death in the second or third trimester (most commonly associated with growth discordance). | NS | NS | Complications | Fetal death | Management |
| Management of multiple pregnancy | SIGO, AOGOI, AGUI | 2016 | NS | In case of IUD of one twin, conservative management is recommended, with labour and delivery to be carried out preferably at the end of gestation independent/regardless of chorionicity. | C | 6 | Stillbirth of a twin | Fetal death | Management |
| Management of multiple pregnancy | SIGO, AOGOI, AGUI | 2016 | NS | In case of a twin IUD, it is necessary to monitor the maternal parameters of coagulation. | C | 5 | Stillbirth of a twin | Fetal death | Management |
| Management of multiple pregnancy | SIGO, AOGOI, AGUI | 2016 | NS | In case of a twin's IUD it is recommended anti-D immunoglobulin in women who are Rh group neg and not isoimmunised. | C | 5 | Stillbirth of a twin | Fetal death | Management |
| Management of multiple pregnancy | SIGO, AOGOI, AGUI | 2016 | NS | It is recommended to send to these cases of IUD to reference (specialist/tertiary) centers. | B | 6 | Stillbirth of a twin | Fetal death | Management |
| Management of multiple pregnancy | SIGO, AOGOI, AGUI | 2016 | NS | Care in these cases must be individualized on the basis of possible risks, contraindications and the clinical and laboratory picture. | C | 6 | Stillbirth of a twin | Fetal death | Management |
| Twin pregnancies: guidelines for clinical practice from the French College of Gynaecologists and Obstetricians (CNGOF) | Christophe Vayssiere | 2011 | NS | Psychological counselling is recommended at the death of a twin(Professional Consensus). | NS | NS | Professional consensus | Fetal death | Counselling |
| Multiple Pregnancy | Lithuanian Society of Obstetricians and Gynaecologists, Lithuanian Midwives Association | 2014 | 5.10.2 | Death of a twin: In the case of dichorionic twins, the dead twin usually mummifies and the other continues to grow without complications. Surviving twins can die in 5-10 percent of cases. The condition of the remaining living twin is monitored, maturity and spontaneous delivery are expected. | NS | NS | Antenatal care | Fetal death | Management |
| Multiple Pregnancy | Lithuanian Society of Obstetricians and Gynaecologists, Lithuanian Midwives Association | 2014 | 5.10.4 | Blood coagulation disorders in pregnant women - hypofibrinogenemia or disseminated intravascular coagulopathy after death of one of the fetuses is rare. However it is recommended that blood clotting tests be performed every 1 to 2 weeks | NS | NS | Antenatal care | Fetal death | Management |
| Multiple Pregnancy | Lithuanian Society of Obstetricians and Gynaecologists, Lithuanian Midwives Association | 2014 | 5.10.5 | A pregnant woman who is Rh factor negative and does not have an immunization after the death of one fetus, immunoglobulin prophylaxis is required | NS | NS | Antenatal care | Fetal death | Management |

**Article Title:** Clinical practice guidelines for the antenatal management of dichorionic diamniotic twin pregnancies: a systematic review.

**Author names:**

Caroline O’Connor^1, 2*^, Emily O’Connor^1, 2, 3^, Sara Leitao^2, 3^, Shauna Barrett^4^, Keelin O’Donoghue^1, 2^

**Affiliations**

^1^ INFANT Research Centre, University College Cork, Cork, Ireland

^2^ Pregnancy Loss Research Group, Department of Obstetrics & Gynecology, University College Cork, Cork, Ireland

^3^ National Perinatal Epidemiology Center (NPEC), University College Cork, Cork, Ireland

^4^ Cork University Hospital Library, Cork University Hospital, Cork, Ireland

**Corresponding author:** *Caroline O’Connor

E-mail: carolineoconnor@ucc.ie
