# Supplementary material for: Clinical practice guidelines for the antenatal management of dichorionic diamniotic twin pregnancies: a systematic review
Source: BMC Pregnancy Childbirth. 2023 May 13;23:347. doi: 10.1186/s12884-023-05652-z (PMC10182673; doi:10.1186/s12884-023-05652-z)
Supplement: Supplementary file 6 — Additional file 6 [file 12884_2023_5652_MOESM6_ESM.docx]

| **Guideline title** | **Author** | **Year** | **Recommendation No.** | **Recommendation** | **Strength of Recommendation** | **Quality of evidence** | **Recommendation category specified within guideline** | **Category** | **Subcategory** |
| --- | --- | --- | --- | --- | --- | --- | --- | --- | --- |
| **Multifetal Gestations: Twin, Triplet, and Higher-Order Multifetal Pregnancies (Practice Bulletin No 231).** | ACOG | 2021 | NS | In multifetal gestations, if a fetal demise, vanishing twin, or anomaly is identified in one fetus, there is a significant risk of an inaccurate test result if serum based aneuploidy screening or cell-free DNA is used. This information should be reviewed with the patient and diagnostic testing should be offered. | NS | NS | None | Screening for fetal anomalies | Screening for aneuploidy |
| **Multifetal Gestations: Twin, Triplet, and Higher-Order Multifetal Pregnancies (Practice Bulletin No 231).** | ACOG | 2021 | NS | All women with multifetal gestations, regardless of age, are candidates for routine screening for fetal chromosomal abnormalities. | Level C | NS | None | Screening for fetal anomalies | Screening for aneuploidy |
| **Multifetal Gestations: Twin, Triplet, and Higher-Order Multifetal Pregnancies (Practice Bulletin No 231).** | ACOG | 2021 | NS | Amniocentesis and CVS can be performed in women with a multifetal gestation who desire definitive testing for genetic anomalies. | NS | NS | None | Screening for fetal anomalies | Screening for aneuploidy |
| **Multifetal Gestations: Twin, Triplet, and Higher-Order Multifetal Pregnancies (Practice Bulletin No 231).** | ACOG | 2021 | NS | When a chromosomal abnormality is diagnosed, counselling should include a discussion of options for pregnancy management if only one fetus is found to be affected. These options include terminating the entire pregnancy; selective reduction of the affected fetus; and continuing the pregnancy without any intervention. | NS | NS | None | Screening for fetal anomalies | Counselling |
| **ISUOG Practice Guidelines: role of ultrasound in twin pregnancy** | ISUOG | 2016 | NS | ﻿Screening for trisomy 21 can be performed in the first trimester using the combined test (nuchal translucency thickness (NT), free beta-human chorionic gonadotropin (β-hCG) level and pregnancy-associated plasma protein-A (PAPP-A) level). An alternative is combination of maternal age and NT only. | B | NS | Screening for chromosomal abnormalities in twin pregnancy | Screening for fetal anomalies | Screening for aneuploidy |
| **ISUOG Practice Guidelines: role of ultrasound in twin pregnancy** | ISUOG | 2016 | NS | ﻿In case of a vanished twin, if there is still a measurable fetal pole, NT alone, in combination with maternal age, should be used for risk estimation. | B | None | Screening for chromosomal abnormalities in twin pregnancy | Screening for fetal anomalies | Screening for aneuploidy |
| **ISUOG Practice Guidelines: role of ultrasound in twin pregnancy** | ISUOG | 2016 | NS | Screening and diagnostic testing for trisomy is more complex in twin compared with singleton pregnancy. It is important, therefore, that counselling prior to testing is provided by healthcare professionals with expertise in this area. It is important to inform women and their partners in advance of the potentially complex decisions that they will need to make on the basis of the results of combined screening, bearing in mind the increased risk of invasive testing in twins, the possible discordance between dichorionic twins for fetal aneuploidy, and the risks of selective fetal reduction. | NS | 2+ |  | Screening for fetal anomalies | Counselling |
| **ISUOG Practice Guidelines: role of ultrasound in twin pregnancy** | ISUOG | 2016 | NS | ﻿CVS is preferred in dichorionic twin pregnancy. *NOTE: ﻿Invasive testing for chromosomal or genetic analysis of twins should be carried out by a fetal medicine expert. CVS is preferred in dichorionic twin pregnancy because it can be performed earlier than can amniocentesis. Earlier diagnosis of any aneuploidy is particularly important in twin pregnancy, given the lower risk of selective termination in the first compared with the second trimester (7% risk of loss of the entire pregnancy, and 14% risk of delivery before 32 weeks).* | D | 3 | ﻿Invasive prenatal diagnosis in twin pregnancy. | Screening for fetal anomalies | Screening for aneuploidy |
| **ISUOG Practice Guidelines: role of ultrasound in twin pregnancy** | ISUOG | 2016 | NS | Twin fetuses should be assessed for the presence of any major anomalies at the first-trimester scan, and a routine second-trimester (anomaly) scan should be performed at around 20 (18–22) weeks’ gestation | GOOD PRACTICE POINT | NS | Ultrasound screening for structural abnormalities in twin pregnancy | Screening for fetal anomalies | Management |
| **ISUOG Practice Guidelines: role of ultrasound in twin pregnancy** | ISUOG | 2016 | NS | Twin pregnancies discordant for fetal anomaly should be referred to a regional fetal medicine center | GOOD PRACTICE POINT | NS | Ultrasound screening for structural abnormalities in twin pregnancy | Screening for fetal anomalies | Management |
| **Ultrasound in twin pregnancies: SOGC Clinical practice guideline No. 260** | SOGC | 2011 | 7 | ﻿In twin pregnancies, aneuploidy screening using nuchal translucency measurements should be offered. | B | II-2 | Screening for anomalies in twin pregnancies | Screening for fetal anomalies | Screening for aneuploidy |
| **Ultrasound in twin pregnancies: SOGC Clinical practice guideline No. 260** | SOGC | 2011 | 8 | ﻿Detailed ultrasound examination to screen for fetal anomalies should be offered, preferably between 18 and 22 weeks’ gestation, in all twin pregnancies. | B | II-2 | Congenital malformations | Screening for fetal anomalies | 2nd trimester anomaly scan |
| **Ultrasound in twin pregnancies: SOGC Clinical practice guideline No. 260** | SOGC | 2011 | Summary statement 1 | ﻿There are insufficient data to make recommendations on repeat anatomical assessments in twin pregnancies. Therefore, a complete anatomical survey at each scan may not be needed following a complete and normal assessment. | NS | III | Congenital malformations | Screening for fetal anomalies | Management |
| **Prenatal Screening for and Diagnosis of Aneuploidy in Twin Pregnancies: Joint SOGC-CCMG Clinical Practice Guideline No, 262** | SOGC | 2011 | 1 | ﻿All pregnant women in Canada, regardless of age, should be offered, through an informed counselling process, the option of a prenatal screening test for the most common clinically significant fetal aneuploidies. In addition, they should be offered a second trimester ultrasound for dating, assessment of fetal anatomy, and detection of multiples. | A | I | Prenatal screening in twins | Screening for fetal anomalies | Counselling |
| **Prenatal Screening for and Diagnosis of Aneuploidy in Twin Pregnancies: Joint SOGC-CCMG Clinical Practice Guideline No, 262** | SOGC | 2011 | 2 | ﻿Counselling must be non-directive and must respect a woman’s right to accept or decline any or all of the testing or options offered at any point in the process. | A | III | Prenatal screening in twins | Screening for fetal anomalies | Counselling |
| **Prenatal Screening for and Diagnosis of Aneuploidy in Twin Pregnancies: Joint SOGC-CCMG Clinical Practice Guideline No, 262** | SOGC | 2011 | 3 | ﻿When non-invasive prenatal screening for aneuploidy is available, maternal age alone should not be an indication for invasive prenatal diagnosis in a twin pregnancy. | A | II-2 | Prenatal screening in twins | Screening for fetal anomalies | Screening for aneuploidy |
| **Prenatal Screening for and Diagnosis of Aneuploidy in Twin Pregnancies: Joint SOGC-CCMG Clinical Practice Guideline No, 262** | SOGC | 2011 | 3 | ﻿If non-invasive prenatal screening is not available, invasive prenatal diagnosis in twins should be offered to women aged 35 and over. | B | II-2 | Prenatal screening in twins | Screening for fetal anomalies | Screening for aneuploidy |
| **Prenatal Screening for and Diagnosis of Aneuploidy in Twin Pregnancies: Joint SOGC-CCMG Clinical Practice Guideline No, 262** | SOGC | 2011 | Summary statement 1 | ﻿Fetal nuchal translucency combined with maternal age is an acceptable first trimester screening test for aneuploidies in twin pregnancies. | NS | II-2 | Prenatal screening in twins | Screening for fetal anomalies | Screening for aneuploidy |
| **Prenatal Screening for and Diagnosis of Aneuploidy in Twin Pregnancies: Joint SOGC-CCMG Clinical Practice Guideline No, 262** | SOGC | 2011 | 5 | ﻿When screening is done by nuchal translucency and maternal age, a pregnancy-specific risk should be calculated in monochorionic twins. In dichorionic twins, a fetus-specific risk should be calculated. | C | II-3 | Prenatal screening in twins | Screening for fetal anomalies | Screening for aneuploidy |
| **Prenatal Screening for and Diagnosis of Aneuploidy in Twin Pregnancies: Joint SOGC-CCMG Clinical Practice Guideline No, 262** | SOGC | 2011 | Summary statement 2 | ﻿First trimester serum screening combined with nuchal translucency may be considered in twin pregnancies. It provides some improvement over the performance of screening by nuchal translucency and maternal age by decreasing the false-positive rate. | NS | II-3 | Prenatal screening in twins | Screening for fetal anomalies | Screening for aneuploidy |
| **Prenatal Screening for and Diagnosis of Aneuploidy in Twin Pregnancies: Joint SOGC-CCMG Clinical Practice Guideline No, 262** | SOGC | 2011 | Summary statement 3 | ﻿Integrated screening with nuchal translucency plus first and second trimester serum screening is an option in twin pregnancies. Further prospective studies are required in this area since it has not been validated in prospective studies in twins. | NS | III | Prenatal screening in twins | Screening for fetal anomalies | Screening for aneuploidy |
| **Prenatal Screening for and Diagnosis of Aneuploidy in Twin Pregnancies: Joint SOGC-CCMG Clinical Practice Guideline No, 262** | SOGC | 2011 | Summary statement 4 | ﻿Non-directive counselling is essential when invasive testing is offered. | NS | III | Invasive prenatal diagnostics for twin pregnancies | Screening for fetal anomalies | Counselling |
| **Prenatal Screening for and Diagnosis of Aneuploidy in Twin Pregnancies: Joint SOGC-CCMG Clinical Practice Guideline No, 262** | SOGC | 2011 | Summary statement 5 | ﻿When chorionic villus sampling is performed in non- monochorionic multiple pregnancies, a combination of transabdominal and transcervical approaches or a transabdominal only approach appears to provide the best results to minimize the likelihood of sampling errors. | NS | II-2 | Chorionic villus sampling | Screening for fetal anomalies | Screening for aneuploidy |
| **Twin and Triplet Pregnancy: NG137** | NICE | 2019 | 1.2.1 | ﻿Explain sensitively the aims and possible outcomes of all screening and ﻿diagnostic tests to women with a twin or triplet pregnancy to minimise their anxiety. | NS | NS | Information and emotional support | Screening for fetal anomalies | Counselling |
| **Twin and Triplet Pregnancy: NG137** | NICE | 2019 | 1.4.1 | A healthcare professional with experience of caring for women with twin and triplet pregnancies should offer information and counselling to women before and after every screening test. | NS | NS | Information about screening | Screening for fetal anomalies | Screening for aneuploidy |
| **Twin and Triplet Pregnancy: NG137** | NICE | 2019 | 1.4.2 | ﻿Inform women with a twin or triplet pregnancy about the complexity of decisions they may need to make depending on the outcomes of screening, including different options according to the chorionicity and amnionicity of the pregnancy | NS | NS | Information about screening | Screening for fetal anomalies | Counselling |
| **Twin and Triplet Pregnancy: NG137** | NICE | 2019 | 1.4.3 | ﻿Offer women with a twin pregnancy information on and screening for Down's syndrome, Edwards' syndrome and Patau's syndrome as outlined in the NHS fetal anomaly screening programme (FASP). | NS | NS | Screening for chromosomal conditions: twin pregnancy | Screening for fetal anomalies | Screening for aneuploidy |
| **Twin and Triplet Pregnancy: NG137** | NICE | 2019 | 1.4.9 | Offer screening for structural abnormalities (such as cardiac abnormalities) in twin and triplet pregnancies as in routine antenatal care; see NICE's guideline on antenatal care for uncomplicated pregnancies and the NHS fetal anomaly screening programme. | NS | NS | Screening for structural abnormalities | Screening for fetal anomalies | 2nd Trimester anomaly scan |
| **Twin and Triplet Pregnancy: NG137** | NICE | 2019 | 1.4.11 | ﻿Allow 45 minutes for the anomaly scan in twin and triplet pregnancies (as recommended by FASP). | NS | NS | Screening for structural abnormalities | Screening for fetal anomalies | 2nd Trimester anomaly scan |
| **Twin pregnancy** | South Australian Perinatal Practice Guideline | 2018 | NS | Screening for Down syndrome by mid-trimester screening is not applicable to twin pregnancies. Where available, non-invasive fetal diagnosis can be applied in twin pregnancy. Nuchal translucency can be applied for screening. Chorionic villus sampling or amniocentesis can be used as diagnostic tests. However, reported loss rates are greater in sampling a twin pregnancy (possibly due to double puncture) and there is a possibility of inaccurate diagnosis due to sampling the same sac twice. | NS | NS | Antenatal care in pregnancy | Screening for fetal anomalies | Screening for aneuploidy |
| **International Society for Prenatal Diagnosis Position Statement: cell free (cf)DNA screening for Down syndrome in multiple pregnancies** | International Society for Prenatal Diagnosis (ISPD) | 2021 | 1 | The use of first trimester cfDNA screening for the common autosomal trisomies is appropriate for twin pregnancies due to sufficient evidence showing high detection and low false positive rates with high predictive values. | Moderate | NS | Summary of evidence-based practices | Screening for fetal anomalies | Screening for anueploidy |
| **International Society for Prenatal Diagnosis Position Statement: cell free (cf)DNA screening for Down syndrome in multiple pregnancies** | International Society for Prenatal Diagnosis (ISPD) | 2021 | 2 | The finding of an increased risk on a cfDNA screening test in multiple pregnancies should be followed by counselling and an offer of diagnostic testing to confirm results. | Strong | NS | Summary of evidence-based practices | Screening for fetal anomalies | Screening for aneuploidy |
| **International Society for Prenatal Diagnosis Position Statement: cell free (cf)DNA screening for Down syndrome in multiple pregnancies** | International Society for Prenatal Diagnosis (ISPD) | 2021 | 3 | It is preferable for laboratories performing cfDNA testing in multi-fetal pregnancies to take evidence of zygosity into consideration (eg, chorionicity, sex of the fetuses, embryo transfer history) for the interpretation of both test results and fetal fractions. | Moderate | NS | Summary of evidence-based practices | Screening for fetal anomalies | Screening for aneuploidy |
| **International Society for Prenatal Diagnosis Position Statement: cell free (cf)DNA screening for Down syndrome in multiple pregnancies** | International Society for Prenatal Diagnosis (ISPD) | 2021 | 4 | When a cfDNA test failure occurs consider ultrasound and diagnostic testing. If there is sufficient time, a second sample draw may also be considered. | Moderate | NS | Summary of evidence-based practices | Screening for fetal anomalies | Screening for aneuploidy |
| **FIGO Good clinical practice advice: management of twin pregnancy** | FIGO | 2019 | NS | Offer screening in the first trimester using combined test or combination of maternal age and NT or cell-free DNA. | NS | NS | Screening for and prenatal diagnosis of aneuploidy | Screening for fetal anomalies | Screening for aneuploidy |
| **FIGO Good clinical practice advice: management of twin pregnancy** | FIGO | 2019 | NS | If a “vanished twin” occurs, if there is still a visible fetal pole on US, NT alone should be used as HCG and PAPPA levels might be affected by the "vanishing twin". Cell-free DNA might be less accurate in this situation and can lead to a false positive result and should not be recommended. | NS | NS | Screening for and prenatal diagnosis of aneuploidy | Screening for fetal anomalies | Screening for aneuploidy |
| **FIGO Good clinical practice advice: management of twin pregnancy** | FIGO | 2019 | NS | CVS is preferable in DC twins because it can be performed earlier and a result obtained earlier than amnio. | NS | NS | Screening for and prenatal diagnosis of aneuploidy | Screening for fetal anomalies | Screening for aneuploidy |
| **FIGO Good clinical practice advice: management of twin pregnancy** | FIGO | 2019 | NS | All twins with discordant anomaly should be referred to tertiary centre for further management. | NS | NS | Twin pregnancies discordant for fetal anomaly | Screening for fetal anomalies | Management |
| **FIGO Good clinical practice advice: management of twin pregnancy** | FIGO | 2019 | NS | Where there is a potentially lethal abnormality of one foetus, conservative management is preferable in DC twins. | NS | NS | Twin pregnancies discordant for fetal anomaly | Screening for fetal anomalies | Management |
| **AWMF 015-087 S2e Guideline Monitoring and Care of Twin Pregnancies** | AWMF | 2020 | 12 | First-trimester screening for chromosomal abnormalities in twins should include maternal age, nuchal translucency (NT) and serum biochemistry (free beta-hCG and PAPP-A). ▪ If necessary, it should be combined with sonographic markers for chromosomal defects such as the nasal bone, tricuspid regurgitation and ductus venosus (NB, TR, DV) | NS | EK, 2++, 2+, 2+ | Screening for chromosomal disorders in twin pregnancies | Screening for fetal anomalies | Screening for aneuploidy |
| **AWMF 015-087 S2e Guideline Monitoring and Care of Twin Pregnancies** | AWMF | 2020 | 13 | In the case of a “vanishing twin”, first-trimester screening for fetal trisomy should take into account maternal age, fetal NT measurement and serum beta-hCG (without PAPP-A) level. PAPP-A should only be used if it has been adjusted for the interval between the estimated gestational age at fetal death and blood collection | B | 2++ | Screening for chromosomal disorders in twin pregnancies | Screening for fetal anomalies | Screening for aneuploidy |
| **AWMF 015-087 S2e Guideline Monitoring and Care of Twin Pregnancies** | AWMF | 2020 | 15 | An US between 11-13+6 weeks should include early structured malformation diagnostics including NT. | NS | EK | Screening for chromosomal disorders in twin pregnancies | Screening for fetal anomalies | Screening for aneuploidy |
| **AWMF 015-087 S2e Guideline Monitoring and Care of Twin Pregnancies** | AWMF | 2020 | 17 | The screening and the invasive diagnostic interventions are more complex in twins than in singletons. These should be done by doctors experienced in the field. Patients should be advised about the risks of the puncture, possible discordance for aneuploidies, potential management strategies and the risks of embryo reduction or selective feticide. | NS | EK | Screening for chromosomal disorders in twin pregnancies | Screening for fetal anomalies | Screening for aneuploidy |
| **AWMF 015-087 S2e Guideline Monitoring and Care of Twin Pregnancies** | AWMF | 2020 | 18 | CVS should be the preferred method for DC twins as it can be employed earlier than amniocentesis. Early diagnosis of aneuploidy is particularly important in twin pregnancies, as the risk of selective fetocide is lower in the first trimester than in the second | C | 2+ | Invasive prenatal diagnosis for twin pregnancies | Screening for fetal anomalies | Screening for aneuploidy |
| **AWMF 015-087 S2e Guideline Monitoring and Care of Twin Pregnancies** | AWMF | 2020 | 19 | The localization of the fetuses and placentas should be carefully mapped to provide a unique assignment. DC twins should be sampled individually. | C | EK, 2+, 3, 2+ | Invasive prenatal diagnosis for twin pregnancies | Screening for fetal anomalies | Screening for aneuploidy |
| **AWMF 015-087 S2e Guideline Monitoring and Care of Twin Pregnancies** | AWMF | 2020 | 21 | Twin fetuses should be screened for the presence of severe malformations in the 1st trimester ultrasound. Organ screening (anatomy scan) should be carried out around 20 (18-22) weeks gestation, including fetal echocardiography. | NS | EK, EK, EK | Ultrasound screening for structural abnormalities | Screening for fetal anomalies | 2nd Trimester anomaly scan |
| **AWMF 015-087 S2e Guideline Monitoring and Care of Twin Pregnancies** | AWMF | 2020 | 23 | A twin pregnancy discordant for fetal malformations should be referred to a fetal medicine center | NS | EK | The management of twin pregnancies with discordant fetal malformations | Screening for fetal anomalies | 2nd Trimester anomaly scan |
| **Ultrasound for twin and multiple pregnancies** | Toward optimized practice (TOP) | 2017 | NS | Offer/recommend aneuploidy screening using a nuchal translucency-based screen between 11-14 weeks gestation. | NS |  | PREGNANCY DATING AND FIRST TRIMESTER ULTRASOUND ASSESSMENT | Screening for fetal anomalies | Screening for aneuploidy |
| **Ultrasound for twin and multiple pregnancies** | Toward optimized practice (TOP) | 2017 | NS | As per routine, offer detailed ultrasound examination to screen for fetal anomalies, preferably between 18 and 20 weeks gestation, in all multiple gestations. | NS |  | Second and third trimester studies | Screening for fetal anomalies | 2nd Trimester anomaly scan |
| **Management of multiple pregnancy** | SIGO, AOGOI, AGUI | 2016 | NS | Before the screening test or invasive procedure counselling is indicated, the purpose of which is explained to the patient/couple the scope proposed exams, the accuracy and limitations, complications and risks, response times and the possible clinical implications of each clinical procedure. | A | 6 | Combined 1st Trim screening in multiple pregnancy | Screening for fetal anomalies | Counselling |
| **Management of multiple pregnancy** | SIGO, AOGOI, AGUI | 2016 | NS | The multiples of the median of the biochemical analysis in maternal blood (PAPP-A and free beta HCG) should be adjusted for twinning, the type of twinning and the type of conception. | A | 3 | Combined 1st Trim screening in multiple pregnancy | Screening for fetal anomalies | Screening for aneuploidy |
| **Management of multiple pregnancy** | SIGO, AOGOI, AGUI | 2016 | NS | In the event of a *vanishing twin* occurring after 8-9 weeks with visible embryonic pole, performing the biochemical test is not recommended. The calculation of risk must be based on the maternal age and nuchal translucency measure. Potentially/Eventually other US markers of level II may be used). | B | 6 | Combined 1st Trim screening in multiple pregnancy | Screening for fetal anomalies | Screening for aneuploidy |
| **Management of multiple pregnancy** | SIGO, AOGOI, AGUI | 2016 | NS | There is currently no sufficient data to recommend the use of NIPT in twin pregnancies. | D | 3 | Combined 1st Trim screening in multiple pregnancy | Screening for fetal anomalies | Screening for aneuploidy |
| **Management of multiple pregnancy** | SIGO, AOGOI, AGUI | 2016 | NS | Invasive prenatal diagnosis should be carried out by an expert operator. | A | 6 | Invasive prenatal diagnosis in multiple pregnancy | Screening for fetal anomalies | Screening for aneuploidy |
| **Management of multiple pregnancy** | SIGO, AOGOI, AGUI | 2016 | NS | In dichorionic twin pregnancies it is possible to have a discordant result, which may lead to a selective reduction and therefore CVS is to be considered the technique of choice. In fact, the embryo/fetal reduction at an earlier time is burdened by a lower rate of pregnancy loss. | A | 3 | Invasive prenatal diagnosis in multiple pregnancy | Screening for fetal anomalies | Screening for aneuploidy |
| **Management of multiple pregnancy** | SIGO, AOGOI, AGUI | 2016 | NS | In the case of DCDA pregnancy, both for the CVS and for the amniocentesis the double puncture is the recommended technique. | A | 6 | Invasive prenatal diagnosis in multiple pregnancy | Screening for fetal anomalies | Screening for aneuploidy |
| **Management of multiple pregnancy** | SIGO, AOGOI, AGUI | 2016 | NS | If the operator chooses to use a dye for performing the amniocentesis, the use of indigo-carmine is indicated instead of using methylene blue. | C | 4 | Invasive prenatal diagnosis in multiple pregnancy | Screening for fetal anomalies | Screening for aneuploidy |
| **Management of multiple pregnancy** | SIGO, AOGOI, AGUI | 2016 | NS | Chorionic villus sampling must be performed with the same technique as that used in single pregnancy, using the route transabdominal, burdened by minor complications compared to the transcervical one | B | 2b | Invasive prenatal diagnosis in multiple pregnancy | Screening for fetal anomalies | Screening for aneuploidy |
| **Management of multiple pregnancy** | SIGO, AOGOI, AGUI | 2016 | NS | In case of multiple pregnancy it is suggested a detailed study of fetal anatomy at the time of 2nd trim US. | B | 4 | Maternal and fetal complications | Screening for fetal anomalies | 2nd Trimester anomaly scan |
| **Twin pregnancies: guidelines for clinical practice from the French College of Gynaecologists and Obstetricians (CNGOF)** | Christophe Vayssiere | 2011 | NS | It appears legitimate to use the risk estimate tables for aneuploidy established for singletons in everyday practice (Professional Consensus). |  | NS | Professional consensus | Screening for fetal anomalies | Screening for aneuploidy |
| **Twin pregnancies: guidelines for clinical practice from the French College of Gynaecologists and Obstetricians (CNGOF)** | Christophe Vayssiere | 2011 | NS | In dichorionic pregnancies, this risk is estimated during the first trimester by a calculation that integrates maternal age and the measurement of the crown-rump length(CRL)and of the nuchal fold of each fetus (Level B) |  | NS | Level B | Screening for fetal anomalies | Screening for aneuploidy |
| **Twin pregnancies: guidelines for clinical practice from the French College of Gynaecologists and Obstetricians (CNGOF)** | Christophe Vayssiere | 2011 | NS | Nor is it recommended to order serum marker tests routinely during the second trimester, because the mean sensitivity is associated with a high false-positive rate and the screening test does not provide the separate risk for each fetus (Professional Consensus). |  | NS | Professional consensus | Screening for fetal anomalies | Screening for aneuploidy |
| **Twin pregnancies: guidelines for clinical practice from the French College of Gynaecologists and Obstetricians (CNGOF)** | Christophe Vayssiere | 2011 | NS | In the case of a choice about sampling methods, chorionic villus sampling is recommended over amniocentesis (Professional Consensus) |  | NS | Professional consensus | Screening for fetal anomalies | Screening for aneuploidy |
| **Twin pregnancies: guidelines for clinical practice from the French College of Gynaecologists and Obstetricians (CNGOF)** | Christophe Vayssiere | 2011 | NS | Its performance, between 11 and 14 weeks, provides an earlier result than amniocentesis and makes it possible to perform selective pregnancy reduction with less risk (Professional Consensus) |  | NS | Professional consensus | Screening for fetal anomalies | Screening for aneuploidy |
| **Twin pregnancies: guidelines for clinical practice from the French College of Gynaecologists and Obstetricians (CNGOF)** | Christophe Vayssiere | 2011 | NS | Sampling from a twin pregnancy must be performed by an operator experienced in taking these samples in multiple pregnancies (Professional Consensus) |  | NS | Professional consensus | Screening for fetal anomalies | Screening for aneuploidy |
| **Twin pregnancies: guidelines for clinical practice from the French College of Gynaecologists and Obstetricians (CNGOF)** | Christophe Vayssiere | 2011 | NS | When amniocentesis is performed, the choice of inserting one needle or two is left to the operator (Professional Consensus) |  | NS | Professional consensus | Screening for fetal anomalies | Screening for aneuploidy |
| **Twin pregnancies: guidelines for clinical practice from the French College of Gynaecologists and Obstetricians (CNGOF)** | Christophe Vayssiere | 2011 | NS | Routine sampling of both fetuses is not always necessary. Nonetheless, parental request justifies it, even in situations where a single sample might otherwise seem sufficient (Professional Consensus). |  | NS | Professional consensus | Screening for fetal anomalies | Screening for aneuploidy |
| **Twin pregnancies: guidelines for clinical practice from the French College of Gynaecologists and Obstetricians (CNGOF)** | Christophe Vayssiere | 2011 | NS | In chorionic villus sampling, the transabdominal route should be preferred to the cervical (Professional Consensus) |  | NS | Professional consensus | Screening for fetal anomalies | Screening for aneuploidy |
| **Twin pregnancies: guidelines for clinical practice from the French College of Gynaecologists and Obstetricians (CNGOF)** | Christophe Vayssiere | 2011 | NS | The incidence of malformations in dichorionic and monochorionic pregnancies is respectively double and triple that in singleton pregnancies. They should be managed at a prenatal diagnostic centre (Professional Consensus). |  | NS | Professional consensus | Screening for fetal anomalies | Management |
| **Multiple Pregnancy** | Lithuanian Society of Obstetricians and Gynaecologists, Lithuanian Midwives Association | 2014 | 5.7.1 | Check for down syndrome: It is performed during the first period of pregnancy. Ultrasound is measured at 11-13 weeks nuchal translucency, biochemical analysis of blood serum: pregnancy-related test plasma protein A and the chorionic gonadotropin β subunit. | NS | NS | Antenatal care | Screening for fetal anomalies | Screening for aneuploidy |
| **Multiple Pregnancy** | Lithuanian Society of Obstetricians and Gynaecologists, Lithuanian Midwives Association | 2014 | 5.7.2 | If the test was not performed during the first period of pregnancy, offer the woman a second serum analysis at 15 to 20 weeks: the study of alpha fetoprotein, total chorionic gonadotropin, free estriol. | NS | NS | Antenatal care | Screening for fetal anomalies | Screening for aneuploidy |
| **Multiple Pregnancy** | Lithuanian Society of Obstetricians and Gynaecologists, Lithuanian Midwives Association | 2014 | 5.8 and 5.8.1 | Examination for pathology of the fetus: perform ultrasound at 18-20 weeks. | NS | NS | Antenatal care | Screening for fetal anomalies | 2nd Trimester anomaly scan |
| **Role of ultrasonography in the management of twin gestation** | International Federation of Gynecology and Obstetrics (FIGO) | 2018 | NS | Aneuploidy screening should be offered in twin gestations just as in singleton gestations. | NS | NS | Diamniotic/dichorionic twins | Screening for fetal anomalies | Screening for aneuploidy |
| **Role of ultrasonography in the management of twin gestation** | International Federation of Gynecology and Obstetrics (FIGO) | 2018 | NS | Cell-free DNA screening is also available for twin gestations. | NS | NS | Diamniotic/dichorionic twins | Screening for fetal anomalies | Screening for aneuploidy |
| **Role of ultrasonography in the management of twin gestation** | International Federation of Gynecology and Obstetrics (FIGO) | 2018 | NS | Routine anatomic evaluation should be performed at the same gestational age applied to singletons. Dichorionic twins have a slightly higher rate of fetal anomalies (3%) as compared with singletons (2%). | NS | NS | Diamniotic/dichorionic twins | Screening for fetal anomalies | 2nd Trimester anomaly scan |

**Article Title:** Clinical practice guidelines for the antenatal management of dichorionic diamniotic twin pregnancies: a systematic review.

**Author names:**

Caroline O’Connor^1, 2*^, Emily O’Connor^1, 2, 3^, Sara Leitao^2, 3^, Shauna Barrett^4^, Keelin O’Donoghue^1, 2^

**Affiliations**

^1^ INFANT Research Centre, University College Cork, Cork, Ireland

^2^ Pregnancy Loss Research Group, Department of Obstetrics & Gynecology, University College Cork, Cork, Ireland

^3^ National Perinatal Epidemiology Center (NPEC), University College Cork, Cork, Ireland

^4^ Cork University Hospital Library, Cork University Hospital, Cork, Ireland

**Corresponding author:** *Caroline O’Connor

E-mail: carolineoconnor@ucc.ie
