# Supplementary material for: Clinical practice guidelines for the antenatal management of dichorionic diamniotic twin pregnancies: a systematic review
Source: BMC Pregnancy Childbirth. 2023 May 13;23:347. doi: 10.1186/s12884-023-05652-z (PMC10182673; doi:10.1186/s12884-023-05652-z)
Supplement: Supplementary file 7 — Additional file 7 [file 12884_2023_5652_MOESM7_ESM.docx]

| **Guideline title** | **Author** | **Year** | **Recommendation No.** | **Recommendation** | **Strength of Recommendation** | **Quality of evidence** | **Recommendation category specified within guideline** | **Category** | **Subcategory** |
| --- | --- | --- | --- | --- | --- | --- | --- | --- | --- |
| Multifetal Gestations: Twin, Triplet, and Higher-Order Multifetal Pregnancies (Practice Bulletin No 231). | ACOG | 2021 | NS | Multifetal  gestation is considered a high risk factor for preeclampsia. Therefore, low dose aspirin (81 mg/day) prophylaxis is recommended and should be initiated between 12 and 28 weeks of gestation (optimally before 16 weeks of gestation) and continued daily until delivery | NS | NS | Maternal morbidity and mortality | Antenatal care | General care |
| Clinical practice guideline: Management of multiple pregnancy | HSE | 2012 | NS | Antenatal and perinatal care of multiple pregnancies should be hospital-based, delivered by a team that includes obstetricians, ultrasonographers, neonatologists and midwives, all experienced in the management of multiple gestation. A clear pathway should be in place for referral in the case of suspected complications. | NS | NS | Delivery of antenatal and perinatal care | Antenatal care | Multidisciplinary team |
| Twin and Triplet Pregnancy: NG137 | NICE | 2019 | 1.1.13 | ﻿Networks should agree care pathways for managing all twin and triplet pregnancies to ensure that each woman has a care plan in place that is appropriate for the chorionicity and amnionicity of her pregnancy | NS | NS | Chorionicity and amnionicity | Antenatal care | General care |
| Twin and Triplet Pregnancy: NG137 | NICE | 2019 | 1.2.2 | Give women with a twin or triplet pregnancy the same advice about diet, lifestyle and nutritional supplements as in routine antenatal care. | NS | NS | ﻿Diet, lifestyle and nutritional supplements | Antenatal care | General care |
| Twin and Triplet Pregnancy: NG137 | NICE | 2019 | 1.2.3 | ﻿Be aware of the higher incidence of anaemia in women with a twin or triplet pregnancy compared with women with a singleton pregnancy | NS | NS | ﻿Diet, lifestyle and nutritional supplements | Antenatal care | General care |
| Twin and Triplet Pregnancy: NG137 | NICE | 2019 | 1.2.4 | ﻿Perform a full blood count at 20 to 24 weeks to identify women with a twin or triplet pregnancy who need early supplementation with iron or folic acid (this is in addition to the test for anaemia at the routine booking appointment recommended in NICE's guideline on antenatal care for uncomplicated pregnancies). Repeat at 28 weeks as in routine antenatal care | NS | NS | ﻿Diet, lifestyle and nutritional supplements | Antenatal care | General care |
| Twin and Triplet Pregnancy: NG137 | NICE | 2019 | 1.3.1 | Antenatal clinical care for women with a twin or triplet pregnancy should be provided by a nominated multidisciplinary team consisting of: • a core team of named specialist obstetricians, specialist midwives and sonographers, all of whom have experience and knowledge of managing twin and triplet pregnancies • an enhanced team for referrals, which should include: － a perinatal mental health professional － a women's health physiotherapist － an infant feeding specialist • a dietitian. | NS | NS | Antenatal care | Antenatal care | Multidisciplinary team |
| Twin and Triplet Pregnancy: NG137 | NICE | 2019 | 1.3.2 | ﻿Members of the enhanced team should have experience and knowledge relevant to twin and triplet pregnancies. | NS | NS | Antenatal care | Antenatal care | Multidisciplinary team |
| Twin and Triplet Pregnancy: NG137 | NICE | 2019 | 1.3.3 | ﻿Do not routinely refer all women with a twin or triplet pregnancy to the enhanced team but base the decision to refer on each woman's needs. | NS | NS | Antenatal care | Antenatal care | Multidisciplinary team |
| Twin and Triplet Pregnancy: NG137 | NICE | 2019 | 1.3.4 | ﻿Coordinate clinical care for women with a twin or triplet pregnancy to: • minimise the number of hospital visits • provide care as close to the woman's home as possible • provide continuity of care within and between hospitals and the community. | NS | NS | Antenatal care | Antenatal care | location |
| Twin and Triplet Pregnancy: NG137 | NICE | 2019 | 1.3.5 | ﻿The core team should offer information and emotional support specific to twin and triplet pregnancies at their first contact with the woman and provide ongoing opportunities for further discussion and advice including: • antenatal and postnatal mental health and wellbeing • antenatal nutrition (see the recommendation on giving advice in the section on diet, lifestyle and nutritional supplements) • the risks, symptoms and signs of preterm labour and the potential need for corticosteroids for fetal lung maturation • likely timing of birth (see the section on timing of birth) and possible modes of birth (see the section on mode of birth) • breastfeeding • parenting. | NS | NS | Antenatal care | Antenatal care | Multidisciplinary team |
| Twin and Triplet Pregnancy: NG137 | NICE | 2019 | 1.3.7 | ﻿Offer women with an uncomplicated dichorionic diamniotic twin pregnancy at least 8 antenatal appointments with a healthcare professional from the core team. At least 2 of these appointments should be with the specialist obstetrician. • Combine appointments with scans when crown–rump length measures from 45.0 mm to 84.0 mm (at approximately 11+2 weeks to 14+1 weeks) and then at estimated gestations of 20, 24, 28, 32 and 36 weeks. • Offer additional appointments without scans at 16 and 34 weeks. | NS | NS | Schedule of specialist antenatal appointments DCDA | Antenatal care | Multidisciplinary team |
| Twin and Triplet Pregnancy: NG137 | NICE | 2019 | 1.4.13 | ﻿Explain to women and their family members or carers (as appropriate) that: • they have a higher risk of spontaneous preterm birth (see the section on timing of birth) than women with a singleton pregnancy and • this risk is further increased if they have other risk factors, such as a spontaneous preterm birth in a previous pregnancy. | NS | NS | Screening for preterm birth | Antenatal care | Patient education |
| Twin and Triplet Pregnancy: NG137 | NICE | 2019 | 1.4.17 | ﻿Do not use abdominal palpation or symphysis–fundal height measurements to monitor for fetal growth restriction in a dichorionic twin or trichorionic triplet pregnancy. | NS | NS | ﻿Diagnostic monitoring for fetal growth restriction in dichorionic twin and trichorionic triplet pregnancies | Antenatal care | General care |
| Twin and Triplet Pregnancy: NG137 | NICE | 2019 | 1.5.3 | Inform women with a twin or triplet pregnancy of their increased risk of preterm birth (see the recommendation explaining screening for preterm birth to women and their family members in the section on screening for preterm birth) and about the benefits of targeted corticosteroids. | NS | NS | Corticosteroids | Antenatal care | Patient education |
| Twin and Triplet Pregnancy: NG137 | NICE | 2019 | 1.6.1 | ﻿Measure blood pressure and test urine for proteinuria to screen for hypertensive disorders at each antenatal appointment in a twin and triplet pregnancy in line with NICE's guideline on antenatal care for uncomplicated pregnancies. | NS | NS | Maternal complications: Hypertension | Antenatal care | General care |
| Twin and Triplet Pregnancy: NG137 | NICE | 2019 | 1.6.2 | ﻿Advise women with a twin or triplet pregnancy to take low-dose aspirin daily from 12 weeks until the birth of the babies if they have 2 or more of the risk factors specified in NICE's guideline on hypertension in pregnancy. Specify what these are? | NS | NS | Maternal complications: Hypertension | Antenatal care | General care |
| Twin and Triplet Pregnancy: NG137 | NICE | 2019 | 1.7.1 | ﻿Seek a consultant opinion from a tertiary level fetal medicine centre for:  • pregnancies complicated by any of the following: － fetal weight discordance (of 25% or more) and an EFW of any of the babies below the 10th centile for gestational age－ fetal anomaly (structural or chromosomal) － discordant fetal death | NS | NS | ﻿Indications for referral to a tertiary level fetal medicine centre | Antenatal care | Location |
| Twin and Triplet Pregnancy: NG137 | NICE | 2019 | 1.8.1 | ﻿From 24 weeks in a twin or triplet pregnancy, discuss with the woman (and her family members or carers, as appropriate) her plans and wishes for the birth of her babies. Provide information that is tailored to each woman's pregnancy, taking into account her needs and preferences. Revisit these conversations whenever clinically indicated and whenever the woman wants to. | NS | NS | Planning birth: information and support | Antenatal care | Patient education |
| Twin and Triplet Pregnancy: NG137 | NICE | 2019 | 1.8.2 | ﻿Ensure the following has been discussed by 28 weeks at the latest: ﻿• place of birth and the possible need to transfer in case of preterm birth • timing and possible modes of birth • analgesia during labour (or for caesarean birth) • intrapartum fetal heart monitoring • management of the third stage of labour. | NS | NS | Planning birth: information and support | Antenatal care | Patient education |
| Twin and Triplet Pregnancy: NG137 | NICE | 2019 | 1.8.3 | ﻿Follow NICE's guideline on patient experience in adult NHS services for how to provide information and communicate with women and their families and carers. | NS | NS | Planning birth: information and support | Antenatal care | Patient education |
| Twin and Triplet Pregnancy: NG137 | NICE | 2019 | 1.9.1 | ﻿Explain to women with a twin pregnancy that about 60 in 100 twin pregnancies result in spontaneous birth before 37 weeks. | NS | NS | Timing of birth | Antenatal care | Patient education |
| Twin and Triplet Pregnancy: NG137 | NICE | 2019 | 1.9.3 | ﻿Explain to women with a twin or triplet pregnancy that spontaneous preterm birth and planned preterm birth are associated with an increased risk of admission to a neonatal unit. | NS | NS | Timing of birth | Antenatal care | Patient education |
| Twin and Triplet Pregnancy: NG137 | NICE | 2019 | 1.9.4 | ﻿Explain to women with an uncomplicated dichorionic diamniotic twin pregnancy that:  • planned birth from 37+0 weeks does not appear to be associated with an increased risk of serious neonatal adverse outcomes and • continuing the pregnancy beyond 37+6 weeks increases the risk of fetal death. | NS | NS | Timing of birth | Antenatal care | Patient education |
| Twin and Triplet Pregnancy: NG137 | NICE | 2019 | 1.10.1 | ﻿Explain to women with an uncomplicated twin pregnancy planning their mode of birth that planned vaginal birth and planned caesarean section are both safe choices for them and their babies if all of the following apply: • the pregnancy remains uncomplicated and has progressed beyond 32 weeks • there are no obstetric contraindications to labour • the first baby is in a cephalic (head-first) presentation ﻿and •there is no significant size discordance between the twins. | NS | NS | Mode of birth: DCDA | Antenatal care | Patient education |
| Twin and Triplet Pregnancy: NG137 | NICE | 2019 | 1.10.2 | ﻿Explain to women with an uncomplicated twin pregnancy that for women giving birth after 32 weeks (see recommendation 1.10.1): • more than a third of women who plan a vaginal birth go on to have a caesarean section • almost all women who plan a caesarean section do have one, but a few women have a vaginal birth before caesarean section can be carried out • a small number of women who plan a vaginal birth will need an emergency caesarean section to deliver the second twin after vaginal birth of the first twin. | NS | NS | Mode of birth: DCDA | Antenatal care | Patient education |
| Twin pregnancy | South Australian Perinatal Practice Guideline | 2018 | NS | Refer women with multiple pregnancies to additional support services. | NS | NS | Summary of Practice Recommendations | Antenatal care | Multidisciplinary team |
| Twin pregnancy | South Australian Perinatal Practice Guideline | 2018 | NS | Refer women with multiple pregnancies to a level 4 or higher maternity unit if she begins care in a lower level unit. | NS | NS | Summary of Practice Recommendations | Antenatal care | Location of care |
| Twin pregnancy | South Australian Perinatal Practice Guideline | 2018 | NS | Women should be informed of the increased risks associated with twin pregnancy. | NS | NS | Summary of Practice Recommendations | Antenatal care | Patient education |
| Twin pregnancy | South Australian Perinatal Practice Guideline | 2018 | NS | Antenatal visits may need to be more frequent than in singleton pregnancies for the timely detection and treatment of medical or obstetric complications. | NS | NS | Summary of Practice Recommendations | Antenatal care | General care |
| Twin pregnancy | South Australian Perinatal Practice Guideline | 2018 | NS | Encourage women with a multiple pregnancy to attend antenatal education specific to care and management of multiple birth. | NS | NS | Antenatal care in pregnancy | Antenatal care | Patient education |
| Twin pregnancy | South Australian Perinatal Practice Guideline | 2018 | NS | Encourage women with a multiple pregnancy to join the South Australian Multiple Birth Association | NS | NS | Antenatal care in pregnancy | Antenatal care | Organisation |
| Twin pregnancy | South Australian Perinatal Practice Guideline | 2018 | NS | Hospitalisation may be appropriate for specific pregnancy complications. | NS | NS | Subsequent care in pregnancy | Antenatal Care | General care |
| Twin pregnancy | South Australian Perinatal Practice Guideline | 2018 | NS | Nutritional advice is recommended and may include supplementary iron and folate to accommodate the increased needs in twin pregnancies. | NS | NS | Subsequent care in pregnancy | Antenatal Care | General care |
| Twin pregnancy | South Australian Perinatal Practice Guideline | 2018 | NS | Recommend the avoidance of strenuous work in the second half of pregnancy. | NS | NS | Subsequent care in pregnancy | Antenatal Care | General care |
| Twin pregnancy | South Australian Perinatal Practice Guideline | 2018 | NS | Antenatal visits may need to be more frequent than in singleton pregnancies for the timely detection and treatment of medical or obstetric complications. | NS | NS | Subsequent care in pregnancy | Antenatal Care | General care |
| Twin pregnancy | South Australian Perinatal Practice Guideline | 2018 | NS | Anti-D prophylaxis 625 IU is recommended at 28 and 34 weeks for all Rh negative women | NS | NS | Subsequent care in pregnancy | Antenatal Care | General care |
| FIGO Good clinical practice advice: management of twin pregnancy | FIGO | 2019 | NS | Women should be cared for by multidisciplinary team in specialised twin clinic. | NS | NS | Where should antenatal care take place? | Antenatal care | Multidisciplinary team |
| FIGO Good clinical practice advice: management of twin pregnancy | FIGO | 2019 | NS | Twin pregnancies that develop complications should be referred to tertiary centre | NS | NS | Where should antenatal care take place? | Antenatal care | Multidisciplinary team |
| FIGO Good clinical practice advice: management of twin pregnancy | FIGO | 2019 | NS | Pregnant women with twins should be counselled about increased risks of twin pregnancy | NS | NS | Where should antenatal care take place? | Antenatal care | Patient education |
| FIGO Good clinical practice advice: management of twin pregnancy | FIGO | 2019 | NS | Check Hb at 20-24 weeks and at 28-34 weeks. | NS | NS | Where should antenatal care take place? | Antenatal care | General care |
| FIGO Good clinical practice advice: management of twin pregnancy | FIGO | 2019 | NS | Prescribe aspirin 75mg daily for women with other risk factors for pre-eclampsia from 12 weeks | NS | NS | Where should antenatal care take place? | Antenatal care | General care |
| Tvillinger - ﻿håndtering af graviditet og fødsel (twins- handling pregnancy and childbirth) | Sandbjerg | 2010 | NS | Follow-up, treatment and delivery of twin pregnant women with pregnancy-related hypertension, preeclampsia, gestational cholestasis and gestational diabetes follow recommendations for singletons. | C | NS | Recommendations with strengths | Antenatal care | General care |
| Tvillinger - ﻿håndtering af graviditet og fødsel (twins- handling pregnancy and childbirth) | Sandbjerg | 2010 | NS | Given the increased incidence of gestational diabetes mellitus in twin pregnant women, one may consider oral glucose load test also for twin pregnant women who have no other risk factors. | C | 3 | Recommendations with strengths | Antenatal care | General care |
| Tvillinger - ﻿håndtering af graviditet og fødsel (twins- handling pregnancy and childbirth) | Sandbjerg | 2010 | NS | The increased incidence of postpartum depression in twin mothers causes that of twin mothers requires extra attention both pre- and postpartum | C | NS | Recommendations with strengths | Antenatal care | General care |
| Tvillinger - ﻿håndtering af graviditet og fødsel (twins- handling pregnancy and childbirth) | Sandbjerg | 2010 | NS | Antihypertensive treatment in pregnancy is also initiated to protect the pregnant woman against intracranial haemorrhage. There is no reason to assume that hypertension in twin pregnancies has a less negative effect on the pregnant woman's health than for singleton pregnancies. Therefore, it is recommended that hypertension treatment in twin pregnant women follow the same guidelines as for singleton pregnant women (http://www.dsog.dk/sandbjerg/Hypertension-Preeklampsi.pdf). The recommendation within the hypertension and preeclampsia guideline is Tbl Magnyl (aspirin) 150 mg daily taken at night time is recommended to be started early in pregnancy; Possibly in connection with the first pregnancy control in electricity and preferably before the 16th week of pregnancy with discontinuation at GA 37 + 0 to the following risk groups: • Severe preeclampsia in previous pregnancies (level of evidence A) • Chronic kidney disease (level of evidence A) • Autoimmune disease such as SLE or antiphospholipid syndrome (level of evidence B) • Pre gestational diabetes type 1 or 2 (level of evidence B) • Essential hypertension requiring treatment (level of evidence B) • Eggs donated (level of evidence B) • Multiple pregnancies• BMI> 30 | B | 4 | Pregnancy related hypertension and Prophylaxis for risk groups (HTN and PET guideline) | Antenatal care | General care |
| Management of multiple pregnancy | SIGO, AOGOI, AGUI | 2016 | NS | In the case of a multiple pregnancy that is non-complicated it is important to carry out, on occasion of the first evaluation, an appropriate counselling in terms of related complications and the methods of surveillance. | B | 6 | Clinical management of uncomplicated multiple pregnancy | Antenatal care | Patient education |
| Management of multiple pregnancy | SIGO, AOGOI, AGUI | 2016 | NS | During pregnancy, it is indicated to carry of the glucose load curve (GTT). | C | 4 | Clinical management of uncomplicated multiple pregnancy | Antenatal care | General care |
| Management of multiple pregnancy | SIGO, AOGOI, AGUI | 2016 | NS | The antepartum cardiotocographic monitoring in uncomplicated DCDA pregnancies is not indicated | D | 6 | Clinical management of uncomplicated multiple pregnancy | Antenatal care | General care |
| Management of multiple pregnancy | SIGO, AOGOI, AGUI | 2016 | NS | Indications for sending a multiple pregnancy to a tertiary/specialist centre: MCMA in twins/triplets, MCDA, triplets, DCDA complicated by one or more of discordant growth, fetal anomalies, stillbirth of a twin, and multiple pregnancies identified as high risk for down syndrome. | NS | NS | Clinical management of uncomplicated multiple pregnancy | Antenatal care | Location |
| Twin pregnancies: guidelines for clinical practice from the French College of Gynaecologists and Obstetricians (CNGOF) | Christophe Vayssiere | 2011 | NS | Monthly follow-up by a gynaecologist–obstetrician in an appropriate facility is recommended (Professional Consensus). | NS | NS | Professional consensus | Antenatal Care | Multidisciplinary |
| Twin pregnancies: guidelines for clinical practice from the French College of Gynaecologists and Obstetricians (CNGOF) | Christophe Vayssiere | 2011 | NS | For mothers with a pre-pregnancy body mass index (BMI) between 19 and 25, the total recommended weight gain is 16–24 kg (Level B) | NS | NS | Level B | Antenatal Care | General care |
| Twin pregnancies: guidelines for clinical practice from the French College of Gynaecologists and Obstetricians (CNGOF) | Christophe Vayssiere | 2011 | NS | Current data are insufficient to justify a recommendation for or against systematic screening for pregnancy-related diabetes (Professional Consensus). | NS | NS | Professional consensus | Antenatal Care | General care |
| Twin pregnancies: guidelines for clinical practice from the French College of Gynaecologists and Obstetricians (CNGOF) | Christophe Vayssiere | 2011 | NS | Because of the increased risk of obstetrical complications, more intensive follow-up may be set up during the third trimester (Professional Consensus) | NS | NS | Professional consensus | Antenatal care | General care |
| Twin pregnancies: guidelines for clinical practice from the French College of Gynaecologists and Obstetricians (CNGOF) | Christophe Vayssiere | 2011 | NS | Prenatal care of twin pregnancies is not currently well codified in France. It must be performed by a physician with good knowledge of this type of pregnancy (Professional Consensus). | NS | NS | Professional consensus | Antenatal care | Multidisciplinary |
| Twin pregnancies: guidelines for clinical practice from the French College of Gynaecologists and Obstetricians (CNGOF) | Christophe Vayssiere | 2011 | NS | Current data are insufficient to justify a recommendation that ‘‘twin clinics’’ be set up in France for the management of twin pregnancies (Professional Consensus) | NS | NS | Professional consensus | Antenatal care | Location of care |
| Twin pregnancies: guidelines for clinical practice from the French College of Gynaecologists and Obstetricians (CNGOF) | Christophe Vayssiere | 2011 | NS | The increased risk of maternal complications and the high rate of medical interventions (caesareans, instrumental operative intervention, and manoeuvres) justify the immediate and permanent availability of a gynaecologist–obstetrician with experience in the vaginal delivery of twins (Professional Consensus). | NS | NS | Professional consensus | Antenatal care | Multidisciplinary |
| Twin pregnancies: guidelines for clinical practice from the French College of Gynaecologists and Obstetricians (CNGOF) | Christophe Vayssiere | 2011 | NS | The immediate and permanent availability of a paediatric team appropriate in size and resuscitation skills to the number of new-borns and the extent of their prematurity is recommended. (Professional Consensus). Twins may be delivered in maternity units that meet these specifications (Professional Consensus) | NS | NS | Professional consensus | Antenatal care | Location of care |
| Twin pregnancies: guidelines for clinical practice from the French College of Gynaecologists and Obstetricians (CNGOF) | Christophe Vayssiere | 2011 | NS | The patient should receive thorough information about the risks of vaginal and caesarean deliveries (Professional Consensus). | NS | NS | Professional consensus | Antenatal care | Patient education |
| Multiple Pregnancy | Lithuanian Society of Obstetricians and Gynaecologists, Lithuanian Midwives Association | 2014 | 5.1 | A pregnant woman who has been diagnosed with a multiple pregnancy should be supervised by an obstetrician-gynaecologist | NS | NS | Antenatal care | Antenatal care | Multidisciplinary team |
| Multiple Pregnancy | Lithuanian Society of Obstetricians and Gynaecologists, Lithuanian Midwives Association | 2014 | 5.2 | The pregnant woman should be informed of the possible complications of pregnancy and childbirth and explain the importance of more frequent doctor visits. | NS | NS | Antenatal care | Antenatal care | Patient education |
| Multiple Pregnancy | Lithuanian Society of Obstetricians and Gynaecologists, Lithuanian Midwives Association | 2014 | 5.3 | Recommended for pregnant women: Eat foods that are higher in protein, Reduce exercise, lie down more, especially in the second half of pregnancy. Weight gain is recommended | NS | NS | Antenatal care | Antenatal care | General care |
| Multiple Pregnancy | Lithuanian Society of Obstetricians and Gynaecologists, Lithuanian Midwives Association | 2014 | 5.9.4 | In the case of multiple pregnancies, fundal height in terms of fetal growth in is not measured | NS | NS | Antenatal care | Antenatal care | General care |
| Multiple Pregnancy | Lithuanian Society of Obstetricians and Gynaecologists, Lithuanian Midwives Association | 2014 | 5.13.1 | Gestational diabetes: Gestational diabetes is more common (3-6%) than in multiple pregnancies in the case of single fetus and even more in the case of yolks (22-39%). In the case of multiple pregnancies 24-28 weeks is recommended to check for gestational diabetes. Under the supervision of a multifetal pregnancy, refer to the guidelines for the diagnosis and treatment of gestational diabetes for a single pregnancy. | NS | NS | Antenatal care | Antenatal care | General care |
| Multiple Pregnancy | Lithuanian Society of Obstetricians and Gynaecologists, Lithuanian Midwives Association | 2014 | 5.14.4 | During multiple pregnancies, it is recommended that 75 mg of aspirin be taken daily from 12 weeks of gestation before delivery if the pregnant woman has one of the following risk factors for hypertension:● first pregnancy;● 40 years or more;● last pregnancy 10 years or more;● BMI ≥ 35;● family history of preeclampsia | NS | NS | Antenatal care | Antenatal care | General care |

**Article Title:** Clinical practice guidelines for the antenatal management of dichorionic diamniotic twin pregnancies: a systematic review.

**Author names:**

Caroline O’Connor^1, 2*^, Emily O’Connor^1, 2, 3^, Sara Leitao^2, 3^, Shauna Barrett^4^, Keelin O’Donoghue^1, 2^

**Affiliations**

^1^ INFANT Research Centre, University College Cork, Cork, Ireland

^2^ Pregnancy Loss Research Group, Department of Obstetrics & Gynecology, University College Cork, Cork, Ireland

^3^ National Perinatal Epidemiology Center (NPEC), University College Cork, Cork, Ireland

^4^ Cork University Hospital Library, Cork University Hospital, Cork, Ireland

**Corresponding author:** *Caroline O’Connor

E-mail: carolineoconnor@ucc.ie
