# Supplementary material for: Clinical practice guidelines for the antenatal management of dichorionic diamniotic twin pregnancies: a systematic review
Source: BMC Pregnancy Childbirth. 2023 May 13;23:347. doi: 10.1186/s12884-023-05652-z (PMC10182673; doi:10.1186/s12884-023-05652-z)
Supplement: Supplementary file 8 — Additional file 8 [file 12884_2023_5652_MOESM8_ESM.docx]

| **Guideline title** | **Author** | **Year** | **Recommendation No.** | **Recommendation** | **Strength of Recommendation** | **Quality of evidence** | **Recommendation category specified within guideline** | **Category** | **Subcategory** |
| --- | --- | --- | --- | --- | --- | --- | --- | --- | --- |
| **Multifetal Gestations: Twin, Triplet, and Higher-Order Multifetal Pregnancies (Practice Bulletin No 231).** | ACOG | 2021 | NS | There are no interventions that have been shown to prevent spontaneous preterm delivery in asymptomatic women with multifetal gestations identified to be at risk based on these screening methods (including transvaginal ultrasonographic cervical length, digital examination, fetal fibronectin screening, and home uterine monitoring). The routine use of these screening methods in asymptomatic women with multifetal pregnancies is not recommended. | NS | NS | Asymptomatic women | Preterm labour | Screening |
| **Multifetal Gestations: Twin, Triplet, and Higher-Order Multifetal Pregnancies (Practice Bulletin No 231).** | ACOG | 2021 | NS | (spontaneous preterm birth)In symptomatic women, the positive predictive value of a fetal fibronectin test result or of a short cervical length alone is poor, and they should not be used exclusively to direct management in the setting of acute symptoms | NS | NS | Symptomatic women | Preterm labour | Screening |
| **Multifetal Gestations: Twin, Triplet, and Higher-Order Multifetal Pregnancies (Practice Bulletin No 231).** | ACOG | 2021 | NS | Routine prophylactic interventions including cerclage, hospitalization, bedrest, tocolytics, and pessary have not been proved to decrease neonatal morbidity or mortality, and therefore should not be used based solely on the indication of multifetal gestation | NS | NS | Routine prophylactic interventions | Preterm labour | Interventions |
| **Multifetal Gestations: Twin, Triplet, and Higher-Order Multifetal Pregnancies (Practice Bulletin No 231).** | ACOG | 2021 | NS | Prophylactic cerclage placement in women with a twin gestation or a triplet gestation without a history of cervical insufficiency has not been shown to be beneficial. | Level B | NS | Prophylactic cerclage | Preterm labour | Interventions |
| **Multifetal Gestations: Twin, Triplet, and Higher-Order Multifetal Pregnancies (Practice Bulletin No 231).** | ACOG | 2021 | NS | Bedrest with or without hospitalization in women with multi-fetal pregnancies is not recommended because of the lack of benefit and the risk of thrombosis and deconditioning associated with prolonged bed rest in pregnancy. | Level B | NS | Routine hospitilization and bed rest | Preterm labour | Interventions |
| **Multifetal Gestations: Twin, Triplet, and Higher-Order Multifetal Pregnancies (Practice Bulletin No 231).** | ACOG | 2021 | NS | There is no role for the prophylactic use of any tocolytic agent in women with multifetal gestations, including the prolonged use of betamimetics for this indication. | Level A | NS | Prophylactic tocolytics | Preterm labour | Interventions |
| **Multifetal Gestations: Twin, Triplet, and Higher-Order Multifetal Pregnancies (Practice Bulletin No 231).** | ACOG | 2021 | NS | Based on available evidence, the use of prophylactic cervical pessary is not recommended in multifetal pregnancies. | Level B | NS | Prophylactic pessary | Preterm labour | Interventions |
| **Multifetal Gestations: Twin, Triplet, and Higher-Order Multifetal Pregnancies (Practice Bulletin No 231).** | ACOG | 2021 | NS | Progesterone treatment does not reduce the incidence of spontaneous preterm birth in unselected women with twin or triplet gestations and, therefore, is not recommended | Level A | NS | Progesterone treatment | Preterm labour | Interventions |
| **Multifetal Gestations: Twin, Triplet, and Higher-Order Multifetal Pregnancies (Practice Bulletin No 231).** | ACOG | 2021 | NS | In multifetal gestations a brief course of tocolysis may be considered for up to 48 hours in the setting of acute preterm labor, in order to allow corticosteroids to be administered. | NS | NS | Tocolytics | Preterm labour | Interventions |
| **Multifetal Gestations: Twin, Triplet, and Higher-Order Multifetal Pregnancies (Practice Bulletin No 231).** | ACOG | 2021 | NS | Unless a contraindication exists, a course of antenatal corticosteroids should be administered to all patients who are at risk of delivery within 7 days and who are between 24 and 34 weeks gestation, irrespective of the fetal number. | Level B | NS | Corticosteroids | Preterm labour | Interventions |
| **Multifetal Gestations: Twin, Triplet, and Higher-Order Multifetal Pregnancies (Practice Bulletin No 231).** | ACOG | 2021 | NS | A single repeat course of antenatal corticosteroids should be considered in women with a gestation of less than 34 weeks, who have an imminent risk of preterm delivery within the next 7 days, and whose prior course of antenatal corticosteroids was administered more than 14 days previously. Rescue-course corticosteroids could be provided as early as 7 days from the prior dose, if indicated by the clinical scenario. | NS | NS |  | Preterm labour | Interventions |
| **Multifetal Gestations: Twin, Triplet, and Higher-Order Multifetal Pregnancies (Practice Bulletin No 231).** | ACOG | 2021 | NS | Magnesium sulfate reduces the severity and risk of cerebral palsy in surviving infants if administered when birth is anticipated before 32 weeks of gestation, regardless of fetal number. | Level B | NS | Magnesium sulphate for fetal neuroprotection | Preterm labour | Interventions |
| **ISUOG Practice Guidelines: role of ultrasound in twin pregnancy** | ISUOG | 2016 | NS | Cervical length assessment is performed ideally at the same visit as the anomaly scan in the second trimester, in order to identify women at risk | 2+ | 2++ |  | Preterm labour | Interventions |
| **ISUOG Practice Guidelines: role of ultrasound in twin pregnancy** | ISUOG | 2016 | NS | ﻿Cervical length measurement is the preferred method of screening for preterm birth in twins; 25mm is the cut-off most commonly used in the second trimester | B | 2++ | ﻿Screening for risk of preterm birth in twin pregnancy. | Preterm labour | Screening |
| **Ultrasound in twin pregnancies: SOGC Clinical practice guideline No. 260** | SOGC | 2011 | 9 | ﻿When ultrasound is used to screen for preterm birth in a twin gestation, endovaginal ultrasound measurement of the cervical length should be performed. | A | II-2 | Screening for preterm birth | Preterm labour | Screening |
| **Ultrasound in twin pregnancies: SOGC Clinical practice guideline No. 260** | SOGC | 2011 | Summary statement 2 | ﻿There are insufficient data to recommend a routine preterm labour surveillance protocol in terms of frequency, timing, and optimal cervical length thresholds. | NS | II-2 | Screening for preterm birth | Preterm labour | Screening |
| **Clinical practice guideline: Management of multiple pregnancy** | HSE | 2012 | NS | Although mid-trimester determination of cervical length may predict preterm birth in twins, such evaluation is not recommended, on the basis that there is no preventative intervention that has been shown to reduce the risk of spontaneous preterm twin birth. Specifically, there is no evidence to support bed rest, elective placement of cervical cerclage, ultrasound-indicated cerclage, prophylactic tocolytic therapy or progesterone for the purpose of prevention of preterm twin birth | NS | NS | Preterm delivery | Preterm labour | Screening |
| **Clinical practice guideline: Management of multiple pregnancy** | HSE | 2012 | NS | A course of antenatal corticosteroid therapy should be prescribed in the event of anticipated preterm delivery prior to 34 weeks gestation, accepting that the benefit of antenatal corticosteroids in the prevention of respiratory morbidity may be less than that observed in singleton pregnancies. Corticosteroid therapy should be administered in a ‘timed’ rather than in an untargeted (‘routine’) manner. | NS | NS | Preterm delivery | Preterm labour | Interventions |
| **Clinical practice guideline: Management of multiple pregnancy** | HSE | 2012 | NS | Applying a strategy of close fetal surveillance, perinatal morbidity can be minimised by allowing dichorionic twins to 38 weeks. If elective preterm delivery is offered prior to these suggested gestational ages, a timed course of antenatal corticosteroids should be administered. | NS | NS | Timing of delivery | Preterm labour | Interventions |
| **Twin and Triplet Pregnancy: NG137** | NICE | 2019 | 1.4.14 | ﻿Do not use fetal fibronectin testing alone to predict the risk of spontaneous preterm birth in twin and triplet pregnancy. | NS | NS | Screening for preterm birth | Preterm labour | Screening |
| **Twin and Triplet Pregnancy: NG137** | NICE | 2019 | 1.4.15 | ﻿Do not use home uterine activity monitoring to predict the risk of spontaneous preterm birth in twin and triplet pregnancy. | NS | NS | Screening for preterm birth | Preterm labour | Screening |
| **Twin and Triplet Pregnancy: NG137** | NICE | 2019 | 1.5.1 | ﻿Do not offer intramuscular progesterone to prevent spontaneous preterm birth in women with a twin or triplet pregnancy. | NS | NS | Preventing preterm birth | Preterm labour | Interventions |
| **Twin and Triplet Pregnancy: NG137** | NICE | 2019 | 1.5.2 | ﻿Do not offer the following interventions (alone or in combination) routinely to prevent spontaneous preterm birth in women with a twin or triplet pregnancy: • arabin pessary • bed rest • cervical cerclage • oral tocolytics. | NS | NS | Preventing preterm birth | Preterm labour | Interventions |
| **Twin and Triplet Pregnancy: NG137** | NICE | 2019 | 1.5.4 | Do not use single or multiple untargeted (routine) courses of corticosteroids in twin or triplet pregnancy. Inform women that there is no benefit in using untargeted administration of corticosteroids. | NS | NS | Corticosteroids | Preterm labour | Interventions |
| **Twin pregnancy** | South Australian Perinatal Practice Guideline | 2018 | NS | Hospitalisation for bed rest, and prophylactic tocolytics have not been shown to confer advantage and do not reduce the frequency of preterm birth or perinatal death. | NS | NS | Subsequent care in pregnancy | Preterm labour | Interventions |
| **Twin pregnancy** | South Australian Perinatal Practice Guideline | 2018 | NS | Progesterone does not reduce preterm delivery of twins. | NS | NS | Subsequent care in pregnancy | Preterm labour | Interventions |
| **Twin pregnancy** | South Australian Perinatal Practice Guideline | 2018 | NS | The wellbeing of both twins should be ascertained by cardiotocography before tocolytics are considered. | NS | NS | Preterm labour | Preterm labour | Interventions |
| **Twin pregnancy** | South Australian Perinatal Practice Guideline | 2018 | NS | If inhibition of labour is indicated follow the guidelines for tocolysis in preterm labour (see Nifedipine for preterm labour PPG available at www.sahealth.sa.gov.au/perinatal). | NS | NS | Preterm labour | Preterm labour | Interventions |
| **Twin pregnancy** | South Australian Perinatal Practice Guideline | 2018 | NS | Corticosteroids are indicated as in a singleton pregnancy. | NS | NS | Preterm labour | Preterm labour | Interventions |
| **FIGO Good clinical practice advice: management of twin pregnancy** | FIGO | 2019 | NS | In all twin pregnancies, the 20 week anatomy scan should include cervical length measurement to identify women at increased risk of extreme prematurity. | NS | NS | Timing, frequency and content of ultrasound assessment | premature | screening |
| **FIGO Good clinical practice advice: management of twin pregnancy** | FIGO | 2019 | NS | Screening and prevention of preterm birth: Second trimester cervical length screening by TV USS should be offered. | NS | NS | Screening for and prevention of preterm birth | Preterm labour | Screening |
| **FIGO Good clinical practice advice: management of twin pregnancy** | FIGO | 2019 | NS | Screening and prevention of preterm birth: Use a cervical length of 20mm as the threshold for short cervix in asymptomatic women | NS | NS | Screening for and prevention of preterm birth | Preterm labour | Screening |
| **FIGO Good clinical practice advice: management of twin pregnancy** | FIGO | 2019 | NS | Screening and prevention of preterm birth: Steroids should be given if delivery is expected <34 weeks’ or if planned C section <37 weeks. | NS | NS | Screening for and prevention of preterm birth | Preterm labour | Interventions |
| **FIGO Good clinical practice advice: management of twin pregnancy** | FIGO | 2019 | NS | Repeat courses of steroids in case of threatened preterm labour should be based on individual circumstances and not be routine practice. | NS | NS | Screening for and prevention of preterm birth | Preterm labour | Interventions |
| **ACR Appropriateness Criteria: Multiple Gestation** | American College of Radiology | 2017 | NS | Transabdominal US is performed in monochorionic twins for fetal anatomy and to screen for fetal anomalies and TTTS. Fetal echocardiography helps screen for structural congenital cardiac anomalies. Transvaginal US of the cervix may help triage patients into higher-risk group for preterm delivery. Duplex Doppler velocimetry is recommended in cases of TTTS, velamentous cord insertion, and sIUGR. | NS |  | Summary of recommendations | Preterm labour | Screening |
| **AWMF 015-087 S2e Guideline Monitoring and Care of Twin Pregnancies** | AWMF | 2020 | 27 | Ultrasound cervical length measurement is the preferred screening method for preterm birth of twins; ▪ a cervix length of < 25 cm in the second trimester should be used as a cut-off | B | EK, 2++ | Screening for premature birth in twin pregnancies | Preterm labour | Screening |
| **Tvillinger - ﻿håndtering af graviditet og fødsel (twins- handling pregnancy and childbirth)** | Sandbjerg | 2010 | NS | Measurement of the cervical length in women with a twin pregnancy between 22-24 weeks, is suitable for women (with twin pregnancies) who are at low risk of giving birth preterm. Cut off of 25 mm is recommended. | B | NS | Recommendations with strengths | Preterm labour | Screening |
| **Tvillinger - ﻿håndtering af graviditet og fødsel (twins- handling pregnancy and childbirth)** | Sandbjerg | 2010 | NS | Cervical scan at week 23 in twins has less sensitivity, but in those cases where you find a shortened cervix, you can give Celeston and possibly transfer to a ward that can receive extremely premature babies. The method, on the other hand, is good at identifying the twin mothers who have a lot lower risk of giving birth before week 32-33 |  | 2 | Outcome by cervical screening | Preterm labour | Screening |
| **Ultrasound for twin and multiple pregnancies** | Toward optimized practice (TOP) | 2017 | NS | Consider performing endovaginal ultrasound measurement of closed cervical length when ultrasound is used in either screening for risk of preterm birth, or in the assessment of spontaneous preterm labour. Note: there is insufficient data to recommend a routine preterm labour surveillance protocol i.e., frequency, timing, and optimal cervical length thresholds. | NS |  | Second and third trimester studies | Preterm labour | Screening |
| **Management of multiple pregnancy** | SIGO, AOGOI, AGUI | 2016 | NS | Prophylactic cerclage in women with multiple pregnancy should be avoided. | A | 1 | Clinical management of uncomplicated multiple pregnancy | Preterm labour | Interventions |
| **Management of multiple pregnancy** | SIGO, AOGOI, AGUI | 2016 | NS | Prophylactic hospitalization and bed rest are not recommended. | D | 1 | Clinical management of uncomplicated multiple pregnancy | Preterm labour | Interventions |
| **Management of multiple pregnancy** | SIGO, AOGOI, AGUI | 2016 | NS | The use of prophylactic tocolysis is not recommended. | C | 1 | Clinical management of uncomplicated multiple pregnancy | Preterm labour | Interventions |
| **Management of multiple pregnancy** | SIGO, AOGOI, AGUI | 2016 | NS | If preterm delivery is suspected, even in multiple pregnancy, steroids should be administered between 24 and 34 weeks of pregnancy | B | 2 | Clinical management of uncomplicated multiple pregnancy | Preterm labour | Interventions |
| **Management of multiple pregnancy** | SIGO, AOGOI, AGUI | 2016 | NS | The use of the prophylactic pessary in the 2nd trim in patients with a normal cervix and above the 25th centile is not recommended. | A | 1 | Clinical management of uncomplicated multiple pregnancy | Preterm labour | Interventions |
| **Management of multiple pregnancy** | SIGO, AOGOI, AGUI | 2016 | NS | The use of the prophylactic pessary in the 2nd trim in patients with cervical length below the 25th centile, can be an option to recommend. | A | 1 | Clinical management of uncomplicated multiple pregnancy | Preterm labour | Interventions |
| **Management of multiple pregnancy** | SIGO, AOGOI, AGUI | 2016 | NS | Progesterone treatment does not reduce the incidence of spontaneous preterm labour in the unselected population with multiple pregnancy therefore it is not recommended. | D | 1 | Clinical management of uncomplicated multiple pregnancy | Preterm labour | Interventions |
| **Management of multiple pregnancy** | SIGO, AOGOI, AGUI | 2016 | NS | The biochemical test using fetal fibronectin has a role in predicting the preterm labour even in multiple pregnancy but as the role of the therapeutic cerclage and other medical therapies is still under discussion, this method does not find application as a generalized screening method. | C | 4 | Maternal and fetal complications | Preterm labour | Screening |
| **Twin pregnancies: guidelines for clinical practice from the French College of Gynaecologists and Obstetricians (CNGOF)** | Christophe Vayssiere | 2011 | NS | Neither tocography nor screening for bacterial vaginosis allows the identification of a population at risk of preterm delivery |  | NS | Level B and level C | Preterm labour | Screening |
| **Twin pregnancies: guidelines for clinical practice from the French College of Gynaecologists and Obstetricians (CNGOF)** | Christophe Vayssiere | 2011 | NS | Current data in the literature are contradictory and insufficient to determine whether the results of either testing for fetal fibronectin in cervicovaginal secretions or digital cervical examination are predictive of preterm delivery (Professional Consensus). |  | NS | Professional consensus | Preterm labour | Screening |
| **Twin pregnancies: guidelines for clinical practice from the French College of Gynaecologists and Obstetricians (CNGOF)** | Christophe Vayssiere | 2011 | NS | Only obstetric history (history of preterm delivery) (Level C) and especially transvaginal ultrasound measurement of cervical length (Level B) are predictive factors for preterm delivery. |  | NS | Level B and level C | Preterm labour | Screening |
| **Twin pregnancies: guidelines for clinical practice from the French College of Gynaecologists and Obstetricians (CNGOF)** | Christophe Vayssiere | 2011 | NS | No study has shown that the identification by transvaginal ultrasound of a group at risk of preterm delivery makes it possible to reduce the frequency of such deliveries in asymptomatic patients carrying twins (Professional Consensus). |  | NS | Professional consensus | Preterm labour | Screening |
| **Twin pregnancies: guidelines for clinical practice from the French College of Gynaecologists and Obstetricians (CNGOF)** | Christophe Vayssiere | 2011 | NS | Transvaginal ultrasound is performed, information about a long cervix (>30 mm) is more pertinent than that of a shortened cervix (<25 mm) (Professional Consensus |  | NS | Professional consensus | Preterm labour | Screening |
| **Twin pregnancies: guidelines for clinical practice from the French College of Gynaecologists and Obstetricians (CNGOF)** | Christophe Vayssiere | 2011 | NS | Preterm delivery rates have not been reduced by any of the following interventions: strict bedrest, use of prophylactic oral tocolytics, administration of progesterone, or prophylactic cerclage in patients with or without cervical modifications (Level A) |  | NS | Level A | Preterm labour | Interventions |
| **Multiple Pregnancy** | Lithuanian Society of Obstetricians and Gynaecologists, Lithuanian Midwives Association | 2014 | 5.15.2 | Measurement of cervical length, fetal fibronectin or other instantaneous tests are not recommended during multiple pregnancies. | NS | NS | Antenatal care | Preterm labour | Screening |
| **Multiple Pregnancy** | Lithuanian Society of Obstetricians and Gynaecologists, Lithuanian Midwives Association | 2014 | 5.15.3 | For the prevention of preterm birth the following are s not recommended: ● bed rest mode at home or in the hospital; ● progesterone preparations; ● cervical suturing; ● oral tocolysis preparations | NS | NS | Antenatal care | Preterm labour | Interventions |
| **Multiple Pregnancy** | Lithuanian Society of Obstetricians and Gynaecologists, Lithuanian Midwives Association | 2014 | 5.15.4 | Steroid maturation of the fetus is prescribed only if there are signs of preterm birth or there are medical indications for premature termination of pregnancy. The indications for fetal lung maturation are the same as for single fetuses in case of pregnancy. The effects of steroids during multiple pregnancies are not as effective in reducing the incidence of respiratory distress syndrome as in a single pregnancy. | NS | NS | Antenatal care | Preterm labour | Interventions |

**Article Title:** Clinical practice guidelines for the antenatal management of dichorionic diamniotic twin pregnancies: a systematic review.

**Author names:**

Caroline O’Connor^1, 2*^, Emily O’Connor^1, 2, 3^, Sara Leitao^2, 3^, Shauna Barrett^4^, Keelin O’Donoghue^1, 2^

**Affiliations**

^1^ INFANT Research Centre, University College Cork, Cork, Ireland

^2^ Pregnancy Loss Research Group, Department of Obstetrics & Gynecology, University College Cork, Cork, Ireland

^3^ National Perinatal Epidemiology Center (NPEC), University College Cork, Cork, Ireland

^4^ Cork University Hospital Library, Cork University Hospital, Cork, Ireland

**Corresponding author:** *Caroline O’Connor

E-mail: carolineoconnor@ucc.ie
