# Supplementary material for: Clinical practice guidelines for the antenatal management of dichorionic diamniotic twin pregnancies: a systematic review
Source: BMC Pregnancy Childbirth. 2023 May 13;23:347. doi: 10.1186/s12884-023-05652-z (PMC10182673; doi:10.1186/s12884-023-05652-z)
Supplement: Supplementary file 9 — Additional file 9 [file 12884_2023_5652_MOESM9_ESM.docx]

| **Guideline title** | **Author** | **Year** | **Recommendation No.** | **Recommendation** | **Strength of Recommendation** | **Quality of evidence** | **Recommendation category specified within guideline** | **Category** | **Subcategory** | |
| --- | --- | --- | --- | --- | --- | --- | --- | --- | --- | --- |
| **Multifetal Gestations: Twin, Triplet, and Higher-Order Multifetal Pregnancies (Practice Bulletin No 231).** | ACOG | 2021 | NS | Women with uncomplicated dichorionic–diamniotic twin gestations can undergo delivery at 380/7–38 6/7weeks of gestation | NS | NS | None | Birth | Timing | |
| **Multifetal Gestations: Twin, Triplet, and Higher-Order Multifetal Pregnancies (Practice Bulletin No 231).** | ACOG | 2021 | NS | The optimal route of delivery in women with twin gestations depends on the type of twins, fetal presentations, gestational age, and experience of the clinician performing the delivery. A twin gestation in and of itself is not an indication for cesarean delivery. | NS | NS | None | Birth | Mode | |
| **Multifetal Gestations: Twin, Triplet, and Higher-Order Multifetal Pregnancies (Practice Bulletin No 231).** | ACOG | 2021 | NS | Women with diamniotic twin gestations whose presenting fetus is in a vertex position are candidates for a vaginal birth | NS | NS | None | Birth | Mode | |
| **Multifetal Gestations: Twin, Triplet, and Higher-Order Multifetal Pregnancies (Practice Bulletin No 231).** | ACOG | 2021 | NS | In diamniotic twin pregnancies at 32 0/7 weeks of gestation or later with a presenting fetus that is vertex, regardless of the presentation of the second twin, vaginal delivery is a reasonable option and should be considered, provided that an obstetrician with experience in managing a non-vertex presenting second twin is available | Level C | NS | None | Birth | Mode | |
| **Multifetal Gestations: Twin, Triplet, and Higher-Order Multifetal Pregnancies (Practice Bulletin No 231).** | ACOG | 2021 | NS | Women with one previous low transverse cesarean delivery, who are otherwise appropriate candidates for twin vaginal delivery, may be considered candidates for trial of labor after cesarean delivery. | Level B | NS | None | Birth | Mode | |
| **Clinical practice guideline: Management of multiple pregnancy** | HSE | 2012 | NS | Mode of twin delivery should be considered on the basis of individual case characteristics to include comorbidity, gestational age, availability of expertise in the management of vaginal twin birth, and patient preference. | NS | NS | Mode of delivery | Birth | Mode | |
| **Clinical practice guideline: Management of multiple pregnancy** | HSE | 2012 | NS | Where no contraindications exist, monochorionic or dichorionic twins may be considered for vaginal birth. Women should be informed that where the first twin delivers by the vaginal route, the prospect of requiring a caesarean section for delivery of the second twin is approximately 4%. | NS | NS | Mode of delivery | Birth | Mode |  |
| **Clinical practice guideline: Management of multiple pregnancy** | HSE | 2012 | NS | An obstetrician experienced in vaginal twin birth should be immediately available in the event of twin labour. | NS | NS | Mode of delivery | Birth | Mode |  |
| **Twin and Triplet Pregnancy: NG137** | NICE | 2019 | 1.9.9 | ﻿Offer planned birth at 37 weeks to women with an uncomplicated dichorionic diamniotic twin pregnancy. | NS | NS | When to offer planned birth | Birth | Timing |  |
| **Twin and Triplet Pregnancy: NG137** | NICE | 2019 | 1.9.11 | ﻿Offer an individual assessment to determine the timing of planned birth in women with any of the following: • a complicated twin or triplet pregnancy • a monochorionic triamniotic triplet pregnancy • a triplet pregnancy that involves a shared amnion. | NS | NS | When to offer planned birth | Birth | Timing |  |
| **Twin and Triplet Pregnancy: NG137** | NICE | 2019 | 1.9.12 | ﻿For women who decline planned birth at the timing recommended in recommendations 1.9.9 and 1.9.10, offer weekly appointments with the specialist obstetrician. At each appointment, offer an ultrasound scan and perform assessments of amniotic fluid level and doppler of the umbilical artery flow for each baby in addition to fortnightly fetal growth scans. | NS | NS | When to offer planned birth | Birth | Timing |  |
| **Twin and Triplet Pregnancy: NG137** | NICE | 2019 | 1.10.3 | ﻿Offer caesarean section to women if the first twin is not cephalic at the time of planned birth. | NS | NS | Mode of birth: DCDA | Birth | Mode |  |
| **Twin and Triplet Pregnancy: NG137** | NICE | 2019 | 1.10.4 | ﻿Offer caesarean section to women in established preterm labour between 26 and 32 weeks if the first twin is not cephalic. | NS | NS | Mode of birth: DCDA | Birth | Mode |  |
| **Twin and Triplet Pregnancy: NG137** | NICE | 2019 | 1.10.5 | ﻿Offer an individualised assessment of mode of birth to women in suspected, diagnosed or established preterm labour before 26 weeks. Take into account the risks of caesarean section (see NICE's guideline on preterm labour and birth) and the chance of survival of the babies. | NS | NS | Mode of birth: DCDA | Birth | Mode |  |
| **Twin pregnancy** | South Australian Perinatal Practice Guideline | 2018 | NS | Plan for vaginal birth where twin 1 is in the vertex position. | NS | NS | Summary of Practice Recommendations | Birth | Mode |  |
| **Twin pregnancy** | South Australian Perinatal Practice Guideline | 2018 | NS | Timing of birth is dependent on chorionicity: Monochorionic twin pregnancies should be offered elective birth from 36+0 weeks after a course of prophylactic corticosteroids; dichorionic twin pregnancies can be offered elective birth from 37+0 weeks. | NS | NS | Summary of Practice Recommendations | Birth | Timing |  |
| **Twin pregnancy** | South Australian Perinatal Practice Guideline | 2018 | NS | When appropriate obstetric experience is available, vaginal birth is the preferred mode of birth for all twin pregnancies that meet the following criteria: -Twins must be diamniotic - Twin I is cephalic - Twin II is not > 500g heavier than twin I - Neither twin has any evidence of fetal compromise requiring caesarean section. | NS | NS | Timing and mode of birth | Birth | Mode |  |
| **Twin pregnancy** | South Australian Perinatal Practice Guideline | 2018 | NS | Twin pregnancies with breech presentation of twin one or other major obstetric risk factors may require elective caesarean section at 38 weeks gestation. | NS | NS | Delivery | Birth | Mode |  |
| **FIGO Good clinical practice advice: management of twin pregnancy** | FIGO | 2019 | NS | In DC twins with discordant FGR, timing of delivery can be decided based on risk-benefit assessment. | NS | NS | Screening, diagnosis and management of fetal growth restriction | Birth | Timing |  |
| **FIGO Good clinical practice advice: management of twin pregnancy** | FIGO | 2019 | NS | DCDA twins should be offered elective delivery from 37+0 weeks’ gestation | NS | NS | Timing of birth in uncomplicated twin pregnancy | Birth | Timing |  |
| **FIGO Good clinical practice advice: management of twin pregnancy** | FIGO | 2019 | NS | For uncomplicated DCDA twins, if leading twin is cephalic, reasonable to aim for vaginal delivery. | NS | NS | Mode of delivery of twin pregnancies | Birth | Mode |  |
| **FIGO Good clinical practice advice: management of twin pregnancy** | FIGO | 2019 | NS | If twin one is not cephalic, CS is “probably” the safer option. | NS | NS | Mode of delivery of twin pregnancies | Birth | Mode |  |
| **AWMF 015-087 S2e Guideline Monitoring and Care of Twin Pregnancies** | AWMF | 2020 | 41 | The timing of delivery should be based on the evaluation of interval growth, fetal doppler findings and/or CTG, and if available, computerised CTG analysis | NS | EK | Intrauterine growth restriction | Birth | Timing |  |
| **AWMF 015-087 S2e Guideline Monitoring and Care of Twin Pregnancies** | AWMF | 2020 | 58 | Straightforward DC twins can be delivered between 37+0 and 38+0 weeks of pregnancy. | A | 1-, 1-, 2++, 2++, 2- | Time of birth for twin pregnancies | Birth | Timing |  |
| **AWMF 015-087 S2e Guideline Monitoring and Care of Twin Pregnancies** | AWMF | 2020 | 60 | Uncomplicated twins >32 weeks gestation with twin 1 in cephalic position, with no contraindications or growth discordance, can be delivered vaginally or by caesarean section. The chorionicity does not play a role in the mode of birth. | A | 1++, 1+, 1+, 1+, 2++, 2++, 2+, 2+, 1+ | Birth mode in twin pregnancies | Birth | Mode |  |
| **AWMF 015-087 S2e Guideline Monitoring and Care of Twin Pregnancies** | AWMF | 2020 | 61 | For the delivery of uncomplicated twins <32 weeks gestation with the first twin in cephalic position, the evidence is insufficient in order to be able to make a reliable recommendation. | D | 2-, 2- | Birth mode in twin pregnancies | Birth | Mode |  |
| **Tvillinger - ﻿håndtering af graviditet og fødsel (twins- handling pregnancy and childbirth)** | Sandbjerg | 2010 | NS | For DCDA pregnancies: In uncomplicated pregnancy to 38 full weeks And TV-A in the main position (VTX) .At birth: TV A: The fetus / child first born either vaginally or by section.TV B: The fetus / child born last either vaginally or by section | BC | NS | Recommendations for delivery | Birth | Mode |  |
| **Tvillinger - ﻿håndtering af graviditet og fødsel (twins- handling pregnancy and childbirth)** | Sandbjerg | 2010 | NS | In uncomplicated pregnancies to 38 full weeks and TV-A (lower body presentation) in the UK. Elective section is recommended. | BC | NS | Recommendations for delivery | Birth | Mode |  |
| **Tvillinger - ﻿håndtering af graviditet og fødsel (twins- handling pregnancy and childbirth)** | Sandbjerg | 2010 | NS | Preterm birth (<24 weeks) Vaginal birth independent of fetal presentation. Also at TV-A in the UK (lower body presentation). Preterm birth (<32 weeks) A cesarean section be considered independently of chorionicity, fetal presentation and judgment. Preterm birth (> 32 weeks <37 weeks) At TV-B in the (lower body presentation) and at the same time fetal estimate <1700 gr, a caesarean section can be considered. Otherwise, aim for a vaginal birth | BC | NS | Recommendations for delivery | Birth | Mode |  |
| **Tvillinger – Håndtering af fødslen (Twins- handling the birth)** | Dansk Selskab for Obstetrik og Gynækologi | 2020 | NS | If presenting twin is vertex and estimated fetal weight estimate (both) is> 1500 grand <4000 gr women are advised for vaginal twin birth - when no other contraindications for vaginal birth exist. | A | NS | Mode of delivery and criteria for vaginal twin birth at GA> 32 + 0 | Birth | Mode |  |
| **Tvillinger – Håndtering af fødslen (Twins- handling the birth)** | Dansk Selskab for Obstetrik og Gynækologi | 2020 | NS | TV-A (leading twin) non-vertex recommended elective cesarean section delivery | D | NS | Mode of delivery and criteria for vaginal twin birth at GA> 32 + 1 | Birth | Mode |  |
| **Tvillinger – Håndtering af fødslen (Twins- handling the birth)** | Dansk Selskab for Obstetrik og Gynækologi | 2020 | NS | Pregnancies complicated by discordant fetal growth and / or fetal growth restriction needs to be evaluated and guided on an individual basis in relation to mode of delivery· In twin pregnancies complicated by discordant fetal growth (> 25% difference),especially when TV-B (non leading twin)> TV-A (leading twin) and TV-B is in non-vertex presentation elective C / S delivery should be considered· Twin pregnancies complicated by IUGR and compromised fetus (es) should be delivered by C / S on the same indications as singletons | D | NS | Mode of delivery and criteria for vaginal twin birth at GA> 32 + 2 | Birth | Mode |  |
| **Tvillinger – Håndtering af fødslen (Twins- handling the birth)** | Dansk Selskab for Obstetrik og Gynækologi | 2020 | NS | Is is recommended that uncomplicated dichorionic twins are delivered electively from GA 37 + 0 and before GA 38 + 0, as this is associated with the lowest perinatal morbidity and mortality risk. | B | NS | Mode of delivery and criteria for vaginal twin birth at GA> 32 + 3 | Birth | Timing |  |
| **Tvillinger – Håndtering af fødslen (Twins- handling the birth)** | Dansk Selskab for Obstetrik og Gynækologi | 2020 | NS | Timing of elective premature delivery and decision on safest mode of delivery of the complicated twin pregnancy must be individualized. Concerns on risks and benefits of conservative management verses active management (delivery) should be assessed and balanced against the risks associated with prematurity | D | NS | Mode of delivery and criteria for vaginal twin birth at GA> 32 + 4 | Birth | Timing |  |
| **Tvillinger – Håndtering af fødslen (Twins- handling the birth)** | Dansk Selskab for Obstetrik og Gynækologi | 2020 | NS | For DC-twins of GA <32 + 0 wks vaginal birth is recommended when first twin is in the vertex presentation - the overall clinical situation is taken into consideration. | D | NS | Mode of delivery - preterm twins GA 24 + 0-31 + 6 | Birth | Mode |  |
| **Tvillinger – Håndtering af fødslen (Twins- handling the birth)** | Dansk Selskab for Obstetrik og Gynækologi | 2020 | NS | IUGR and / or discordant fetal growth should be considered a risk factor in itself; especially if TV-B> TV-A and should be taken into consideration when planning / recommending mode of delivery | D | NS | Mode of delivery - preterm twins GA 24 + 0-31 + 7 | Birth | Mode |  |
| **Tvillinger – Håndtering af fødslen (Twins- handling the birth)** | Dansk Selskab for Obstetrik og Gynækologi | 2020 | NS | If first twin is in a breech presentation or non-cephalic delivery by C / S is recommended. | C | NS | Mode of delivery - preterm twins GA 24 + 0-31 + 8 | Birth | Mode |  |
| **Management of multiple pregnancy** | SIGO, AOGOI, AGUI | 2016 | NS | If multiple pregnancy is not complicated and the US dating is correct, in order to reduce the risk of adverse outcomes, the completion of childbirth is recommended in DCDA twins at 38+0 weeks. | B | 2 | Timing and method of birth | Birth | Timing |  |
| **Management of multiple pregnancy** | SIGO, AOGOI, AGUI | 2016 | NS | In the case of diamniotic twins where both have cephalic presentation there are no contraindications to vaginal birth and caesarean section is indicated in the case of obstetric problems independent of chorionicity. | NS | NS | Timing and method of birth | Birth | Mode |  |
| **Management of multiple pregnancy** | SIGO, AOGOI, AGUI | 2016 | NS | In the case of diamniotic twins, beyond 32 weeks gestation in which the 2nd twin is not cephalic and with an EFW >1500g, vaginal delivery is a reasonable option subject to the fact that assistance is provided by an obstetrician with experience in the internal podalic manoeuvres/assisting with vaginal breech delivery. | B | 2 | Timing and method of birth | Birth | Mode |  |
| **Management of multiple pregnancy** | SIGO, AOGOI, AGUI | 2016 | NS | In women with a previous caesarean section and who are suitable candidates for vaginal delivery, labour is not contraindicated. | B | 4 | Timing and method of birth | Birth | Mode |  |
| **Twin pregnancies: guidelines for clinical practice from the French College of Gynaecologists and Obstetricians (CNGOF)** | Christophe Vayssiere | 2011 | NS | It is recommended to plan delivery of uncomplicated dichorionic diamniotic twin pregnancies from 38 weeks and before 40 weeks (Level C). | NS | NS | Level C | Birth | Timing |  |
| **Twin pregnancies: guidelines for clinical practice from the French College of Gynaecologists and Obstetricians (CNGOF)** | Christophe Vayssiere | 2011 | NS | Vaginal delivery should be performed by an obstetrician with experience in the vaginal delivery of twins (Professional Consensus) | NS | NS | Professional consensus | Birth | Mode |  |
| **Twin pregnancies: guidelines for clinical practice from the French College of Gynaecologists and Obstetricians (CNGOF)** | Christophe Vayssiere | 2011 | NS | There is no reason to recommend one type of delivery rather than another in twin pregnancies, regardless of gestational age at birth (Level C) | NS | NS | Level C | Birth | Mode |  |
| **Twin pregnancies: guidelines for clinical practice from the French College of Gynaecologists and Obstetricians (CNGOF)** | Christophe Vayssiere | 2011 | NS | In particular, there is no reason to recommend one type of delivery rather than another:-in a twin pregnancy with Twin 1 in cephalic presentation near term (Level B),-in a twin pregnancy with Twin 1 in breech presentation near term (Level B),-in a twin pregnancy in women with uterine scars (Level C),-in a twin pregnancy with Twin 1 in cephalic or breech presentation in women with preterm labour (Level C). | NS | NS | Level B, B, C, C | Birth | Mode |  |
| **Multiple Pregnancy** | Lithuanian Society of Obstetricians and Gynaecologists, Lithuanian Midwives Association | 2014 | 5.6 | Twin perinatal mortality begins to increase after 39 weeks of gestation. Therefore, even in uncomplicated pregnancies it is not recommended to continue the pregnancy for more than 39 weeks. | NS | NS | Antenatal care | Birth | Timing |  |
| **Multiple Pregnancy** | Lithuanian Society of Obstetricians and Gynaecologists, Lithuanian Midwives Association | 2014 | 6.1 | Recommended timing of delivery: Dichorionic, diamniotic twins, the course of pregnancy without complications 38 weeks. Dichorionic, diamniotic twins, fetal growth retardation 36-37 weeks. | NS | NS | Childbirth care | Birth | Timing |  |
| **Multiple Pregnancy** | Lithuanian Society of Obstetricians and Gynaecologists, Lithuanian Midwives Association | 2014 | 6.2.2 | Method of childbirth: The choice of method of childbirth is influenced by the duration of pregnancy, the duration of the fetus and the expected weight | NS | NS | Childbirth care | Birth | Mode |  |
| **Multiple Pregnancy** | Lithuanian Society of Obstetricians and Gynaecologists, Lithuanian Midwives Association | 2014 | 6.2.3.1 | If the pregnancy is uncomplicated and the presenting part of both fetuses is the head, a natural birth is recommended | 2B | NS | Childbirth care | Birth | Mode |  |
| **Multiple Pregnancy** | Lithuanian Society of Obstetricians and Gynaecologists, Lithuanian Midwives Association | 2014 | 6.2.3.2 | If only the first part of the first twin is the head, it is also recommended to give birth naturally. In this case, an unplanned caesarean section due to the second twin may be 4–10 percent cases. | NS | NS | Childbirth care | Birth | Mode |  |
| **Multiple Pregnancy** | Lithuanian Society of Obstetricians and Gynaecologists, Lithuanian Midwives Association | 2014 | 6.2.3.3 | If the pregnancy is between 32 and 38 weeks and the first part of the first twin is the head, caesarean section does not increase or decrease fetal or neonatal death risks and risks of neonatal morbidity compared to natural delivery | NS | NS | Childbirth care | Birth | Mode |  |
| **Multiple Pregnancy** | Lithuanian Society of Obstetricians and Gynaecologists, Lithuanian Midwives Association | 2014 | 6.2.3.4 | If the first part of the first twin is not the head, a caesarean section is performed | NS | NS | Childbirth care | Birth | Mode |  |
| **Multiple Pregnancy** | Lithuanian Society of Obstetricians and Gynaecologists, Lithuanian Midwives Association | 2014 | 6.2.4 | If a woman has had one caesarean section and the birth started spontaneously, it is possible to allow natural birth. A characteristic early sign of uterine rupture is an abnormal fetus heart rate, so continuous heart rate in both fetuses is essential monitoring. If such options are not available, it is better to complete the pregnancy with caesarean section | NS | NS | Childbirth care | Birth | Mode |  |
| **Multiple Pregnancy** | Lithuanian Society of Obstetricians and Gynaecologists, Lithuanian Midwives Association | 2014 | 6.2.5 | Other indications for a caesarean section: The head circumference of the second twin is 12 cm or more larger than that of the first. Unequal growth of twins. Maternal or fetal complications. Conjoined twins. | NS | NS | Childbirth care | Birth | Mode |  |

**Article Title:** Clinical practice guidelines for the antenatal management of dichorionic diamniotic twin pregnancies: a systematic review.

**Author names:**

Caroline O’Connor^1, 2*^, Emily O’Connor^1, 2, 3^, Sara Leitao^2, 3^, Shauna Barrett^4^, Keelin O’Donoghue^1, 2^

**Affiliations**

^1^ INFANT Research Centre, University College Cork, Cork, Ireland

^2^ Pregnancy Loss Research Group, Department of Obstetrics & Gynecology, University College Cork, Cork, Ireland

^3^ National Perinatal Epidemiology Center (NPEC), University College Cork, Cork, Ireland

^4^ Cork University Hospital Library, Cork University Hospital, Cork, Ireland

**Corresponding author:** *Caroline O’Connor

E-mail: carolineoconnor@ucc.ie
